# First synthesis of acylated nitrocyclopropanes

**DOI:** 10.3762/bjoc.19.67

**Published:** 2023-06-21

**Authors:** Kento Iwai, Rikiya Kamidate, Khimiya Wada, Haruyasu Asahara, Nagatoshi Nishiwaki

**Affiliations:** 1 School of Engineering Science, Kochi University of Technology, Tosayamada, Kami, Kochi 782-8502, Japan; 2 Research Center for Molecular Design, Kochi University of Technology, Kami, Kochi 782-8502, Japan; 3 Graduate School of Pharmaceutical Sciences, Osaka University, Yamadaoka 1-6, Suita, Osaka 565-0871, Japan

**Keywords:** acetoxyiodine, conjugate addition, dihydrofuran, nitroalkene, nitrocyclopropane

## Abstract

Although nitrocyclopropanedicarboxylic acid esters are widely used in organic syntheses, nitrocyclopropanes with an acyl group have not yet been synthesized. When adducts of β-nitrostyrene and 1,3-dicarbonyl compounds are treated with (diacetoxyiodo)benzene and tetrabutylammonium iodide, iodination occurs at the α-position of the nitro group, and the subsequent *O*-attack of the enol moiety leads to 2,3-dihydrofuran. Cyclopropane was successfully synthesized through *C*-attack as the acyl group became bulkier. The obtained nitrocyclopropane was transformed into furan upon treatment with tin(II) chloride via a ring-opening/ring-closure process.

## Introduction

**Scheme 1:**
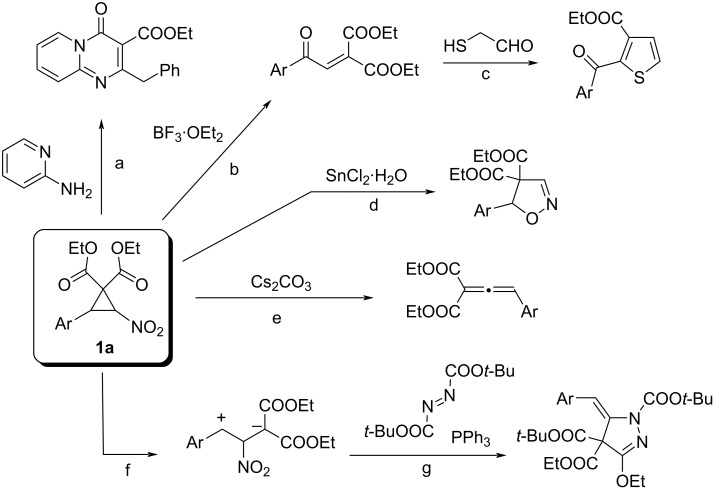
Versatile reactivities of cyclopropanes **1a**.

3-Arylated 2-nitrocyclopropane-1,1-dicarbonylic acid esters **1a** have recently attracted considerable attention from synthetic organic chemists. In addition to their polyfunctionality, their ring strain and electron deficiency lead to a wide variety of reactivities. Based on their electron-deficient nature, these compounds have been used as substrates in the reaction with dinucleophiles such as 2-aminopyridines, which affords pyrido[1,2-*a*]pyrimidinones through ring opening (Scheme 1, reaction a) [1]. Chemical transformations that take advantage of polyfunctionality are also possible. A six-membered ring forms between the aryl group and ester functionality through an intramolecular aza-Wittig reaction, yielding cyclopropane-fused 2-quinolones [2]. A nitro group not only activates substrates and stabilizes the α-anion as an electron-withdrawing group but also acts as a nucleophile, electrophile, and leaving group, exhibiting diverse reactivities [3]. For example, when esters **1a** are subjected to Lewis acid-induced denitration, highly electron-deficient enones (reaction b) [4] are obtained. The latter compounds are highly reactive and undergo reaction with, e.g., mercaptoacetaldehyde affording thiophenes (reaction c) [5] or with activated (hetero)aromatic compounds to give diarylated (oxoalkyl)malonates [6]. In the reaction using tin(II) chloride as the Lewis acid, the ring opening and nucleophilic attack of the nitro group occur, to produce functionalized isoxazolines (reaction d) [7]. In contrast, denitration under basic conditions generates highly reactive allenes (reaction e), which serve as synthetic intermediates for polyfunctionalized enynes [8]. The ring strain of the cyclopropane ring facilitates the cleavage of the C–C bond, and both cation and anion are stabilized by the adjacent phenyl group and ester functions, respectively (reaction f). These structural features enable the construction of a five-membered ring upon treatment with alkenes [9], diazo compounds (reaction g) [10], and nitriles [11].

**Scheme 2:**
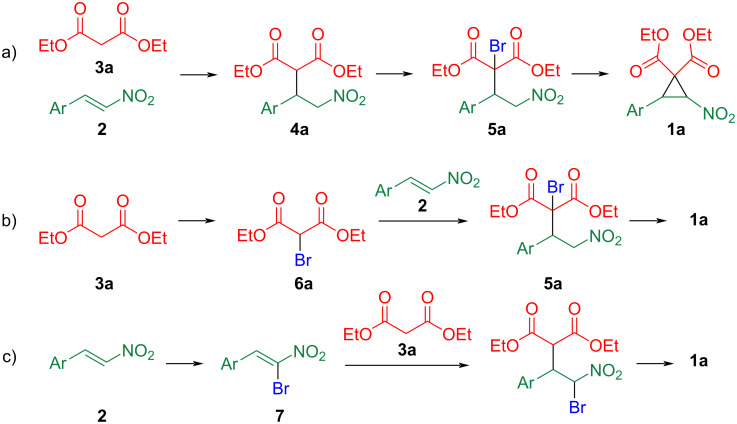
Preparative methods for cyclopropanedicarboxylates **1a**.

Several approaches are available for the synthesis of cyclopropanedicarboxylates **1a**, which consist of three steps: 1) conjugate addition, 2) halogenation, and 3) ring closure (Scheme 2). β-Nitrostyrene **2** serves as an appropriate acceptor for conjugate addition by diethyl malonate (**3a**) to afford adduct **4a**, in which the methine group flanked by two carbonyl groups is readily halogenated, and the subsequent intramolecular nucleophilic substitution by nitronic acid furnishes cyclopropane **1a** (method a) [1,4,12–13]. Halogenated malonate **6a** [7,14–17] and nitrostyrene **7** [18] can also be used as substrates in this protocol (methods b and c, respectively). In these methods, diesters are mostly used as 1,3-dicarbonyl compounds, with acetylacetone **3b** used in only three cases [12–13,19], to the best of our knowledge. Extending beyond Scheme 2, only two syntheses of nitrocyclopropanes containing cyclic keto esters or diketones have been reported [20–21]. These findings indicate that the synthesis of nitrocyclopropanes with an acyl group is quite difficult, although they would be very useful in synthetic chemistry, if available. In this study, we investigated the synthesis of nitrocyclopropanes using keto esters **3c**–**f** and diketones **3b** and **3g** instead of diester **3a** as the starting 1,3-dicarbonyl compounds.

## Results and Discussion

**Scheme 3:**
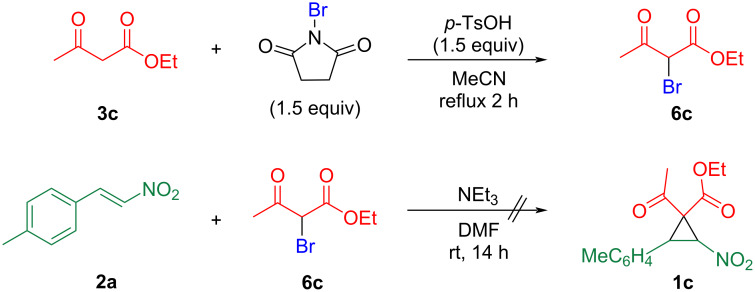
Bromination of ethyl acetoacetate (**3c**) and reaction with nitrostyrene **2a**.

We commenced our study using ethyl acetoacetate (**3c**) as starting 1,3-dicarbonyl compound according to method b (Scheme 2). After heating an acetonitrile solution of **3c**, *N*-bromosuccinimide, and *p*-toluenesulfonic acid [7], α-bromoacetoacetate **6c** was isolated with a 44% yield after purification of the reaction mixture by silica gel column chromatography (Scheme 3). In the subsequent reaction of the α-brominated product **6c** with nitrostyrene **2a** in the presence of triethylamine, the complete consumption of **2a** was confirmed, however, the reaction mixture was complicated, and the desired cyclopropane **1c** was not detected (Scheme 3).

Next, method a (Scheme 2) was employed to synthesize nitrocyclopropanes **1** possessing an acyl group. Keto esters **3c–f** and diketones **3b** and **3g** underwent conjugate addition to nitrostyrene **2a** to afford the corresponding adducts **4b**–**g** with moderate yields (Table 1). For unsymmetrical substrates, almost equal amounts of diastereomers were formed. Whereas **4b** was isolated by recrystallization of the reaction mixture from ethanol, the other adducts **4c**–**g** were easily isolated by column chromatography.

**Table 1:** Michael addition of 1,3-dicarbonyl compounds **3b**–**g** to nitrostyrene **2a**.

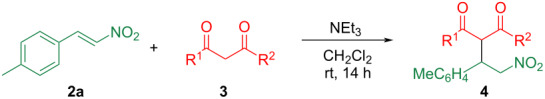					

Entry	R^1^	R^2^		Yield/%	dr^a^

1	Me	OEt	**c**	74	58:42
2	Et	OMe	**d**	55	54:46
3	iPr	OMe	**e**	54	53:47
4	Ph	OEt	**f**	67	54:46
5	Me	Me	**b**	56	—
6	Me	Ph	**g**	66	57:43

^a^Diastereisomeric ratio determined by ^1^H NMR.

**Scheme 4:**
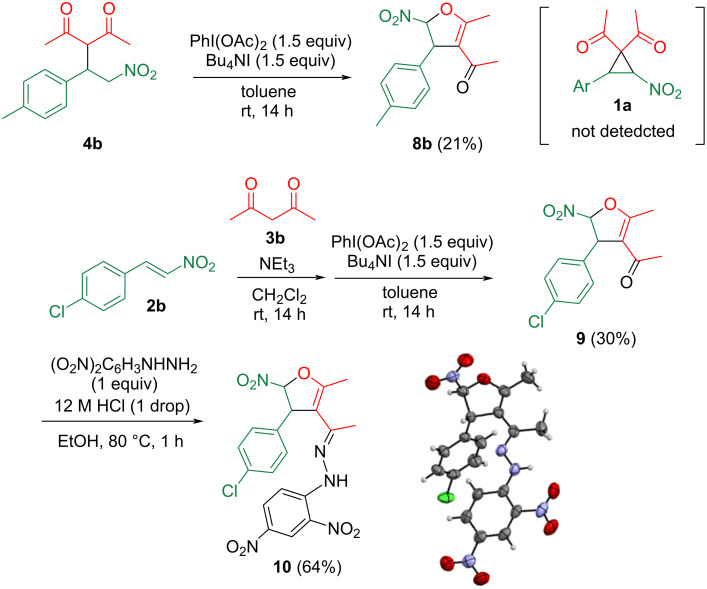
Reaction of **4b** with (diacetoxyiodo)benzene (top); structural determination of product **9** (bottom).

For comparison with previously reported results, adduct **4b**, derived from acetylacetone (**3b**), was subjected to cyclopropanation according to a method described in the literature [13]. To a solution of adduct **4b** in toluene, (diacetoxyiodo)benzene and tetrabutylammonium iodide were added, and the resulting mixture was stirred at room temperature for 14 h. Unexpectedly, from the reaction mixture, compound **8b** was isolated after column chromatography with 21% yield instead of the desired cyclopropane **1b** (Scheme 4).

The NMR data for the ring protons of product **8b** and compound **1b’** are listed in Table 2. Although the benzene ring in compound **8b** is methyl-substituted, a ^1^H NMR spectrum similar to those in the literature was observed [12–13,19], indicating that both compounds have the same framework. Surprisingly, the coupling constants were considerably smaller than those of **1a** (Ar = MeC_6_H_4_), and the same tendency was previously found in the literature [12–13]. However, a reasonable explanation was not given for the different coupling constants between diester **1a** and diketone **1b’**. In the ^13^C NMR spectrum of diester **1a**, two separate signals of carbonyl groups were observed at 163.2 and 163.3 ppm, indicating that the two ester functionalities were not equivalent. Moreover, the spectrum of compound **8b** revealed only a single signal of a carbonyl carbon at 193.2 ppm, and a signal of a quaternary carbon at 167.0 ppm, which could not be assigned. These spectral data prompted us to reconsider the structure of compound **8b**.

**Table 2:** Comparison of the ^1^H NMR data of ring protons for compounds **1a**, **1b’**, and product **8b**.

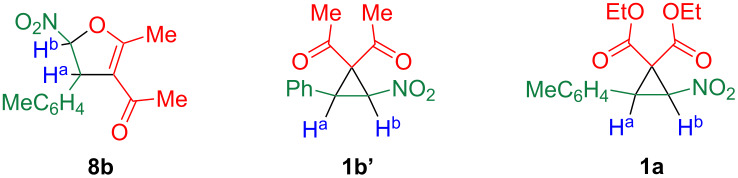									

Ref.		Chemical shift/ppm		Coupling constant	Ref.		Chemical shift/ppm		Coupling constant
				
		H^a^	H^b^	*J* [Hz]			H^a^	H^b^	*J* [Hz]

	**8b**	4.61 (br s)	5.71 (br s)	–					
[12]	**1b’**	4.66 (d)	5.77 (d)	2.0	[12]	**1a**	4.17 (d)	5.39 (d)	6.0
[13]	**1b’**	4.66 (d)	5.74 (d)	1.8	[13]	**1a**	4.15 (d)	5.38 (d)	6.0
[19]	**1b’**	4.59 (s)	5.67 (s)	–	[7]	**1a**	4.15 (d)	5.37 (d)	5.9

The reaction of chlorinated nitrostyrene **2b** with acetylacetone (**3b**) was in the same way, and the obtained product **9** was converted to the 2,4-dinitrophenylhydrazone **10** to facilitate the crystallization for X-ray crystallography, which showed that a 2,3-dihydrofuran framework had formed (Scheme 4, bottom). Hence, we clarified that product **8b** was not the desired cyclopropane, but a dihydrofuran, so the cyclopropane **1b’** reported in the literature [12–13,19] is presumably incorrect [22]. In the cases of donor–acceptor cyclopropanes possessing an electron-donating group such as an alkoxy or amino group, ring expansion caused by an intramolecular attack of nitro oxygen occurs, leading to five-membered cyclic nitronates [23]. To the contrary, such reaction was not observed at all, which is presumably due to the lower electron-donating ability of the benzene ring.

The other adducts **4c**–**g** were subjected to the cyclization under the same conditions (Table 3). When the adduct of ethyl acetoacetate (**4c**) was used, dihydrofuran **8c** was obtained as the main product, with small amounts of cyclopropane **1c** (Table 3, entry 1).

**Table 3:** Cyclization of other adducts **4**.

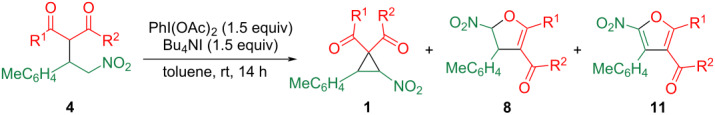							

Entry	R^1^	R^2^		Yield [%]			Recovery of **4**/%
		
				**1**	**8**	**11**	

1	Me	OEt	**c**	8	39	0	0
2	Et	OMe	**d**	12	44	trace	0
3	iPr	OMe	**e**	24	36	9	0
4	Ph	OEt	**f**	56	9	17	0
5	Me	Me	**b**	0	21	0	17
6	Me	Ph	**g**	5	30^a^	0	7

^a^Total yield of two isomers **8g** (14%) and **8g’** (R^1^ = Ph, R^2^ = Me, 16%).

With a bulkier acyl group the yield of **1** was higher, and the yield increased up to 56% in the reaction using adduct **4f**, which was derived from ethyl benzoylacetate (**3f**) (Table 3, entries 1–4). The yield of aromatized furans **8** also increased with an increase in the bulkiness of the acyl group. A similar tendency was observed when adducts **4b** and **4g** derived from diketones **3b** and **3g** were reacted in the same way (Table 3, entries 5 and 6).

**Figure 1:**
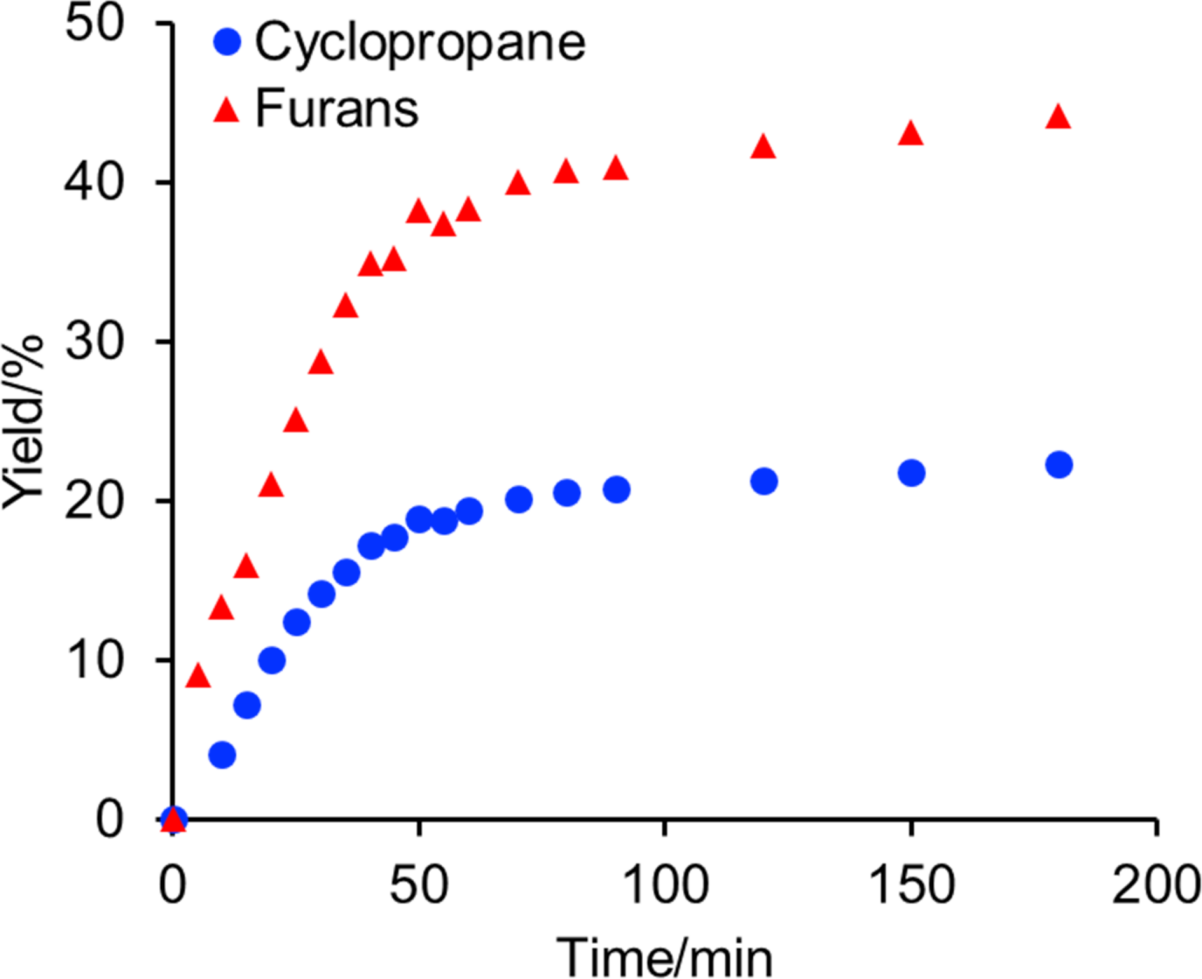
Monitoring the cyclization reaction using **4e** by ^1^H NMR.

To obtain insight into the two cyclization modes, the reaction of **4e** was monitored by ^1^H NMR in 5 min intervals (Figure 1). In Figure 1, the red triangles are the total yields of furans **8e** and **11e**. The yields of cyclopropane **1e** and furans **8e** and **11e** increased with increasing reaction time, without disturbing the shape of the graph. In addition, we confirmed that isolated cyclopropane **1e** did not change upon treatment with (diacetoxyiodo)benzene and tetrabutylammonium iodide. These findings indicate that no equilibrium existed between cyclopropane **1e** and dihydrofuran **8e**, and that these products were competitively formed.

**Scheme 5:**
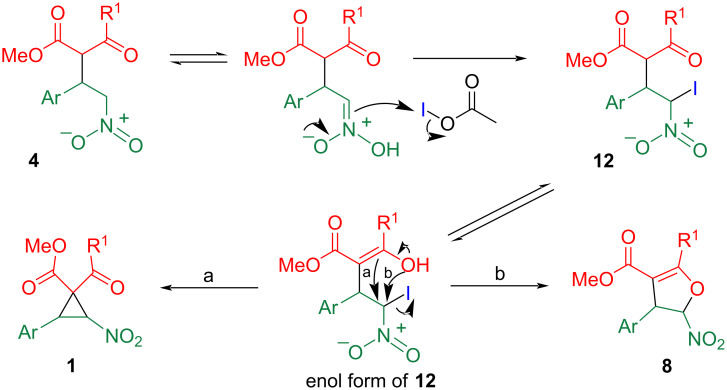
A plausible mechanism for formation of cyclopropane **1** and dihydrofuran **8**.

A plausible mechanism explaining the experimental results is illustrated in Scheme 5. In this reaction, acetoxyiodine serves as the active species [13,24]. Nitronic acid, one of the tautomers of **4**, attacks the acetoxyiodine to afford α-iododerivative **12**. After the carbonyl moiety tautomerized to the enol form, a *C*-attack (path a) furnishes cyclopropane **1**, and an *O*-attack (path b) furnishes dihydrofuran **8**. When the R^1^ group becomes bulkier, the hydroxy group may be far from the reaction site because of the steric repulsion in the stable conformation. Another possibility is that the bulky substituent may prevent the attack of other reagents and suppress the decomposition of the nitrocyclopropane framework.

**Scheme 6:**
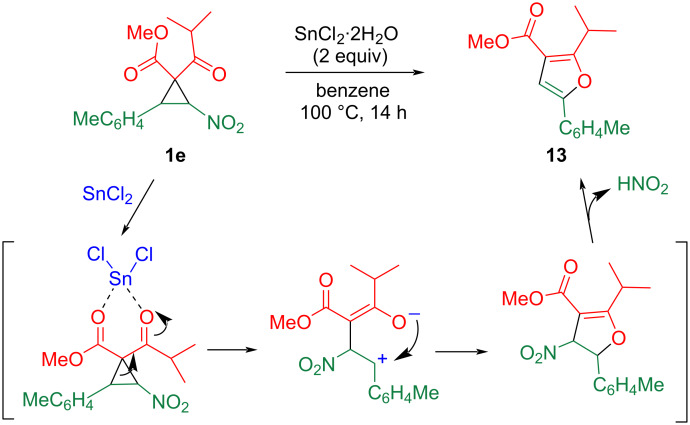
Tin(II)-mediated ring expansion of nitrocyclopropane **1e**.

The chemical transformation of cyclopropane **1e** was investigated. When a solution of **1e** and tin(II) chloride in benzene was heated at 100 °C for 14 h, successive ring-opening/ring-closure proceeded, to produce furan **13** with a 46% yield (Scheme 6). The coordination of two carbonyl groups to the tin species facilitated the ring opening of the cyclopropane ring to afford betaine [7], then the oxygen atom of the enolate attacked the benzyl cation to construct a five-membered ring. The subsequent elimination of nitrous acid, accompanied by aromatization, yielded furan **13**. In addition to the stepwise mechanism, a concerted ring-expansion can be also acceptable [25].

## Conclusion

Although nitrocyclopropanedicarboxylic acid esters **1a** have been used in organic syntheses, nitrocyclopropanes possessing an acyl group are unknown, except for a derivative of cyclic diketone [20–21]. Although diacetyl derivative **13** has been reported in some studies [12–13,19], we corrected its structure to dihydrofuran **8**. This is the first report of the synthesis of acylated nitrocyclopropanes **1**. After the conjugate addition of the 1,3-dicarbonyl compound **3** to nitrostyrene **2** and the α-iodination of the adduct **4**, two cyclization modes became possible owing to the ambident property of enol **12**. Dihydrofuran **8** was formed in the case of an *O*-attack, and nitrocyclopropane **1** was formed in the case of a *C*-attack. Furthermore, the latter cyclization predominantly proceeded as the acyl group became bulkier. The polyfunctionality of products **1** and **8** facilitate further chemical conversion [22]. Nitrocyclopropane **1e** was converted into furan **13** by treatment with tin(II) chloride. The findings obtained herein will be useful for researchers studying organic syntheses using functionalized cyclopropanes.

## Experimental

### General

All reagents were purchased from commercial sources and used without further purification. ^1^H and^13^C NMR spectra were recorded on Bruker DPX-400 and JEOL JMN-ECZ400S spectrometers (400 MHz and 100 MHz, respectively) in CDCl_3_ using TMS as an internal standard. The assignments of the ^13^C NMR signals were performed by DEPT experiments. IR spectra were recorded on a JASCO FT/IR-4200 spectrometer equipped with an ATR detector. High-resolution mass spectra were obtained on AB SCIEX Triplet TOF 4600 and Bruker Compact mass spectrometers. All measurements were made on a Rigaku AFC7R diffractometer with graphite monochromatized Mo Kα radiation. Melting points were recorded on an SRS-Optimelt automated melting point system and were uncorrected.

### Preparation of nitrostyrenes **2**

Nitrostyrenes **2** were prepared according to the literature [26]. To a solution of ammonium acetate (2.63 g, 36 mmol) in acetic acid (10 mL), were added 4-methylbenzaldehyde (2 mL, 15 mmol) and nitromethane (5.3 mL, 98 mmol), and the resultant mixture was heated at 100 °C for 6 h. After adjusting the pH value to 7 by 2 M sodium hydroxide (40 mL, 80 mmol), the mixture was extracted with ethyl acetate (30 mL × 3), and the organic layer was washed with brine (30 mL × 1), dried over magnesium sulfate, and concentrated to afford nitrostyrene **2a** (17.4 g, 71%, mp 56–58 °C) as a yellow solid. Nitrostyrene **2b** (mp 112–114 °C) was prepared in the same way. The structure was confirmed by comparison with those reported in the literature [26].

### Conjugate addition of 1,3-dicarbonyl compound **3** to nitrostyrene **2**

Adduct **4** was prepared according to the literature [27]. To a solution of nitrostyrene **2a** (978 mg, 6 mmol) in dichloromethane (8 mL), were added ethyl benzoylacetate (**3f**, 1.04 mL, 6 mmol) and triethylamine (84 μL, 0.6 mmol), and the resultant solution was stirred at room temperature for 14 h. After removal of the solvent under reduced pressure, the residual pale-yellow solid was extracted with hot hexane (20 mL × 4). The hexane was concentrated, and the residual yellow oil was subjected to column chromatography on silica gel to afford ethyl 2-benzoyl-3-(4-methylphenyl)-4-nitrobutanoate (**4f**) [28] (eluted with hexane/ethyl acetate 95:5, 1.43 g, 4.02 mmol, 67%) as a pale-yellow oil. Major isomer ^1^H NMR (400 MHz, CDCl_3_) δ 0.92 (t, *J* = 7.2 Hz, 3H), 4.17 (q, *J* = 1.8, 7.2 Hz, 2H), 4.48–4.37 (m, 1H), 4.81–4.72 (m, 1H), 4.97–4.88 (m, 2H), 7.09 (d, *J* = 8.0 Hz, 2H), 7.18 (d, *J* = 8.0 Hz, 2H), 7.48 (dd, *J* = 7.6, 7.2 Hz, 2H), 7.61 (t, *J* = 7.6 Hz, 1H), 8.05 (d, *J* = 7.2 Hz, 2H); ^13^C NMR (100 MHz, CDCl_3_) δ 13.6 (CH_3_), 21.1 (CH_3_), 42.8 (CH), 57.1 (CH), 61.9 (CH_2_), 78.1 (CH_2_), 128.1 (CH), 128.6 (CH), 128.9 (CH), 128.9 (CH), 133.8 (C), 134.2 (CH), 135.9 (C), 138.0 (C), 167.8 (C), 192.9 (C). Minor isomer ^1^H NMR (400 MHz, CDCl_3_) δ 1.17 (dd, *J* = 7.2, 7.2 Hz, 3H), 3.88 (dq, *J* = 7.2 Hz, 1H), 3.88 (dq, *J* = 7.2 Hz, 1H), 4.48–4.37 (m, 1H), 4.81–4.72 (m, 1H), 4.97–4.88 (m, 2H), 7.02 (d, *J* = 8.2 Hz, 2H), 7.11 (d, *J* = 7.8 Hz, 2H), 7.41 (dd, *J* = 7.6, 7.2 Hz, 2H), 7.55 (t, *J* = 7.6 Hz, 1H), 7.86 (d, *J* = 7.2 Hz, 2H); ^13^C NMR (100 MHz, CDCl_3_) δ 13.9 (CH_3_), 21.0 (CH_3_), 42.8 (CH), 56.5 (CH), 62.2 (CH_2_), 78.1 (CH_2_), 127.8 (CH), 128.7 (CH), 129.6 (CH), 129.6 (CH), 133.2 (C), 133.8 (CH), 135.1 (C), 137.8 (C), 167.0 (C), 192.7 (C).

Although the adduct was obtained as a mixture of diastereomeric isomers (54:46), these isomers were subjected to subsequent cyclization without separation. When other 1,3-dicarbonyl compounds **3** or nitrostyrene **2b** were used, the reaction was conducted in the same way.

### Cyclization of adduct **4**

Cyclization was conducted according to the literature [13]. To a solution of adduct **4f** (593 mg, 1.67 mmol) in toluene (7 mL), were added (diacetoxyiodo)benzene (539 mg, 2.5 mmol) and tetrabutylammonium iodide (618 mg, 2.5 mmol), and the resultant mixture was stirred at room temperature for 14 h. The solution was subjected to column chromatography on silica gel to afford ethyl 4,5-dihydro-4-(4-methylphenyl)-5-nitro-2-phenylfuran-3-carboxylate (**8f**) (eluted with hexane/ethyl acetate 95:5, 53 mg, 0.15 mmol, 9%) as a pale-yellow oil, ethyl 1-benzoyl-2-(4-methylphenyl)-3-nitrocyclopropanecarboxylate (**1f**) (eluted with hexane/ethyl acetate 90:10, 330 mg, 0.94 mmol, 56%) as a yellow oil, and ethyl 4-(4-methylphenyl)-5-nitro-2-phenylfuran-3-carboxylate (**11f**) (eluted with hexane/ethyl acetate 90:10, 100 mg, 0.28 mmol, 17%) as a yellow oil, respectively.

**Ethyl 1-benzoyl-2-(4-methylphenyl)-3-nitrocyclopropanecarboxylate (1f):** Major isomer: ^1^H NMR (400 MHz, CDCl_3_) δ 0.82 (t, *J* = 7.1 Hz, 3H), 2.34 (s, 3H), 3.90 (q, *J* = 7.1 Hz, 2H), 4.34 (d, *J* = 5.8 Hz, 1H), 5.73 (d, *J* = 5.8 Hz, 1H), 7.16 (d, *J* = 7.9 Hz, 2H), 7.24 (d, *J* = 7.9 Hz, 2H), 7.48 (dd, *J* = 7.5, 7.8 Hz, 2H), 7.59 (t, *J* = 7.5 Hz, 1H), 7.96 (d, *J* = 7.8 Hz, 2H); ^13^C NMR (100 MHz, CDCl_3_) δ 13.5 (CH_3_), 21.1 (CH_3_), 36.5 (CH), 49.8 (C), 62.7 (CH_2_), 67.8 (CH), 127.5 (C), 128.2 (CH), 128.3 (CH), 129.1 (CH), 129.5 (CH), 134.1 (CH), 135.1 (C), 138.3 (C), 164.3 (C), 186.6 (C); IR (ATR): 1362, 1557, 1682, 1695 cm^−1^; HRESIMS–TOF (*m*/*z*): [M + H]^+^ calcd for C_20_H_19_NO_5_, 354.1336; found, 354.1328. Minor isomer: ^1^H NMR (400 MHz, CDCl_3_) δ 0.99 (t, *J* = 7.1 Hz, 3H), 2.20 (s, 3H), 4.11 (q, *J* = 7.1 Hz, 2H), 4.44 (d, *J* = 6.2 Hz, 1H), 5.73–5.72 (overlapped, 1H), 7.00 (d, *J* = 8.0 Hz, 2H), 7.07 (d, *J* = 8.0 Hz, 2H), 7.37 (dd, *J* = 7.7, 7.8 Hz, 2H), 7.79 (d, *J* = 7.8 Hz, 2H); ^13^C NMR (100 MHz, CDCl_3_) δ 13.5 (CH_3_), 21.0 (CH_3_), 38.8 (CH), 52.4 (C), 63.1 (CH_2_), 66.2 (CH), 126.9 (C), 127.6 (CH), 128.6 (CH), 128.6 (CH), 129.5 (CH), 133.8 (CH), 135.6 (C), 138.4 (C), 164.8 (C), 187.8 (C).

**Ethyl 4,5-dihydro-4-(4-methylphenyl)-5-nitro-2-phenylfuran-3-carboxylate (8f): **^1^H NMR (400 MHz, CDCl_3_) δ 1.08 (dd, *J* = 7.2, 7.2 Hz, 3H), 4.02 (dq, *J* = 7.2, 10.8 Hz, 1H), 4.06 (dq, *J* = 7.2, 10.8 Hz, 1H), 4.80 (d, *J* = 1.7 Hz, 1H), 5.86 (d, *J* = 1.7 Hz, 1H), 7.21–7.20 (br s, 4H), 7.54–7.46 (m, 3H), 8.09–8.07 (m, 2H); ^13^C NMR (100 MHz, CDCl_3_) δ 13.9 (CH_3_), 21.1 (CH_3_), 57.0 (CH), 60.6 (CH_2_), 107.7 (C), 108.9 (CH), 127.0 (CH), 127.7 (C), 128.0 (CH), 129.9 (CH), 130.0 (CH), 134.9 (C), 138.3 (C), 162.7 (C), 163.5 (C); IR (ATR): 1371, 1572, 1697 cm^−1^; HRESIMS–TOF (*m*/*z*): [M + H]^+^ calcd for C_14_H_15_NO_4_, 354.1336; found, 354.1324.

**Ethyl 4-(4-methylphenyl)-5-nitro-2-phenylfuran-3-carboxylate (11f): **^1^H NMR (400 MHz, CDCl_3_) δ 0.99 (t, *J* = 7.2 Hz, 3H), 2.42 (s, 3H), 4.12 (q, *J* = 7.2 Hz, 2H), 7.26 (d, *J* = 6.8 Hz, 2H), 7.32 (d, *J* = 6.8 Hz, 2H), 7.52–7.47 (m, 3H), 7.93 (dd, *J* = 7.2, 1.6 Hz, 2H); ^13^C NMR (100 MHz, CDCl_3_) δ 13.5 (CH_3_), 21.4 (CH_3_), 61.6 (CH_2_), 118.2 (C), 125.6 (C), 127.3 (C), 128.4 (CH), 128.7 (CH), 128.9 (CH), 129.2 (CH), 131.2 (CH), 139.3 (C), 154.9 (C), 162.5 (C), Two signals were lacked presumably due to overlapping. IR (ATR): 1359, 1593, 1730 cm^−1^; HRESIMS–TOF (*m*/*z*): [M + Na]^+^ calcd for C_19_H_19_NO_6_, 374.0999; found, 374.1000.

## Supporting Information

File 1 Spectral data for **1, 4, 8, 10, 13** and NMR charts (^1^H and ^13^C NMR), and information of X-ray analysis for **10**.

File 2 Crystallographic information file for compound **10**.

File 3 CheckCIF/PLATON report for compound **10**.
