# Supplementary material for: First synthesis of acylated nitrocyclopropanes
Source: Beilstein J Org Chem. 2023 Jun 21;19:892–900. doi: 10.3762/bjoc.19.67 (PMC10291240; doi:10.3762/bjoc.19.67)
Supplement: File 1 — Spectral data for 1, 4, 8, 10, 13 and NMR charts (1H and 13C NMR), and information of X-ray analysis for 10. [file Beilstein_J_Org_Chem-19-892-s001.pdf]

BEILSTEIN JOURNAL OF ORGANIC CHEMISTRY

Supporting Information

for

First synthesis of acylated nitrocyclopropanes

Kento Iwai, Rikiya Kamidate, Khimiya Wada, Haruyasu Asahara and Nagatoshi Nishiwaki

Beilstein J. Org. Chem. **2023,** *19,* 892–900. doi:10.3762/bjoc.19.67

Spectral data for 1, 4, 8, 10, 13 and NMR charts (^{1}H and ^{13}C NMR), and information of X-ray analysis for 10

License and Terms: This is a supporting information file under the terms of the Creative Commons Attribution License (https://creativecommons.org/licenses/by/4.0). Please note that the reuse, redistribution and reproduction in particular requires that the author(s) and source are credited and that individual graphics may be subject to special legal provisions.
The license is subject to the *Beilstein Journal of Organic Chemistry* terms and conditions: (https://www.beilstein-journals.org/bjoc/terms)

Table of contents

Synthesis of brominated ethyl acetoacetate **6c** S2
Spectral data of **4** S2
Spectral data of **1** S5
Spectral data of **8** and **11** S6
Information about X-ray analysis and spectral data of **10** S9
Synthesis of **10** via product **9** S10
Spectral data of **13** S11
^{1}H, ^{13}C, DEPT NMR of adducts **4** S12
^{1}H, ^{13}C, DEPT NMR of 1,1-dicarbonylated nitrocyclopropanes **1** S23
^{1}H, ^{13}C, DEPT NMR of 3-carbonylayed 4,5-dihydro-5-nitrofurans **8** and **11** S30
^{1}H, ^{13}C, DEPT NMR of product **9** and hydrazone **10** S44
^{1}H, ^{13}C, DEPT NMR of 2-isopropyl-5-(4-methylphenyl)furan-3-carboxylate (**13**) S47
References S48

Synthesis of brominated ethyl acetoacetate 6c

Bromination of ethyl acetoacetate (**3c**) was conducted using a modified method of the literature [1]. To a solution of *p*-toluenesulfonic acid monohydrate (5.7 g, 30 mmol) in acetonitrile (200 mL), were added *N*-bromosuccinimide (5.34 g, 30 mmol) and ethyl acetoacetate (**3c**, 2.6 g, 20 mmol). The resulting solution was heated under reflux for 2 h and concentrated under reduced pressure. The residue was dissolved in dichloromethane (200 mL), and the organic layer was washed with water (50 mL × 3), dried over magnesium sulfate, and concentrated. The residue was chromatographed on silica gel (dichloromethane) to afford brominated product **6c** (1.84 g, 8.8 mmol, 44%) as a colorless liquid. ^{1}H NMR (400 MHz, $CDCl_3$) δ 1.31 (t, *J* = 7.2 Hz, 3H), 2.45 (s, 3H), 4.26 (q, *J* = 7.2 Hz, 2H), 4.76 (s, 1H).

Spectral data for 4

3-[1-(4-Methylphenyl)-2-nitroethyl]-2,4-pentanedione (4b) [2]

Colorless solid. ^{1}H NMR (400 MHz, $CDCl_3$) δ 1.94 (s, 3H), 2.28 (s, 3H), 2.31 (s, 3H), 4.23–4.17 (m, 1H), 4.35 (d, *J* = 10.7 Hz, 1H), 4.61–4.59 (m, 2H), 7.06 (d, *J* = 8.0 Hz, 2H), 7.13 (d, *J* = 8.0 Hz, 2H); ^{13}C NMR (100 MHz, $CDCl_3$) δ = 21.0 (CH_3), 29.4 (CH_3), 30.4 (CH_3), 42.5 (CH), 70.9 (CH), 78.4 (CH_2), 127.8 (CH), 130.0 (CH), 132.9 (C), 138.4 (C), 201.0 (C), 201.9 (C).

Ethyl 2-[1-(4-methylphenyl)-2-nitroethyl]-3-oxobutanoate (4c) [3]

Pale-yellow oil (*dr* = 58/42). Major isomer: ^{1}H NMR (400 MHz, $CDCl_3$) δ 1.04 (t, *J* = 7.2 Hz, 3H), 2.30 (s, 6H), 3.98 (q, *J* = 7.2 Hz, 2H), 4.19–4.08 (m, 2H), 4.73 (d, *J* = 6.0 Hz, 2H), 7.12–7.06 (m, 4H); ^{13}C NMR (100 MHz, $CDCl_3$) δ 13.7 (CH_3), 21.0 (CH_3), 30.2 (CH_3), 42.0 (CH), 61.7 (CH), 61.9 (CH_2), 78.0 (CH_2), 127.8 (CH), 129.6 (CH), 133.3 (C), 138.1 (C), 166.9 (C), 201.3 (C). Minor isomer: ^{1}H NMR (400 MHz, $CDCl_3$) δ 1.28 (t, *J*

= 7.2 Hz, 3H), 2.05 (s, 3H), 2.30 (s, 3H), 4.19–4.08 (m, 2H), 4.23 (q, *J* = 7.2 Hz, 2H), 4.81–4.78 (m, 2H), 7.12–7.06 (m, 4H); ^{13}C NMR (100 MHz, $CDCl_3$) δ 14.0 (CH_3), 21.0 (CH_3), 30.0 (CH_3), 42.3 (CH), 62.1 (CH), 62.1 (CH_2), 77.5 (CH_2), 127.8 (CH), 129.8 (CH), 133.4 (C), 137.9 (C), 167.6 (C), 200.5 (C).

Methyl 2-[1-(4-methylphenyl)-2-nitroethyl]-3-oxopentanoate (4d)

Pale-yellow solid (*dr* = 54/46), mp 62–63 °C. Major isomer: ^{1}H NMR (400 MHz, $CDCl_3$) δ 1.06 (dd, *J* = 7.2, 7.2 Hz, 3H), 2.30 (s, 3H), 2.44 (dq, *J* = 7.2, 18.4 Hz, 1H), 2.66 (dq, *J* = 7.2, 18.4 Hz, 1H), 4.11 (d, *J* = 9.6 Hz, 1H), 4.23–4.16 (m, 1H), 4.86–4.75 (m, 2H), 7.06 (d, *J* = 8.0 Hz, 2H), 7.10 (d, *J* = 8.0 Hz, 2H); ^{13}C NMR (100 MHz, $CDCl_3$) δ 7.4 (CH_3), 21.0 (CH_3), 36.8 (CH_2), 42.2 (CH), 52.7 (CH_3), 61.0 (CH), 77.7 (CH_2), 127.6 (CH), 129.7 (CH), 133.5 (C), 138.0 (C), 167.6 (C), 204.2 (C); HRMS (ESI-TOF) calcd. for $C_{15}H_{19}NO_5$ [$M+H^+$] 294.1336, found: 294.1323. Minor isomer: ^{1}H NMR (400 MHz, $CDCl_3$) δ 0.85 (dd, *J* = 7.2, 7.2 Hz, 3H), 2.15 (dq, *J* = 7.2, 18.4 Hz, 1H), 2.29 (s, 3H), 2.44 (dq, *J* = 18.4, 7.2 Hz, 1H), 4.01 (d, *J* = 9.6 Hz, 1H), 4.23–4.16 (m, 1H), 4.86–4.75 (m, 2H), 7.06 (d, *J* = 8.0 Hz, 2H), 7.10 (d, *J* = 8.0 Hz, 2H); ^{13}C NMR (100 MHz, $CDCl_3$) δ 7.3 (CH_3), 21.0 (CH_3), 40.0 (CH_2), 42.4 (CH), 60.8 (CH), 52.9 (CH_3), 77.9 (CH_2), 127.7 (CH), 129.8 (CH), 133.2 (C), 138.1 (C), 168.2 (C), 203.2 (C).

Methyl 4-methyl-2-[1-(4-methylphenyl)-2-nitroethyl]-3-oxobutanoate (4e)

Pale-yellow oil (*dr* = 53/47). Major isomer ^{1}H NMR (400 MHz, $CDCl_3$) δ 0.70 (d, *J* = 6.8 Hz, 3H), 0.97 (d, *J* = 7.2 Hz, 3H), 2.27 (s, 3H), 2.43 (qq, *J* = 7.2, 6.8 Hz, 1H), 3.72 (s, 3H), 4.13 (d, *J* = 9.6 Hz, 1H), 4.22–4.16 (m, 1H), 4.87–4.72 (m, 2H), 7.07 (d, *J* = 8.0 Hz, 2H), 7.10 (d, *J* = 8.0 Hz, 2H); ^{13}C NMR (100 MHz, $CDCl_3$) δ 17.4 (CH_3), 17.7 (CH_3), 21.0 (CH_3), 41.9 (CH), 42.7 (CH), 52.8 (CH_3), 77.7 (CH_2), 127.9 (CH), 129.7 (CH), 133.4 (C), 138.0 (C), 168.0 (C), 206.4 (C); HRMS (ESI–TOF) calcd. for [$M+Na^+$] $C_{16}H_{21}NO_5$

330.1312, found 330.1324. Minor isomer ^{1}H NMR (400 MHz, $CDCl_3$) δ 1.05 (d, *J* = 7.2 Hz, 3H), 1.06 (d, *J* = 6.8 Hz, 3H), 2.28 (s, 3H), 2.70 (qq, *J* = 6.8, 7.2 Hz 1H), 3.51 (s, 3H), 4.22–4.16 (m, 1H), 4.24 (d, *J* = 8.8 Hz, 1H), 4.87–4.72 (m, 2H), 7.07 (d, *J* = 8.0 Hz, 2H), 7.10 (d, *J* = 8.0 Hz, 2H); ^{13}C NMR (100 MHz, $CDCl_3$) δ 17.7 (CH_3), 18.1 (CH_3), 21.0 (CH_3), 41.4 (CH), 42.3 (CH), 52.6 (CH_3), 59.5 (CH), 77.5 (CH_2), 127.7 (CH), 129.6 (CH), 133.4 (C), 138.0 (C), 167.6 (C), 207.5 (C).

3-[1-(4-Methylphenyl)-2-nitroethyl]-1-phenyl-1,3-butanedione (4g)

Yellow oil (*dr* = 53/47). Major isomer ^{1}H NMR (400 MHz, $CDCl_3$) δ 1.93 (s, 3H), 2.29 (s, 3H), 4.49 (ddd, *J* = 4.8, 8.4, 10.0 Hz, 1H), 4.63 (dd, *J* = 8.4, 12.4 Hz, 1H), 4.69 (dd, *J* = 4.8, 12.4 Hz, 1H), 5.16 (d, *J* = 10.0 Hz, 1H), 7.15 (d, *J* = 8.2 Hz, 2H), 7.14 (d, *J* = 8.2 Hz, 2H), 7.49 (dd, *J* = 7.4, *J* = 7.4 Hz, 2H), 7.62 (t, *J* = 7.4 Hz, 1H), 8.03 (dd, *J* = 7.4, 1.2 Hz, 2H); ^{13}C NMR (100 MHz, $CDCl_3$) δ 21.1 (CH_3), 28.4 (CH), 65.5 (CH), 78.4 (CH_2), 128.0 (CH), 129.0 (CH), 129.1 (CH), 129.9 (CH), 132.8 (C), 134.4 (CH), 136.2 (C), 138.3 (C), 194.1 (C), 200.9 (C); HRMS (ESI-TOF) calcd. for $C_{19}H_{19}NO_4$ 348.1206, found 348.1201. Minor isomer ^{1}H NMR (400 MHz, $CDCl_3$) δ 2.20 (s, 3H), 2.21 (s, 3H), 4.40 (ddd, *J* = 4.4, 8.6, 9.6 Hz, 1H), 4.73 (dd, *J* = 4.4, 12.6 Hz, 1H), 4.82 (dd, *J* = 8.6, 12.6 Hz, 1H), 5.18 (d, *J* = 9.6 Hz, 1H), 6.98 (d, *J* = 8.2 Hz, 2H), 7.05 (d, *J* = 8.2 Hz, 2H), 7.42 (dd, *J* = 7.4, 7.4 Hz, 2H), 7.56 (t, *J* = 7.4 Hz, 1H), 7.82 (dd, *J* = 7.4, 1.2 Hz, 2H); ^{13}C NMR (100 MHz, $CDCl_3$) δ 20.9 (CH_3), 29.7 (CH), 43.1 (CH_3), 64.9 (CH), 78.2 (CH_2), 127.8 (CH), 128.6 (CH), 128.9 (CH), 129.6 (CH), 133.5 (C), 134.0 (CH), 136.4 (C), 137.8 (C), 193.8 (C), 201.8 (C).

3-[1-(4-Chlorophenyl)-2-nitroethyl]-2,4-pentanedione [3]

Colorless solid. ^{1}H NMR (400 MHz, $CDCl_3$) δ 1.97 (s, 3H), 2.29 (s, 3H), 4.25–4.20 (m, 1H), 4.32 (d, J = 10.7 Hz, 1H), 4.16–4.60 (m, 2H), 7.13 (d, J = 8.5 Hz, 2H), 7.31 (d, J = 8.5 Hz, 2H); ^{13}C NMR (100 MHz, $CDCl_3$) δ 29.6 (CH_3), 30.4 (CH_3), 42.2 (CH), 70.6 (CH), 77.9 (CH_2), 129.3 (C), 129.6 (CH), 134.6 (C), 200.5 (C), 201.4 (C).

Spectral data of 1

Ethyl 2-(4-methylphenyl)-1-ethanoyl-3-nitrocyclopropane-1-carboxylate (1c)

Yellow oil.^{1}H NMR (400 MHz, $CDCl_3$) δ 0.98 (t, J = 7.2 Hz, 3H), 2.31 (s, 3H), 2.39 (s, 3H), 3.99 (q, J = 7.2 Hz, 2H), 4.16 (d, J = 6.0 Hz, 1H), 5.41 (d, J = 6.0 Hz, 1H), 7.11 (s, 4H); ^{13}C NMR (100 MHz, $CDCl_3$) δ 13.8 (CH_3), 21.2 (CH_3), 29.3 (CH_3), 36.9 (CH_3), 51.5 (C), 62.7 (CH_2), 67.5 (CH), 127.4 (C), 128.1 (CH), 129.5 (CH), 138.4 (C), 164.0 (C), 194.6 (C); HRMS (APCI/TOF): *m/z* calcd. for $C_{15}H_{17}NO_5$ $[M - H]^-$ 290.1034, found 290.1060.

Methyl 2-(4-methylphenyl)-3-nitro-1-propanoylcyclopropane-1-carboxylate (1d)

Pale yellow oil. ^{1}H NMR (400 MHz, $CDCl_3$) δ 1.15 (t, J = 7.2 Hz, 3H), 2.32 (s, 3H), 2.54 (dq, J = 18.2, 7.2 Hz, 1H), 2.82 (dq, J = 18.2, 7.2 Hz, 1H), 3.54 (s, 3H), 4.17 (d, J = 6.0 Hz, 1H), 5.41 (d, J = 6.0 Hz, 1H), 7.12 (s, 4H); ^{13}C NMR (100 MHz, $CDCl_3$) δ 7.92 (CH_3), 21.2 (CH_3), 35.5 (CH_2), 36.8 (CH), 51.3 (C), 53.3 (CH_3), 67.3 (CH), 127.5 (C), 128.1 (CH), 129.6 (CH), 138.5 (C), 164.7 (C), 197.7 (C); HRMS (APCI/TOF): *m/z* calcd. for $C_{15}H_{17}NO_5$ $[M - H]^-$ 290.1034, found 290.1012.

Methyl 2-(4-methylphenyl)-1-(2-methylpropanoyl)-3-nitrocyclopropane-1-carboxylate (1e)

Pale-yellow oil (*dr* = 83/17). Major isomer ^{1}H NMR (400 MHz, $CDCl_3$) δ 1.12 (d, J = 6.8 Hz, 3H), 1.19 (d, J = 6.8 Hz, 3H), 2.32 (s, 3 H), 2.97 (qq, J = 6.8, 6.8 Hz, 1H), 3.51 (s, 3 H), 4.23 (d, J = 6.0 Hz, 1H), 5.45 (d, J = 6.0 Hz, 1H), 7.13–7.09 (m, 4 H); ^{13}C NMR (100

MHz, $CDCl_3$) δ 18.1 (CH_3), 18.2 (CH_3), 21.1 (CH), 36.6 (CH_3), 40.4 (CH), 51.3 (C), 53.1 (OCH_3), 67.6 (CH), 127.5 (C), 128.0 (CH), 129.5 (CH), 138.4 (C), 164.7 (C), 200.4 (C); IR (ATR) 1361, 1557, 1715, 1730 cm^{-1}; HRMS (ESI-TOF) calcd. for $C_{16}H_{19}NO_5Na$ [$M+Na^+$] 328.1155, found 328.1159.

1-Benzoyl-1-ethanoyl-2-(4-methylphenyl)-3-nitrocyclopropane (1g)

Yellow oil. ^1H NMR (400 MHz, $CDCl_3$) δ 1.87 (s, 3H), 2.33 (s, 3H), 4.30 (d, *J* = 5.6 Hz, 1H), 5.83 (d, *J* = 5.6 Hz, 1H), 7.16 (s, 4H), 7.50 (dd, *J* = 7.2, 7.2 Hz, 2H), 7.63 (t, *J* = 7.2 Hz, 1H), 7.95 (d, *J* = 7.2 Hz, 2H); ^{13}C NMR (100 MHz, $CDCl_3$) δ 21.2 (CH_3), 29.8 CH_3), 38.2 (CH), 56.8 (C), 66.7 (CH), 126.8 (C), 128.1 (CH), 128.7 (CH), 129.5 (CH), 129.7 (CH), 135.0 (CH), 138.5 (C), 138.6 (C), 195.9 (C), 210.1 (C); HRMS (APCI/TOF): *m/z* calcd. for $C_{19}H_{17}NO_4$ $[M + H]^+$ 324.1230, found 324.1254. In spite of several attempts to separate **1g** and by-products, it could not be isolated completely.

Spectral data of 8

3-Ethanoyl-4,5-dihydro-2-methyl-4-(4-methylphenyl)-5-nitrofuran (8b) [4]

Yellow oil. ^1H NMR (400 MHz, $CDCl_3$) δ 2.02 (s, 3H), 2.35 (s, 3H), 2.53 (s, 3H), 4.61 (br s, 1H), 5.71 (br s, 1H), 7.12 (d, *J* = 8.0, 2H), 7.20 (d, *J* = 8.0, 2H); ^{13}C NMR (100 MHz, $CDCl_3$) δ 193.2 (C), 166.8 (C), 138.6 (C), 134.5 (C), 130.2 (CH), 127.0 (CH), 115.6 (C), 109.7 (CH), 56.0 (CH), 29.7 (CH_3), 21.1 (CH_3), 14.5 (CH_3).

Ethyl 4,5-dihydro-2-methyl-4-(4-methylphenyl)-5-nitrofuran-3-carboxylate (8c) [5]

Pale-yellow oil. ^1H NMR (400 MHz, $CDCl_3$) δ 1.10 (t, *J* = 7.1 Hz, 3H), 2.34 (s, 3H), 2.49 (s, 3H), 4.06 (q, *J* = 7.1 Hz, 3H), 4.56 (br s, 1H), 5.76 (d, *J* = 1.5 Hz, 1H), 7.10 (d, *J* = 8.0 Hz, 2H), 7.17 (d, *J* = 8.0 Hz, 2H); ^{13}C NMR (100 MHz, $CDCl_3$) δ 13.8 (CH_3), 14.0 (CH_3), 21.1 (CH_3), 55.5 (CH), 60.3 (CH_2), 107.9 (C), 109.8 (CH), 126.9 (CH), 129.8 (CH), 135.0 (C), 163.5 (C), 166.8 (C).

Methyl 2-ethyl-4,5-dihydro-4-(4-methylphenyl)-5-nitrofuran-3-carboxylate (8d)

Pale-yellow oil. ^{1}H NMR (400 MHz, $CDCl_3$) δ 1.34 (dd, *J* = 7.6, 7.6 Hz, 3H), 2.34 (s, 3H), 2.84 (dq, *J* = 7.6, 14.8 Hz, 1H), 3.02 (dq, *J* = 7.6, 14.8 Hz, 1H), 3.60 (s, 3H), 4.56 (br s, 1H), 5.73 (br s, 1H), 7.10 (d, *J* = 7.8 Hz, 2H), 7.17 (d, *J* = 7.8 Hz, 2H); ^{13}C NMR (100 MHz, $CDCl_3$) δ 10.9 (CH_3), 21.0 (CH_2), 21.0 (CH_2), 51.4 (CH_3), 55.4 (CH), 106.6 (C), 109.7 (CH), 126.8 (CH), 129.9 (CH), 134.9 (C), 138.1 (C), 163.8 (C), 171.9 (C); IR (ATR) 1368, 1566, 1715 cm^{-1}; HRMS (ESI-TOF) calcd. for $C_{15}H_{17}NO_5$ [M+H$^+$] 292.1180, found 292.1179.

Methyl 4,5-dihydro-2-(1-methylethyl)-4-(4-methylphenyl)-5-nitrofuran-3-carboxylate (8e)

Pale-yellow oil. ^{1}H NMR (400 MHz, $CDCl_3$) δ 1.32 (d, *J* = 6.9 Hz, 3H), 1.35 (d, *J* = 6.9 Hz, 3H), 2.35 (s, 3H), 3.60 (s, 3H), 3.80 (qq, *J* = 6.9, 6.9 Hz, 1H), 4.55 (d, *J* = 1.5 Hz, 1H), 5.73 (d, *J* = 1.5 Hz, 1H), 7.09 (d, *J* = 8.0 Hz, 2H), 7.17 (d, *J* = 8.0 Hz, 2H); ^{13}C NMR (100 MHz, $CDCl_3$) δ 19.2 (CH_3), 19.5 (CH_3), 21.1 (CH), 26.9 (CH_3), 51.4 (CH_3), 55.3 (CH), 105.5 (C), 109.6 (CH), 126.8 (CH), 129.9 (CH), 135.0 (C), 138.1 (C), 163.8 (C), 174.8 (C); HRMS (ESI-TOF) calcd. for $C_{16}H_{19}NO_5$ [M+H$^+$] 306.1336, found: 306.1336.

3-Benzoyl-4,5-dihydro-2-methyl-4-(4-methylphenyl)-5-nitrofuran (8g)

Yellow oil. ^{1}H NMR (400 MHz, $CDCl_3$) δ 1.98 (s, 3H), 2.36 (s, 3H), 4.80 (d, *J* = 1.6 Hz, 1H), 5.83 (d, *J* = 1.6 Hz, 1H), 7.23–7.22 (m, 4H), 7.61–7.51 (m, 3H), 7.90–7.87 (m, 2H); ^{13}C NMR (100 MHz, $CDCl_3$) δ 21.1 (CH_3), 29.5 (CH_3), 57.1 (CH), 109.0 (CH), 117.4 (C), 127.0 (CH), 128.3 (C), 128.6 (CH), 129.9 (CH), 130.1 (CH), 131.9 (CH), 134.6 (C), 138.5 (C), 164.1 (C), 192.5 (C); IR (ATR) 1361, 1558, 1645 cm^{-1}; HRMS (ESI-TOF) calcd. for $C_{19}H_{19}NO_6$ [M+Na$^+$] 346.1050, found: 346.1043.

3-Ethanoyl-4,5-dihydro-4-(4-methylphenyl)-2-phenyl-5-nitrofuran (8g')

Yellow oil. ^{1}H NMR (400 MHz, $CDCl_3$) δ 2.12 (s, 3H), 2.32 (s, 3H), 4.84 (br s, 1H), 5.85 (d, *J* = 2.0 Hz, 1H), 7.16–7.11 (m, 4H), 7.43–7.39 (m, 2H), 7.55–7.49 (m, 3H); ^{13}C NMR (100 MHz, $CDCl_3$) δ 14.9 (CH_3), 21.1 (CH_3), 56.8 (CH), 110.0 (CH), 116.6 (C), 126.9 (CH), 128.1 (CH), 128.5 (CH), 129.9 (CH), 132.2 (CH), 134.7 (C), 138.2 (C), 139.3 (C), 165.0 (C), 191.0 (C); IR (ATR)1354, 1568, 1633 cm^{-1}; HRMS (ESI-TOF) calcd. for $C_{16}H_{19}NO_7$ [M+Na^{+}] 346.1050, found:346.1054.

Methyl 5-isopropyl-3-(4-methylphenyl)-2-nitro-4-carboxylate (11e)

Yellow oil. ^{1}H NMR (400 MHz, $CDCl_3$) δ 1.40 (d, *J* = 7.0 Hz, 6H), 2.41 (s, 3H), 3.66 (s, 3H), 3.77 (septet, *J* = 7.0 Hz, 1H), 7.26-7.20 (m, 4H); ^{13}C NMR (100 MHz, $CDCl_3$) δ 20.5 (CH_3), 21.5 (CH), 28.0 (CH_3), 51.8 (CH_3), 115.5 (C), 125.8 (C), 127.8 (C), 128.6 (CH), 129.2 (CH), 138.9 (C), 162.4 (C), 167.4 (C); IR (ATR/cm^{-1}): 1715, 1518, 1360; HRMS (ESI/TOF): *m/z* calcd. for $C_{16}H_{17}NO_5$ [M + H]$^{+}$ 304.1180, found 304.1174.

Information about X-ray analysis and spectral data of 10

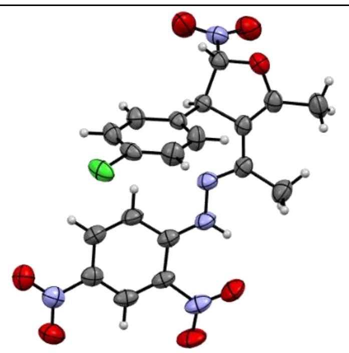

10

**Empirical formula**	$C_{19}H_{16}ClN_5O_7$
**Formula weight**	461.82
**Temperature (K)**	298(2)
**Crystal system**	monoclinic
**Space group**	*P*21/*c* (#14)
**Unit cell Dimensions**	a = 14.972(5) Å, b = 8.905(5) Å<br>c = 15.738(3) Å, β = 95.65(2)°
**Volume (Å)**	2088.1(6)
**Z**	4
**ρ_{calc} (g•cm^{-3})**	1.469
**Absorption coefficient (mm^{-1})**	0.236
**θ range (°)**	2.601 to 27.496
**Reflections collected**	4814
**Independent reflections**	2788
**Completeness to θ**	100
**Goodness-of-fit**	1.026
**Final R indices [$I > 2\sigma(I)$]**	R_1 = 0.0439, wR_2 = 0.1170
**R indices (all data)**	R_1 = 0.1026, wR_2 = 0.1387
**Largest diff. peak (e•Å)**	0.234
**Largest diff. hole (e•Å)**	–0.233
**CCDC number**	2226435

Color labels: gray, carbon; white, hydrogen; light green, chlorine; blue, nitrogen; red, oxygen. The thermal ellipsoids are represented at 50% probability level.

Conjugate addition of acetylacetone 3b to 2-(4-chlorophenyl)-1-nitroethene 2b: To a solution of 2-(4-chlorophenyl)-1-nitroethene (**2b**, 582.6 mg, 3.2 mmol) in dichloromethane (5 mL), were added acetylacetone (**3b**, 1.04 mL, 6 mmol) and triethylamine (84 μL, 0.6 mmol), and the resultant solution was stirred at room temperature for 14 h. After removal of the solvent under reduced pressure, the residual pale-yellow solid was extracted with hot hexane (10 mL × 4). The hexane solution was concentrated, and the residual yellow oil was subjected to column chromatography on silica gel to afford 3-[1-(4-chlorophenyl)-2-nitroethyl]-2,4-pentanedione [3] (eluted with hexane/ethyl acetate 1:1, 236.2 mg, 0.83 mmol, 27%) as a white solid.

3-(4-Chlorophenyl)-4-ethanoyl-2,3-dihydro-5-methyl-2-nitrofuran (9): To a solution of 3-[1-(4-chlorophenyl)-2-nitroethyl]-2,4-pentanedione (236.2 mg, 0.83 mmol) in toluene (3 mL), were added (diacetoxyiodo)benzene (401 mg, 1.25 mmol) and tetrabutylammonium iodide (460 mg, 1.25 mmol), and the resultant mixture was stirred at room temperature for 14 h. The solution was subjected to column chromatography on silica gel to afford 4,5-dihydro-3-acetyl-4-(4-chlorophenyl)-5-nitro-2-methylfuran (**9**, eluted with hexane/ethyl acetate 9:1, 52.1 mg, 0.185 mmol, 23%) as pale yellow oil.

4-Chloro-4,5-dihydro-2-methyl-5-nitro-3-[1-(2,4-dinitrophenylhydrazino)ethyl] furan (10): To a solution of product **9** (83 mg, 0.3 mmol) in ethanol (2 mL), 2,4-dinitrophenylhydrazine (59 mg, 0.3 mmol) and one drop of 12 M hydrochloric acid was added. After heating under reflux for 1 h, the mixture was cooled to 0 °C and hexane (10 mL) was added. The red precipitate was collected by filtration to afford the hydrazone **10** (89 mg, 0.19 mmol, 64%) by filtration. A single crystal suitable for X ray analysis was obtained by further recrystallization from a mixed solvent (dichloromethane/hexane 9:1, 5 mL). Red solid, mp 166–167 °C. ^{1}H NMR (400 MHz, $CDCl_3$) δ 2.21 (s, 3H), 2.52 (s,

3H), 4.80 (br s, 1H), 5.69 (d, J = 2.0, 1H), 7.23 (d, J = 8.5 Hz, 2H), 7.38 (d, J = 8.5 Hz, 2H), 7.40 (d, J = 9.6 Hz, 1H), 8.23 (dd, J = 2.6, 9.6, 1H), 9.08 (d, J = 2.6, 1H), 11.12 (s, 1H); ^{13}C NMR (100 MHz, $CDCl_3$) δ 14.6 (CH_3), 14.8 (CH_3), 57.3 (CH), 108.8 (CH), 114.2 (C), 116.2 (CH), 123.4 (CH), 128.4 (CH), 129.5 (CH), 129.6 (C), 130.0 (CH), 134.4 (C), 136.7 (C), 138.3 (C), 144.4 (C), 146.3 (C), 157.9 (C). HRMS (ESI-TOF) calcd. for $C_{19}H_{16}ClN_5O_7$ [M+H$^+$] 460.0666, found: 460.0685.

Methyl 2-isopropyl-5-(4-methylphenyl)furan-3-carboxylate (**13**): In a screw-capped test tube, a solution of cyclopropane **1e** (152 mg, 0.5 mmol) in benzene (2 mL), was added to a solution of tin(II) chloride dihydrate (225 mg, 1.0 mmol) in benzene (2 mL). After the resulting mixture was heated at 100 °C in a sealed tube for 14 h, the solvent was removed under reduced pressure, and the residue was extracted with diethyl ether (10 mL × 3). The organic layer was evaporated, and the residue was purified by column chromatography on silica gel to afford furan **13** (60 mg, 0.23 mmol, 46%). Colorless oil. ^{1}H NMR (400 MHz, $CDCl_3$) δ 1.34 (d, J = 7.2 Hz, 6H), 2.37 (s, 3H), 3.80 (sept, J = 7.2 Hz, 1H), 3.84 (s, 3H), 6.80 (s, 1H), 7.19 (d, J = 8.0 Hz, 2H), 7.53 (d, J = 8.0 Hz, 2H). ^{13}C NMR (100 MHz, $CDCl_3$) δ 20.9 (CH_3), 21.3 (CH), 27.4 (CH_3), 51.3 (CH_3), 104.6 (CH), 113.1 (C), 123.6 (CH), 127.5 (C), 129.4 (C), 137.5 (C), 151.7 (C), 164.4 (C), 166.4 (C). IR (ATR) = 1718 cm^{-1}; HRMS (ESI-TOF) calcd. for $C_{16}H_{18}O_3$ [M + Na] 281.1148, found 281.1148.

^{1}H and ^{13}C NMR spectra of products

3-[1-(4-Methylphenyl)-2-nitroethyl]-2,4-pentanedione (4b) ($CDCl_3$)

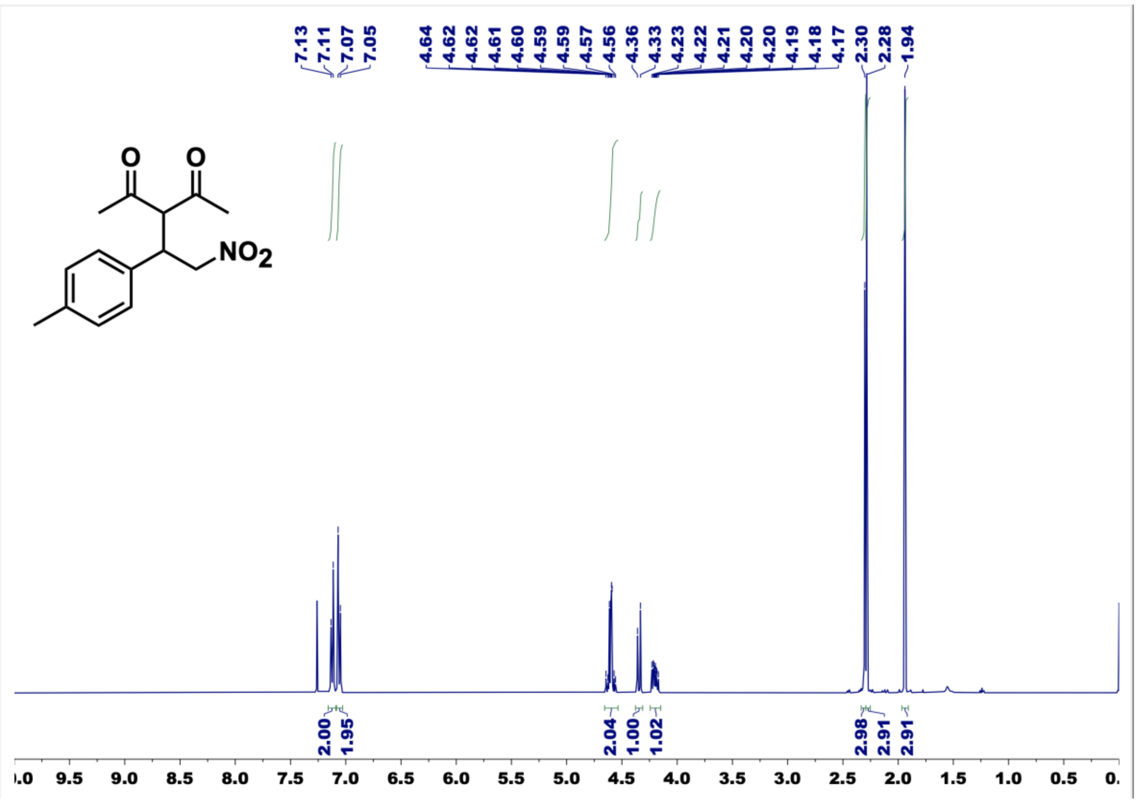

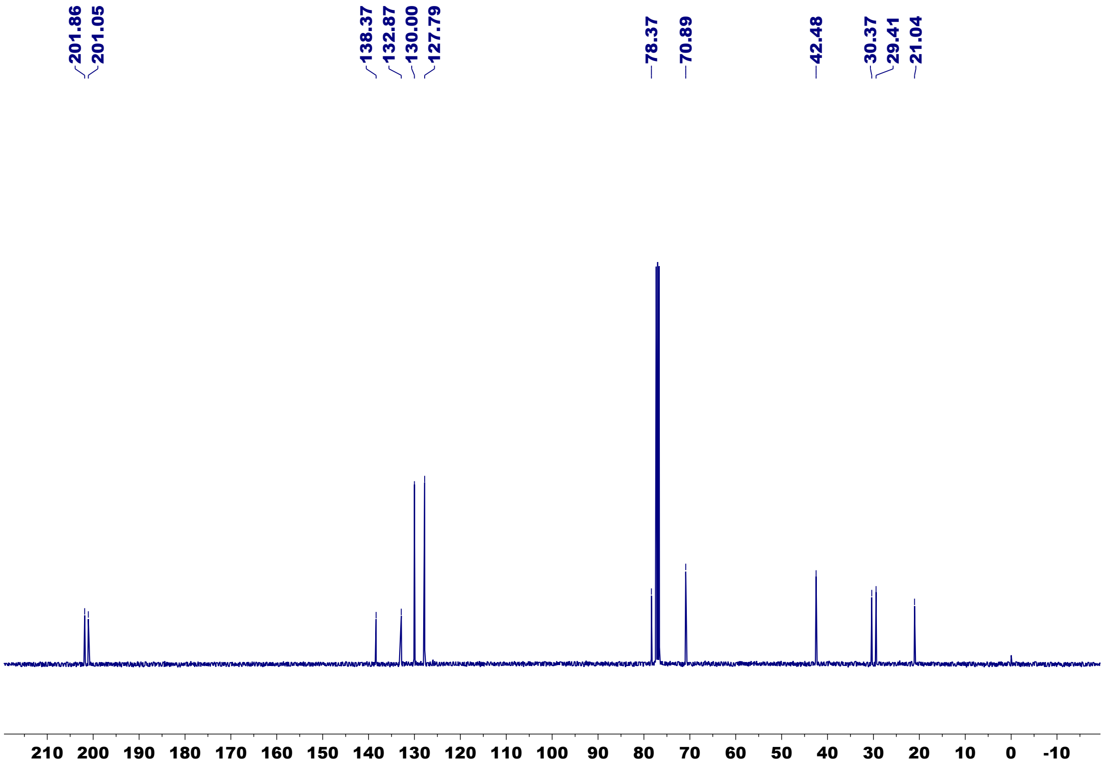

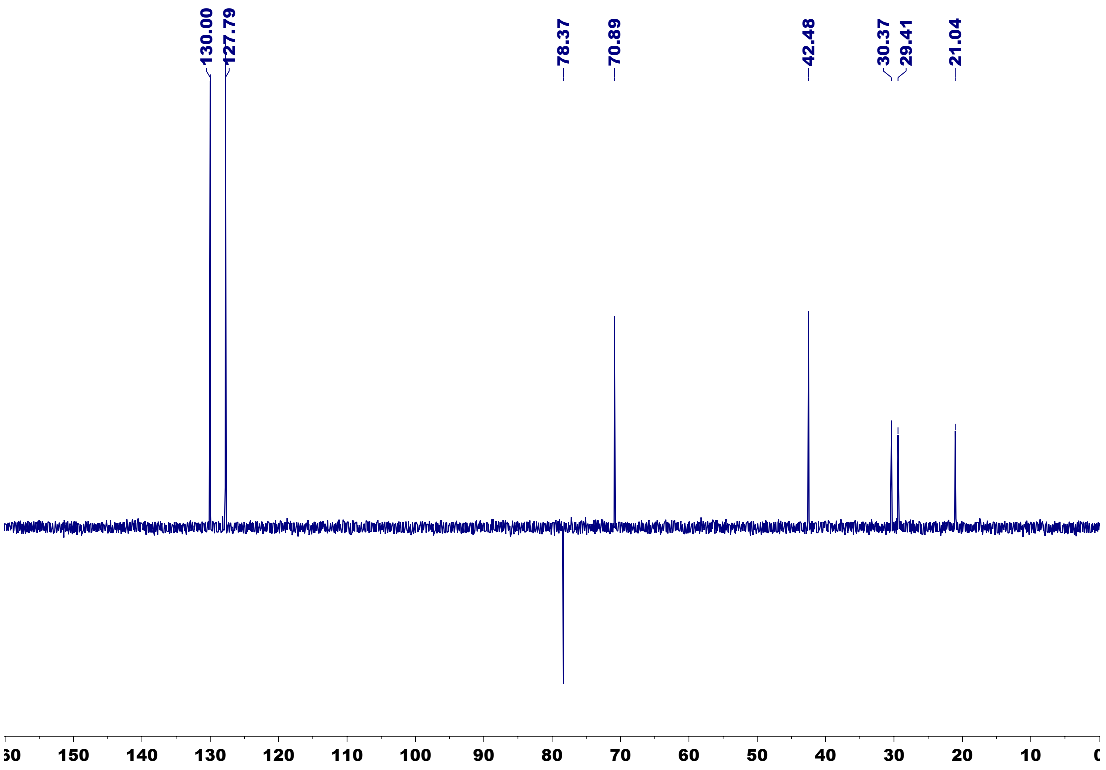

Ethyl 2-[1-(4-methylphenyl)-2-nitroethyl]-3-oxobutanoate (4c) ($CDCl_3$)

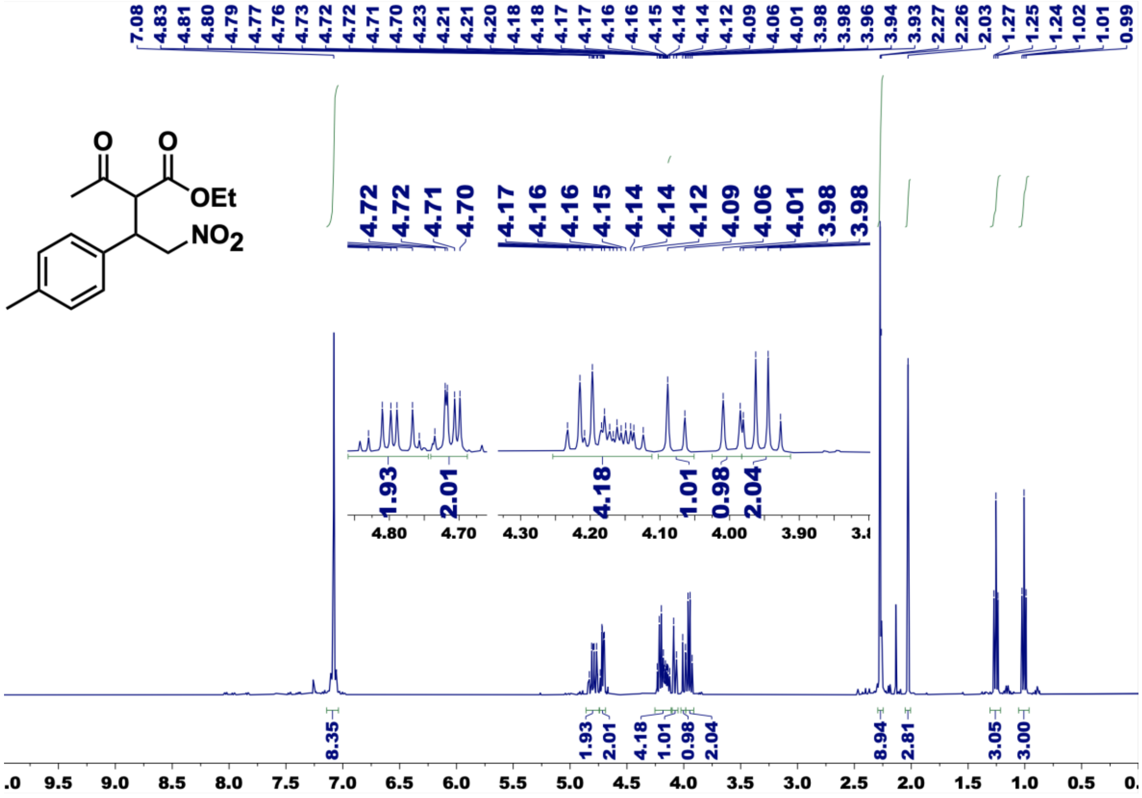

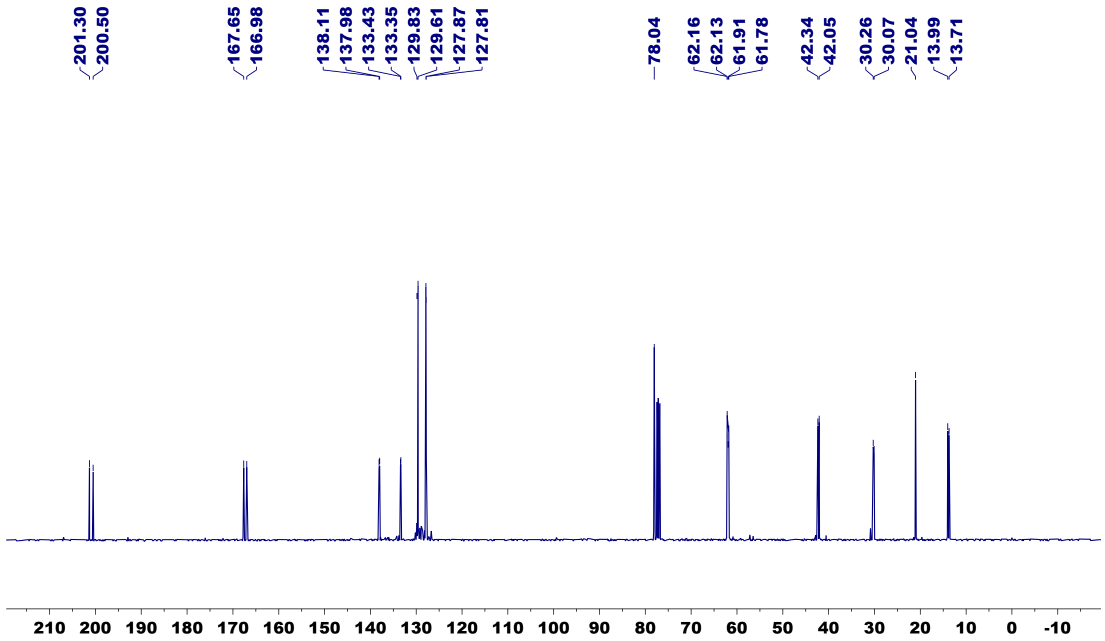
201.30
200.50
167.65
166.98
138.11
137.98
133.43
133.35
129.83
129.61
127.87
127.81
78.04
62.16
62.13
61.91
61.78
42.34
42.05
30.26
30.07
21.04
13.99
13.71
210 200 190 180 170 160 150 140 130 120 110 100 90 80 70 60 50 40 30 20 10 0 -10

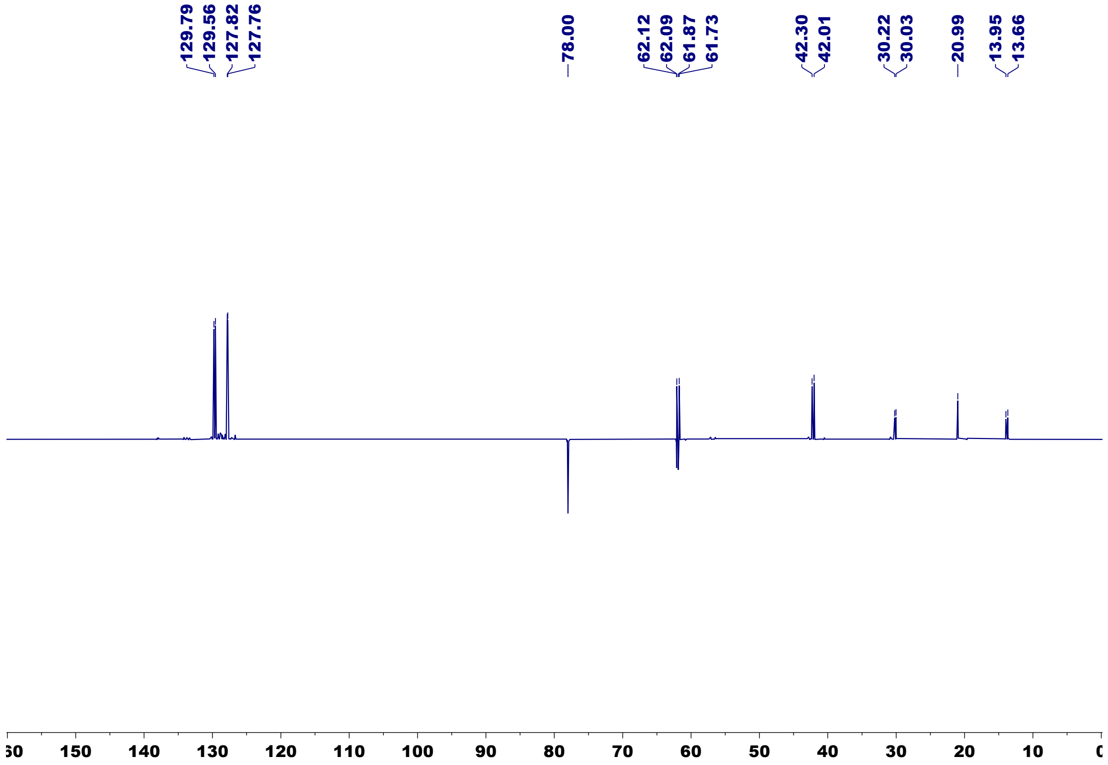
129.79
129.56
127.82
127.76
78.00
62.12
62.09
61.87
61.73
42.30
42.01
30.22
30.03
20.99
13.95
13.66
150 140 130 120 110 100 90 80 70 60 50 40 30 20 10

Methyl 2-[1-(4-methylphenyl)-2-nitroethyl]-3-oxopentanoate (4d) (CDCl$_3$)

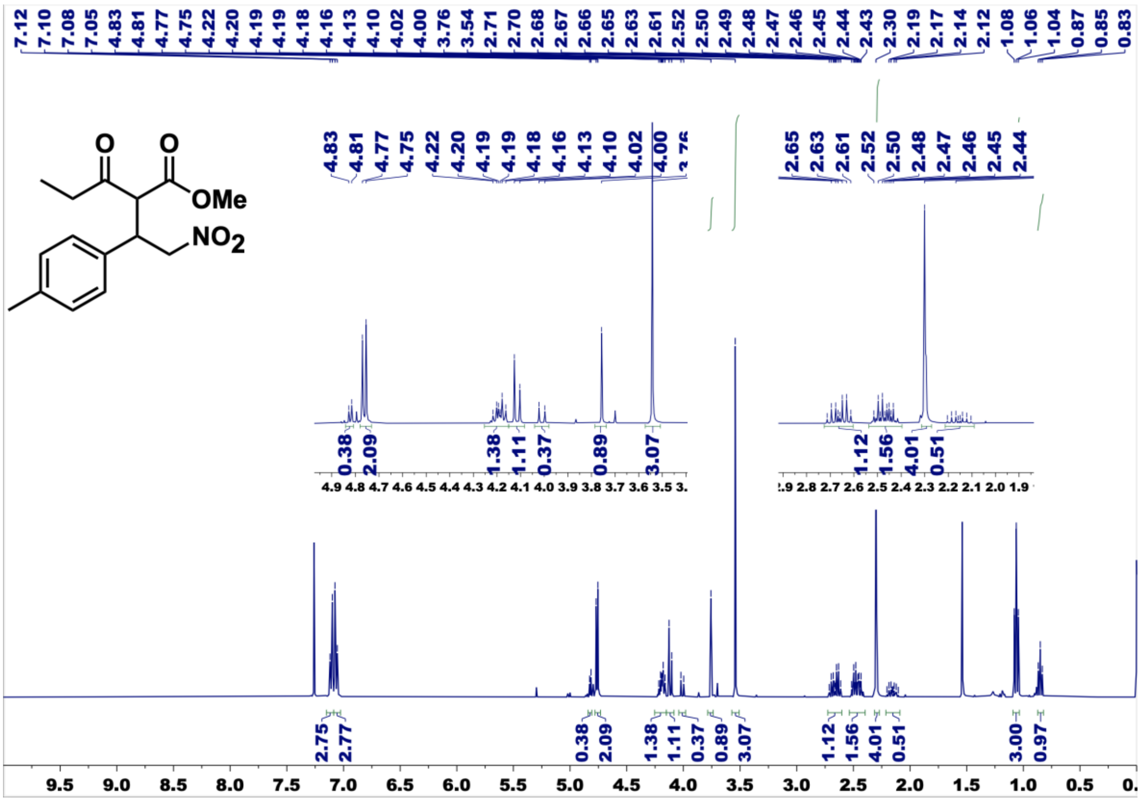

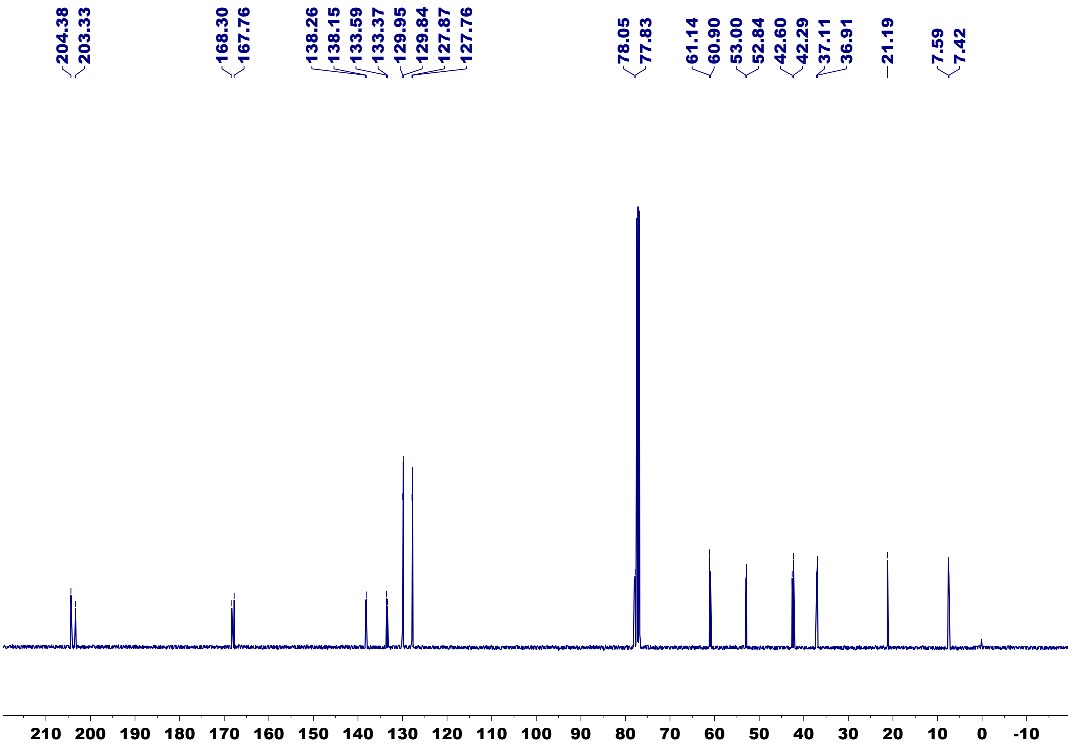

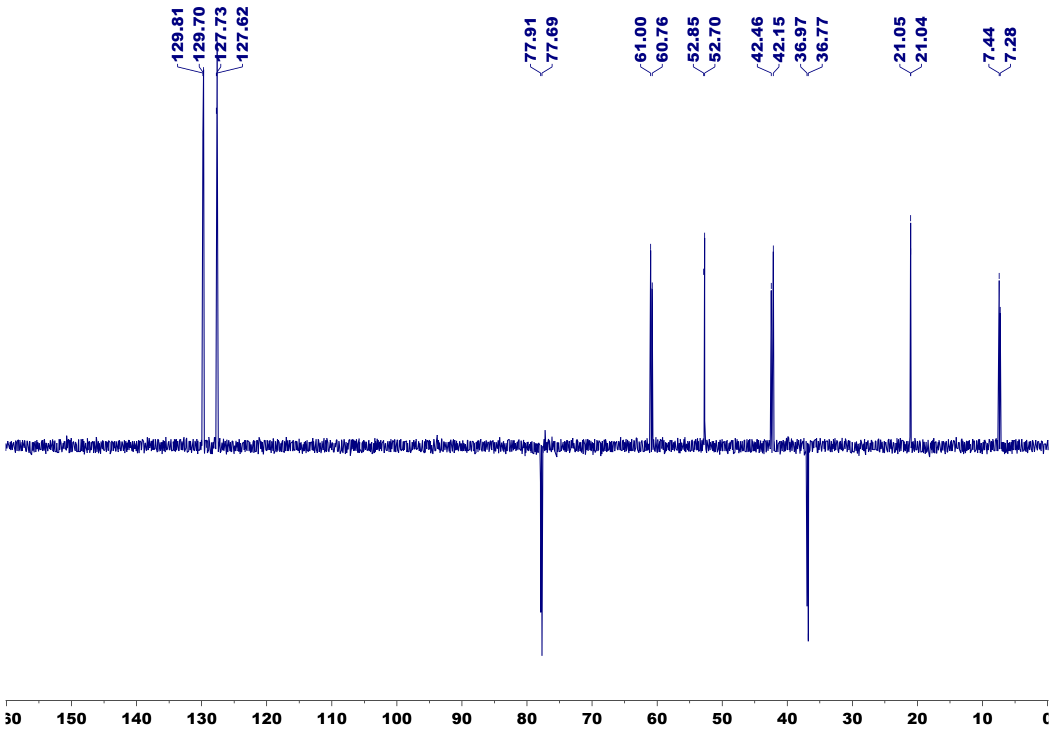

Methyl 4-methyl-2-[1-(4-methylphenyl)-2-nitroethyl]-3-oxobutanoate (4e) ($CDCl_3$)

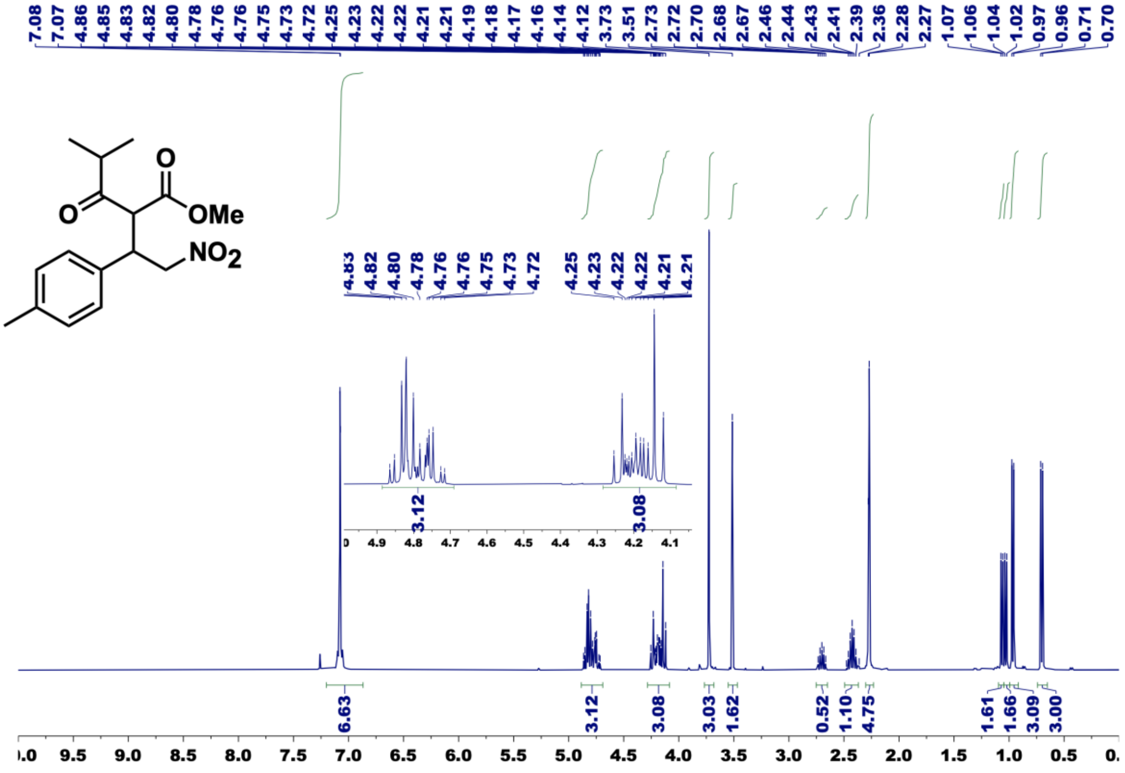

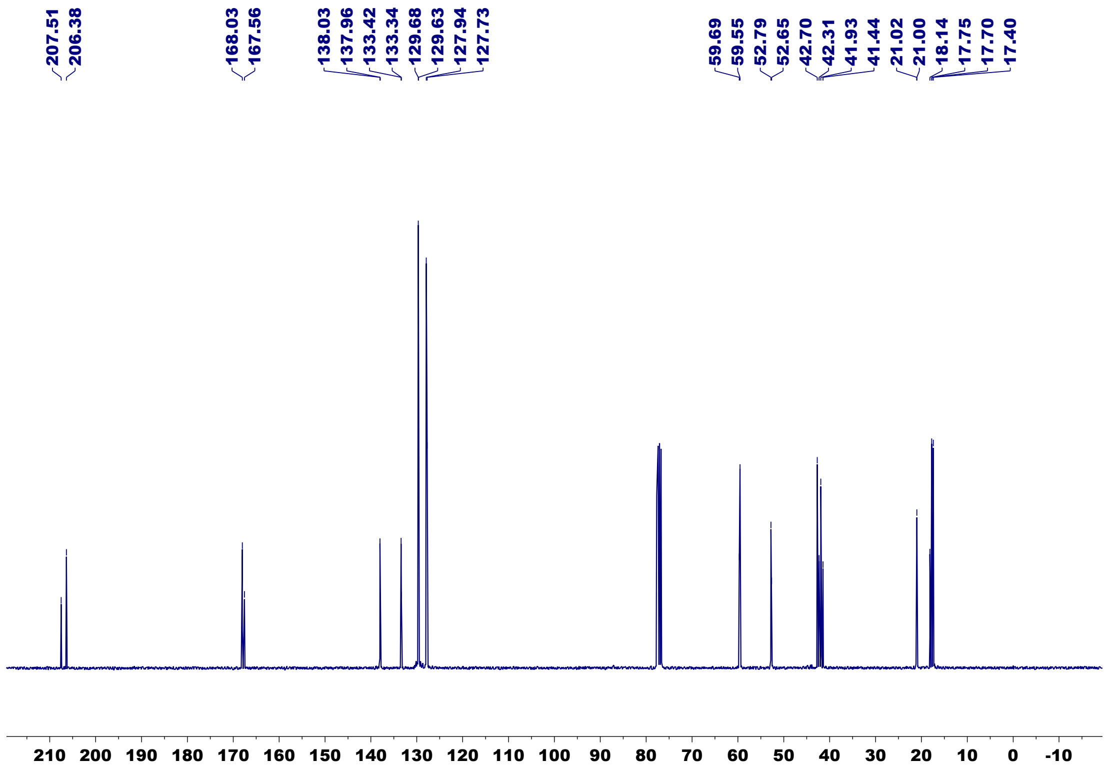
207.51
206.38
168.03
167.56
138.03
137.96
133.42
133.34
129.68
129.63
127.94
127.73
59.69
59.55
52.79
52.65
42.70
42.31
41.93
41.44
21.02
21.00
18.14
17.75
17.70
17.40
210 200 190 180 170 160 150 140 130 120 110 100 90 80 70 60 50 40 30 20 10 0 -10

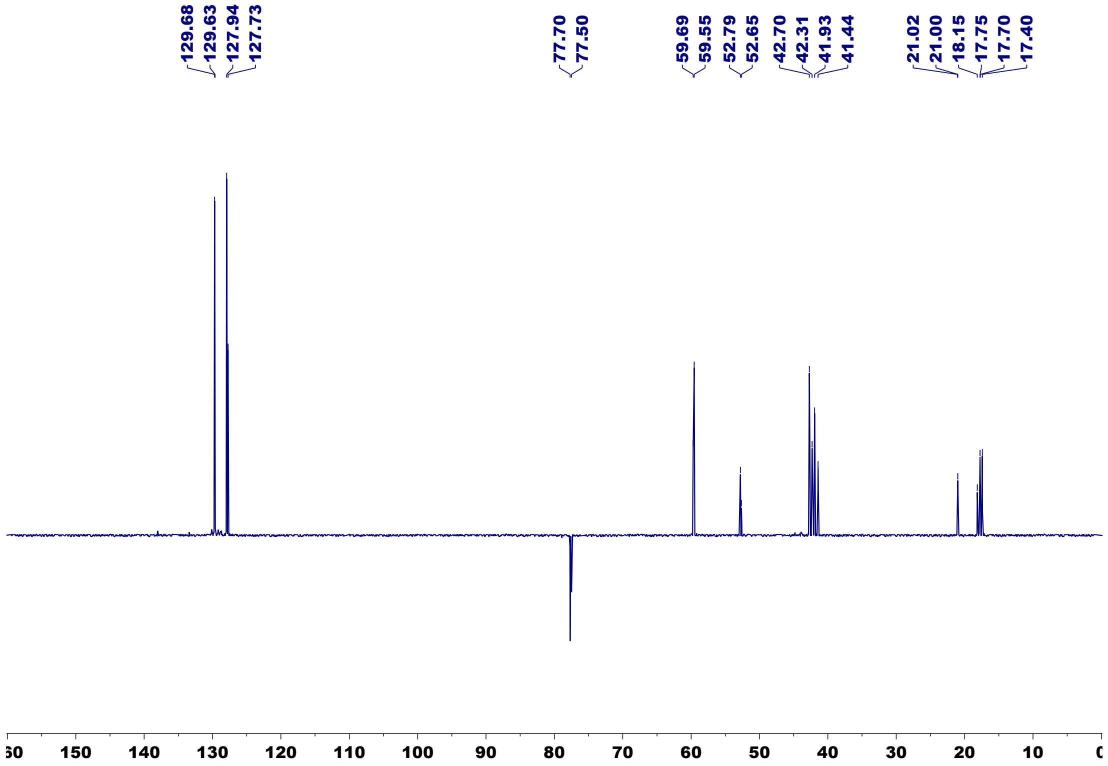
129.68
129.63
127.94
127.73
77.70
77.50
59.69
59.55
52.79
52.65
42.70
42.31
41.93
41.44
21.02
21.00
18.15
17.75
17.70
17.40
150 140 130 120 110 100 90 80 70 60 50 40 30 20 10

Ethyl 2-benzoyl-3-(4-methylphenyl)-4-nitrobutanoate (4f) ($CDCl_3$)

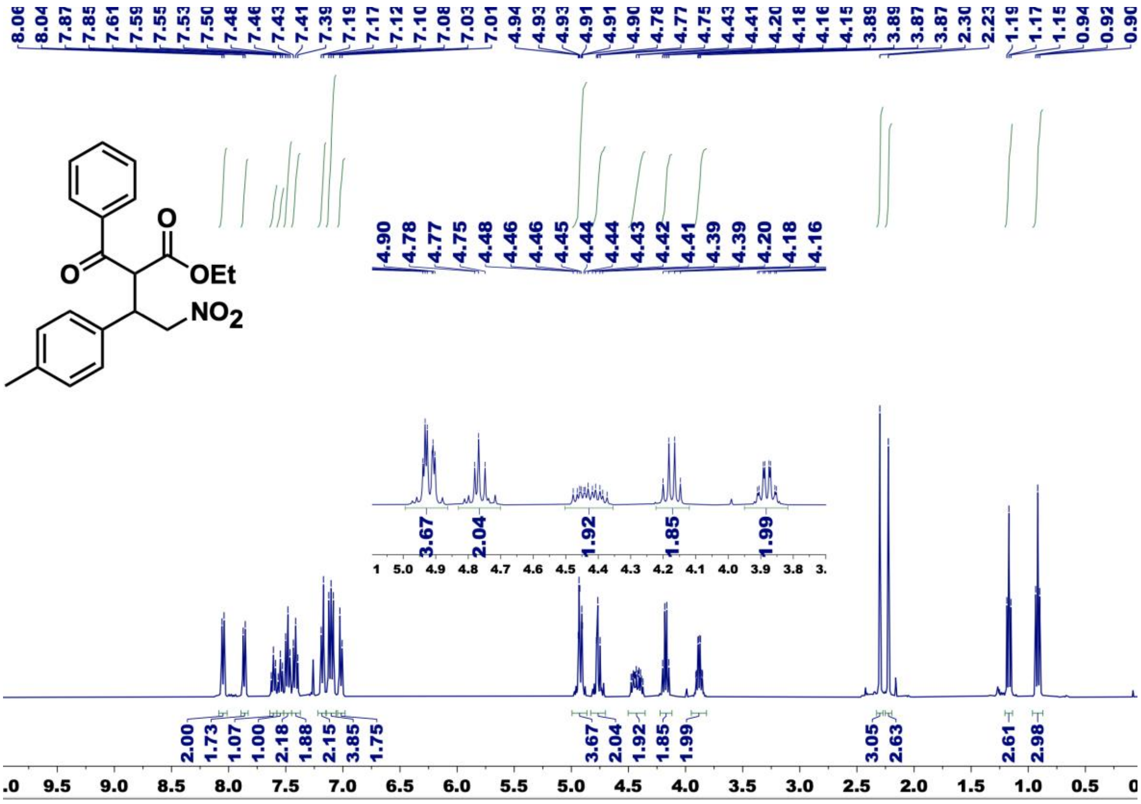

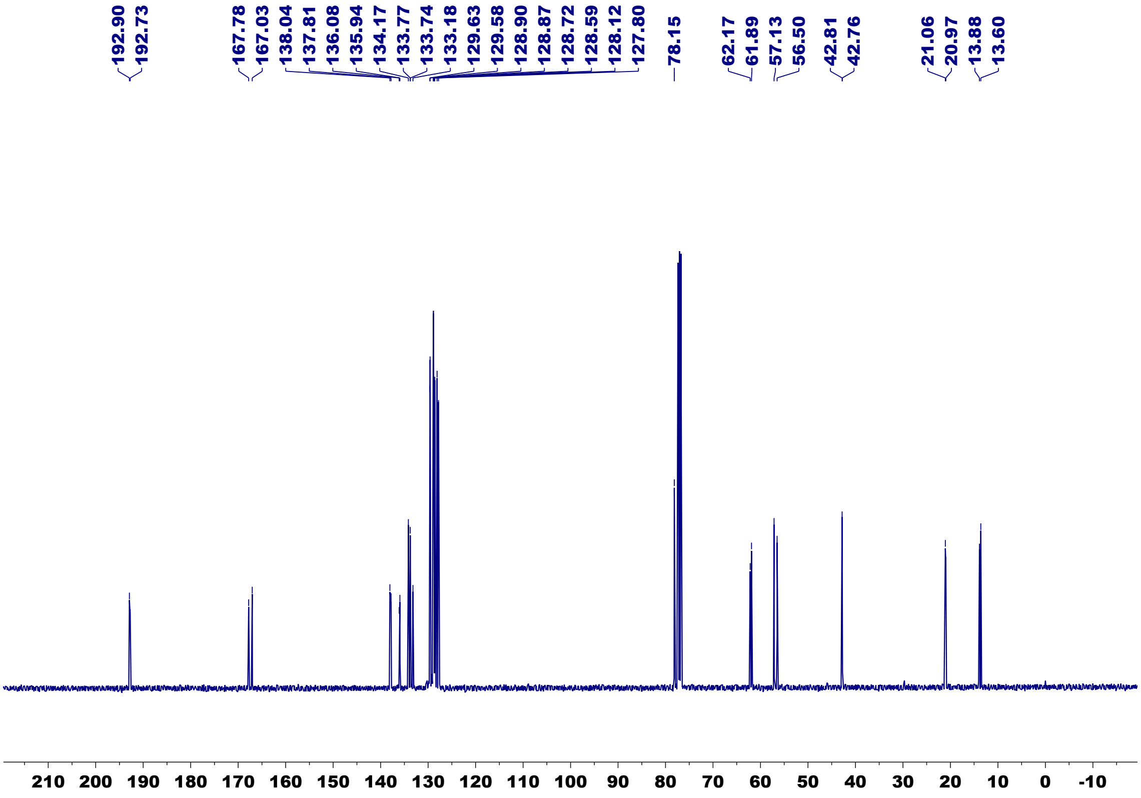

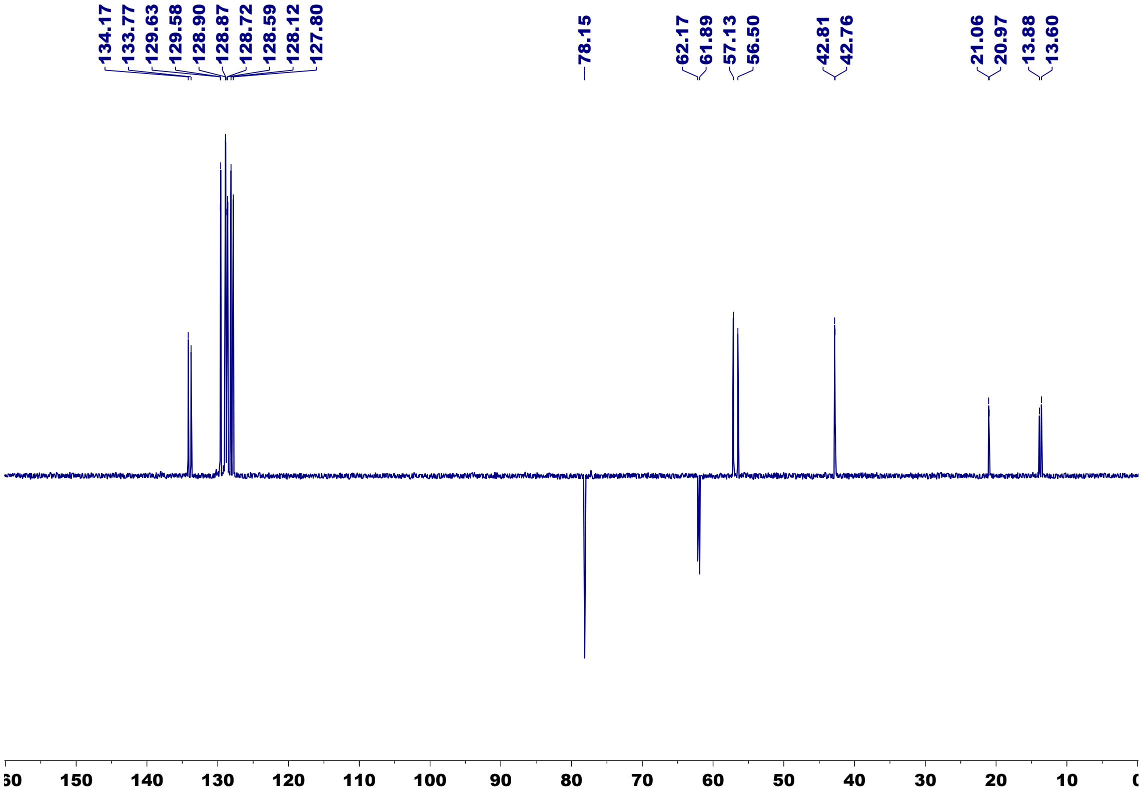

3-[1-(4-Chlorophenyl)-2-nitroethyl]-2,4-pentanedione ($CDCl_3$)

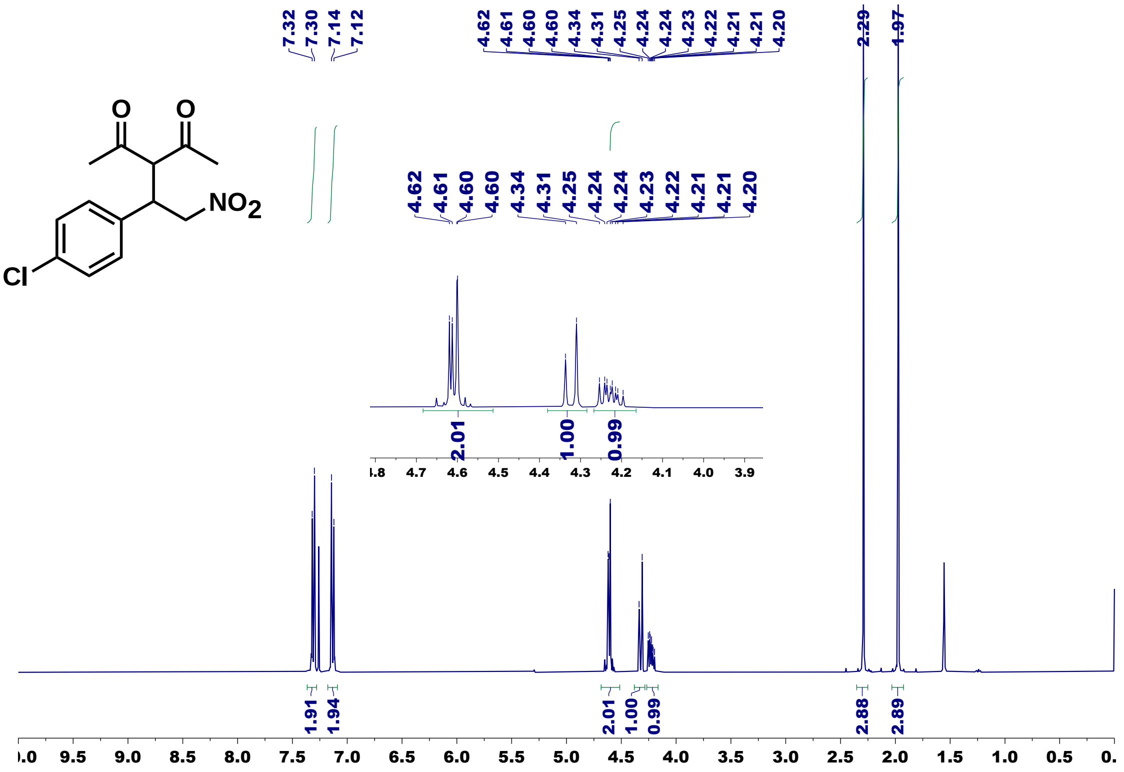

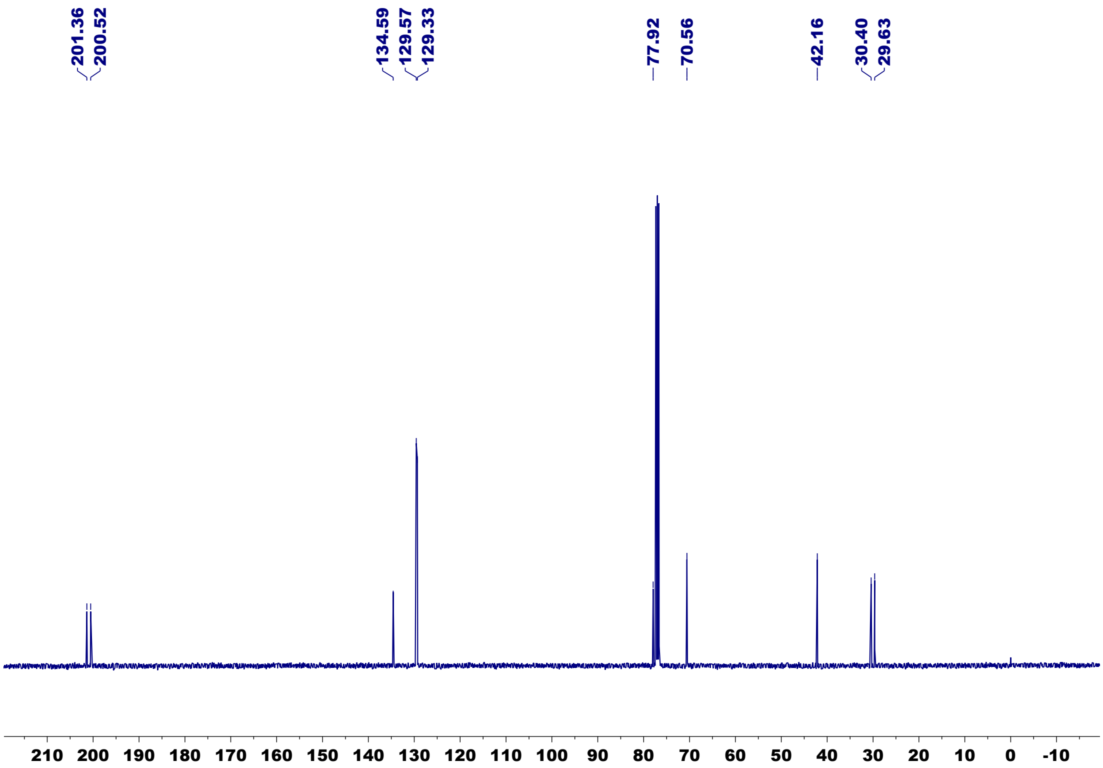
201.36
200.52
134.59
129.57
129.33
77.92
70.56
42.16
30.40
29.63
210 200 190 180 170 160 150 140 130 120 110 100 90 80 70 60 50 40 30 20 10 0 -10

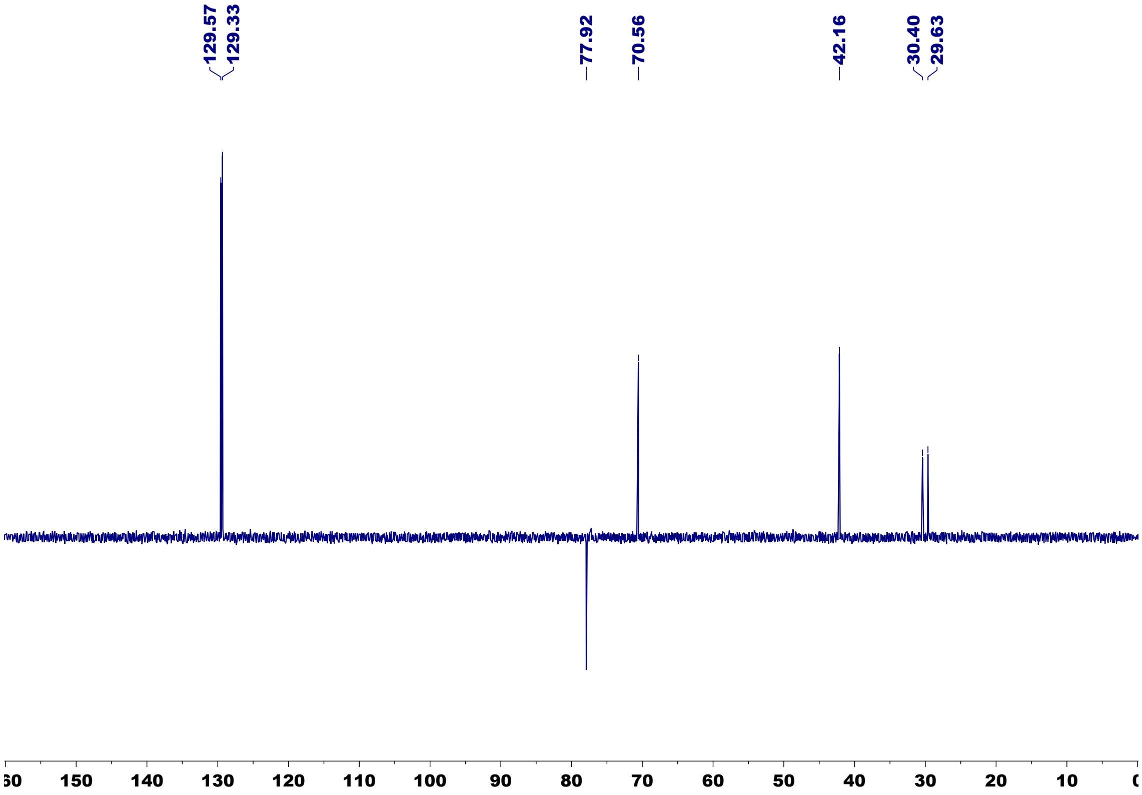
129.57
129.33
77.92
70.56
42.16
30.40
29.63
150 140 130 120 110 100 90 80 70 60 50 40 30 20 10

3-[1-(4-Methylphenyl)-2-nitroethyl]-1-phenyl-1,3-butanedione (4g) ($CDCl_3$)

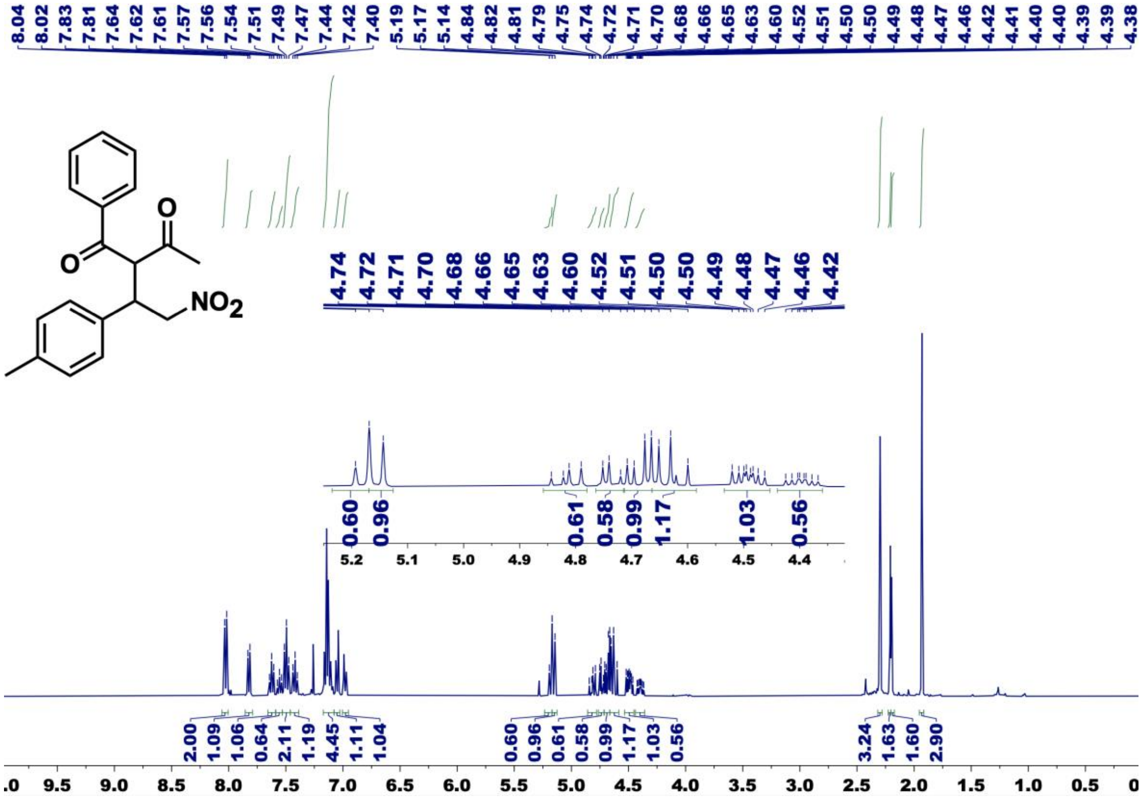

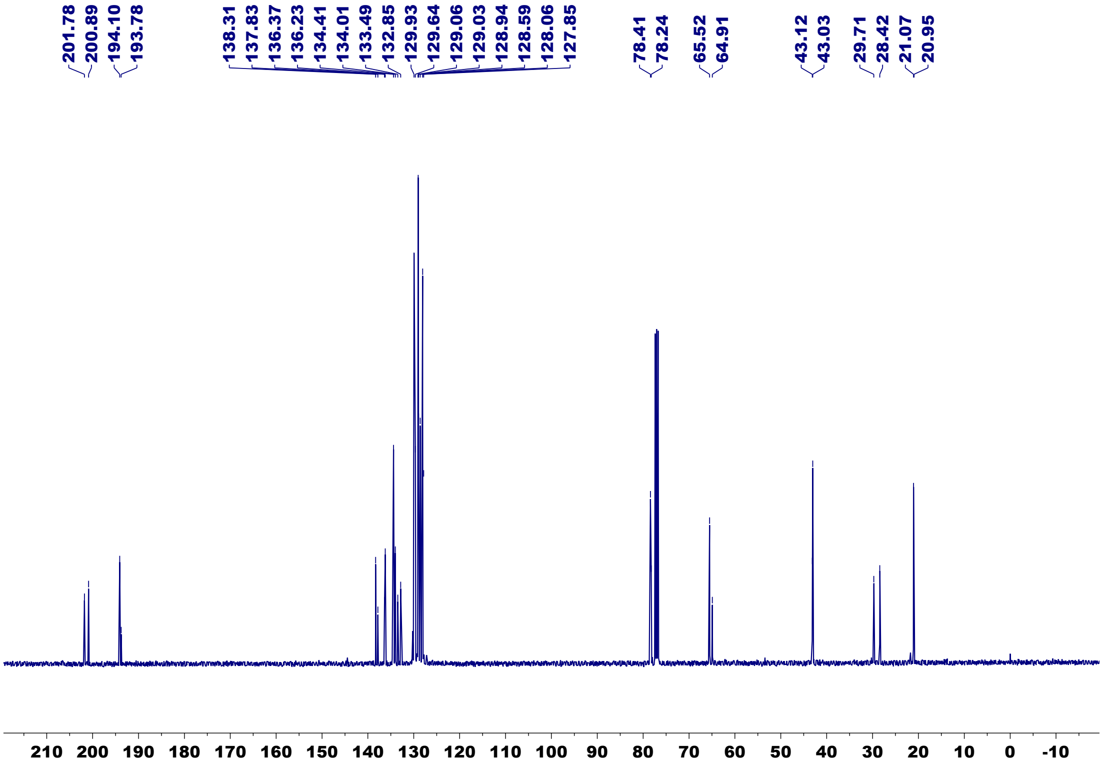

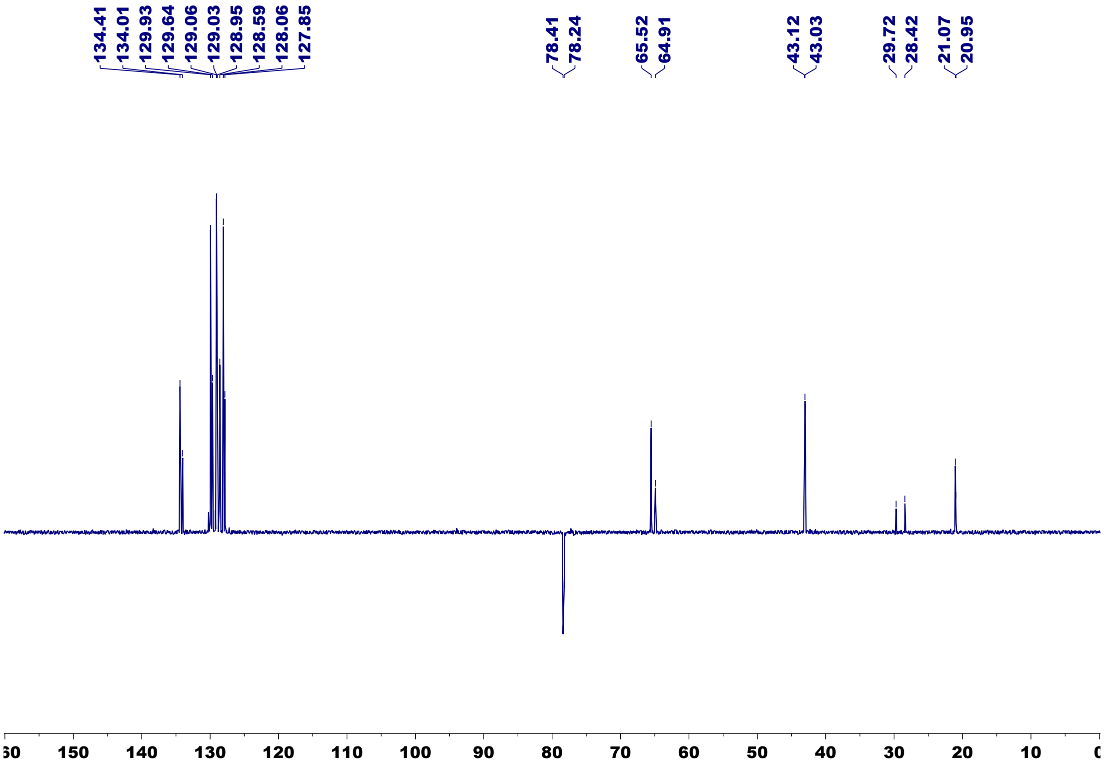

134.41
134.01
129.93
129.64
129.06
129.03
128.95
128.59
128.06
127.85
78.41
78.24
65.52
64.91
43.12
43.03
29.72
28.42
21.07
20.95
150
140
130
120
110
100
90
80
70
60
50
40
30
20
10

Ethyl 1-ethanoyl-2-(4-methylphenyl)-3-nitrocyclopropane-1-carboxylate (1c)
($CDCl_3$)

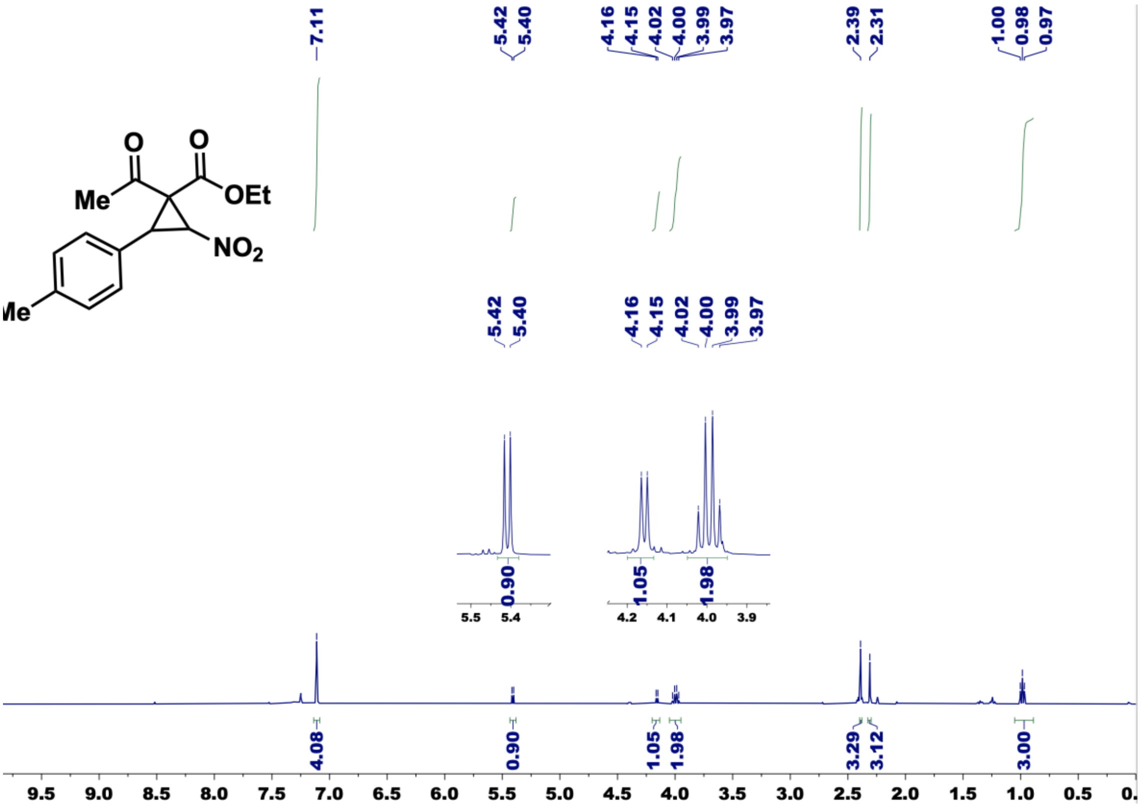

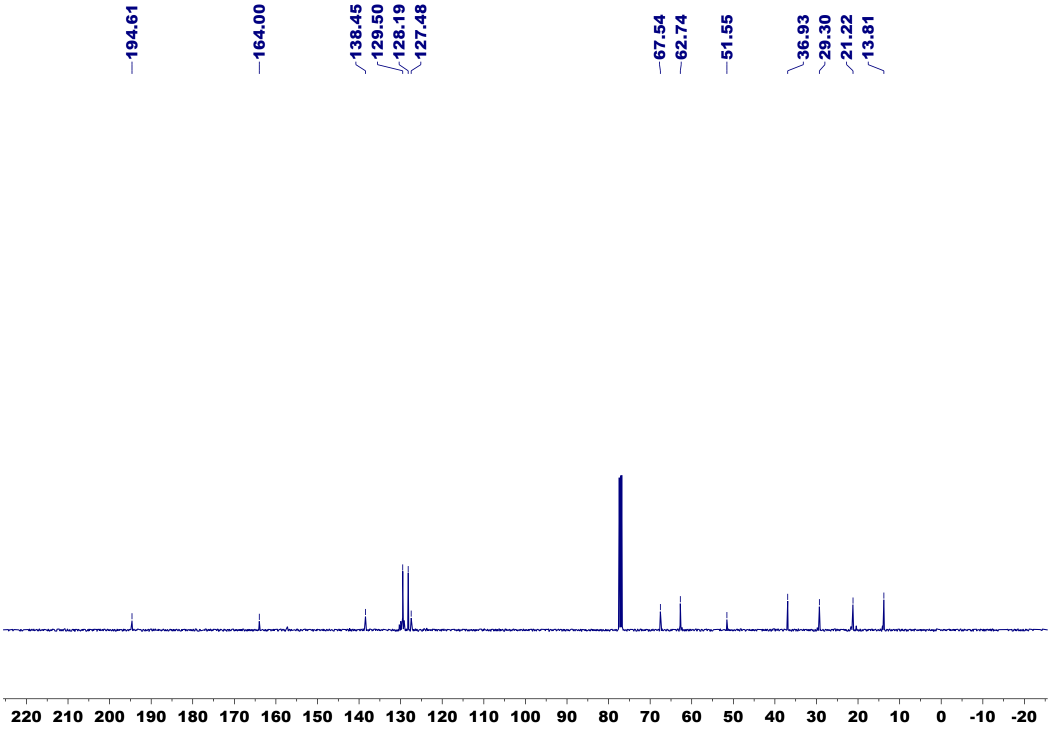

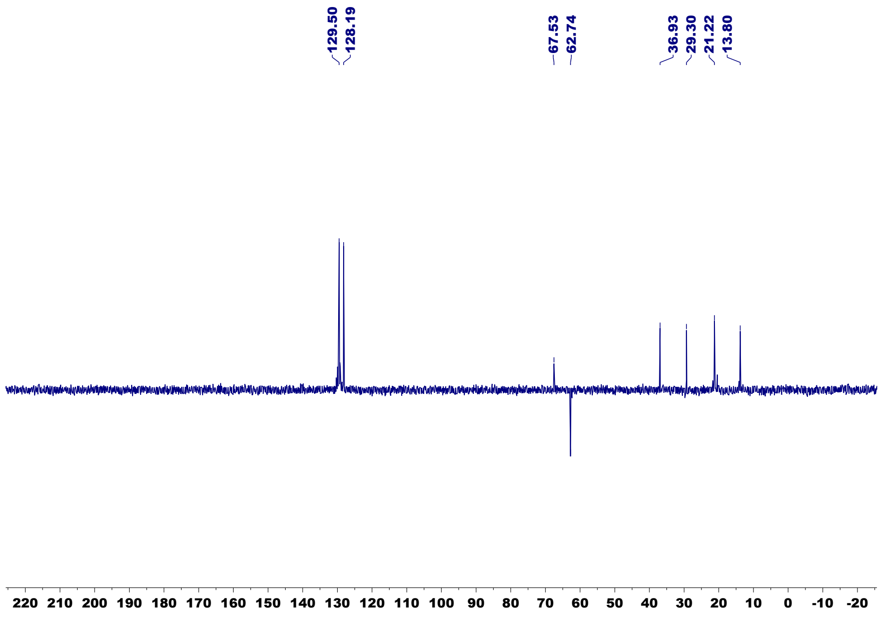

Methyl 2-(4-methylphenyl)-3-nitro1-propanoylcyclopropane-1-carboxylate (1d)
($CDCl_3$)

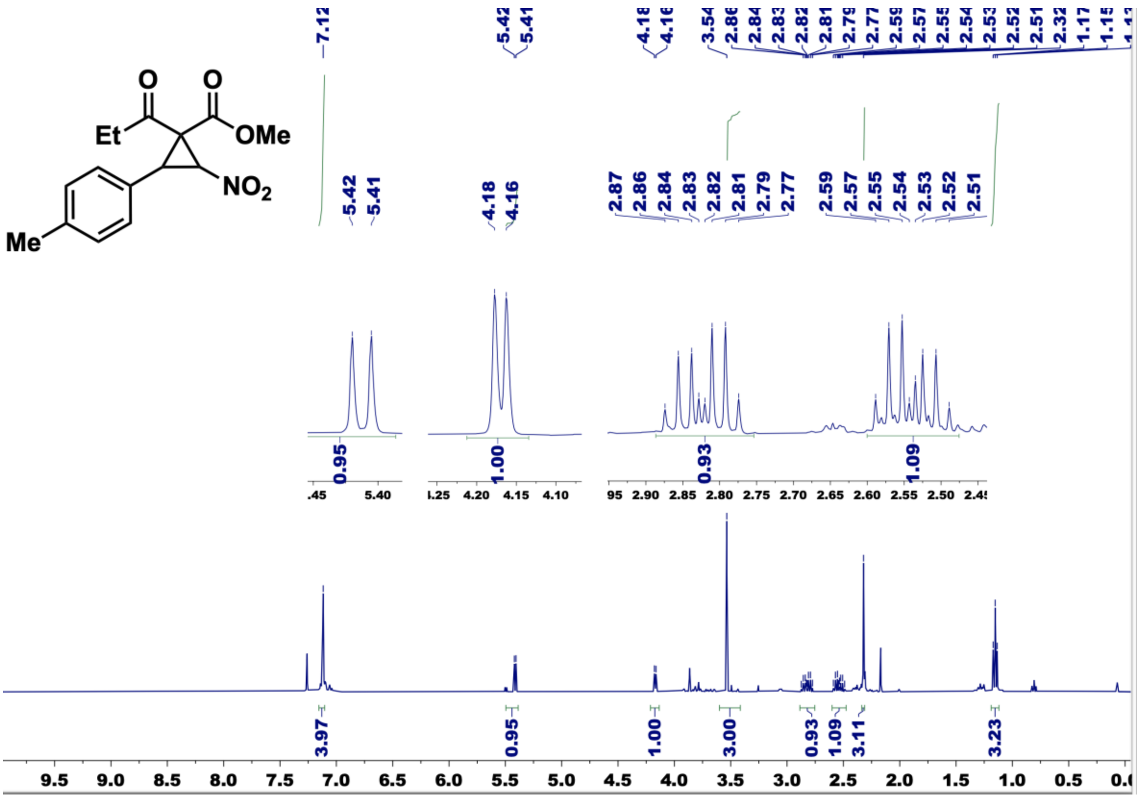

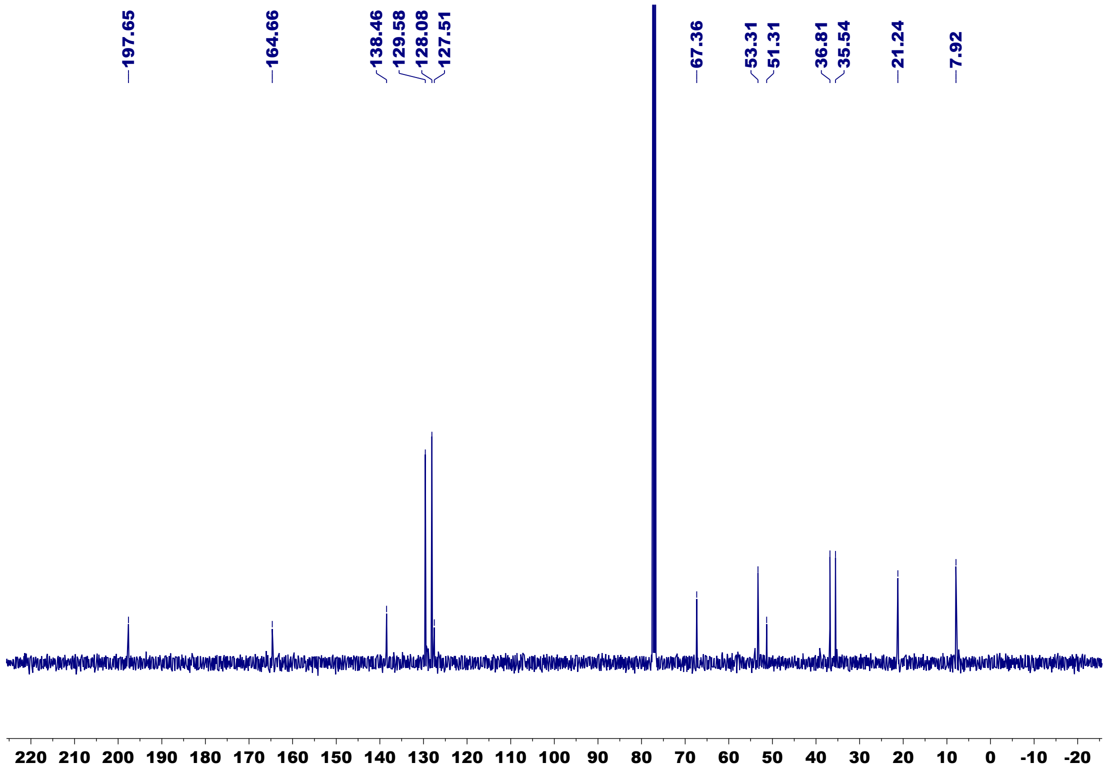
197.65
164.66
138.46
129.58
128.08
127.51
67.36
53.31
51.31
36.81
35.54
21.24
7.92
220 210 200 190 180 170 160 150 140 130 120 110 100 90 80 70 60 50 40 30 20 10 0 -10 -20

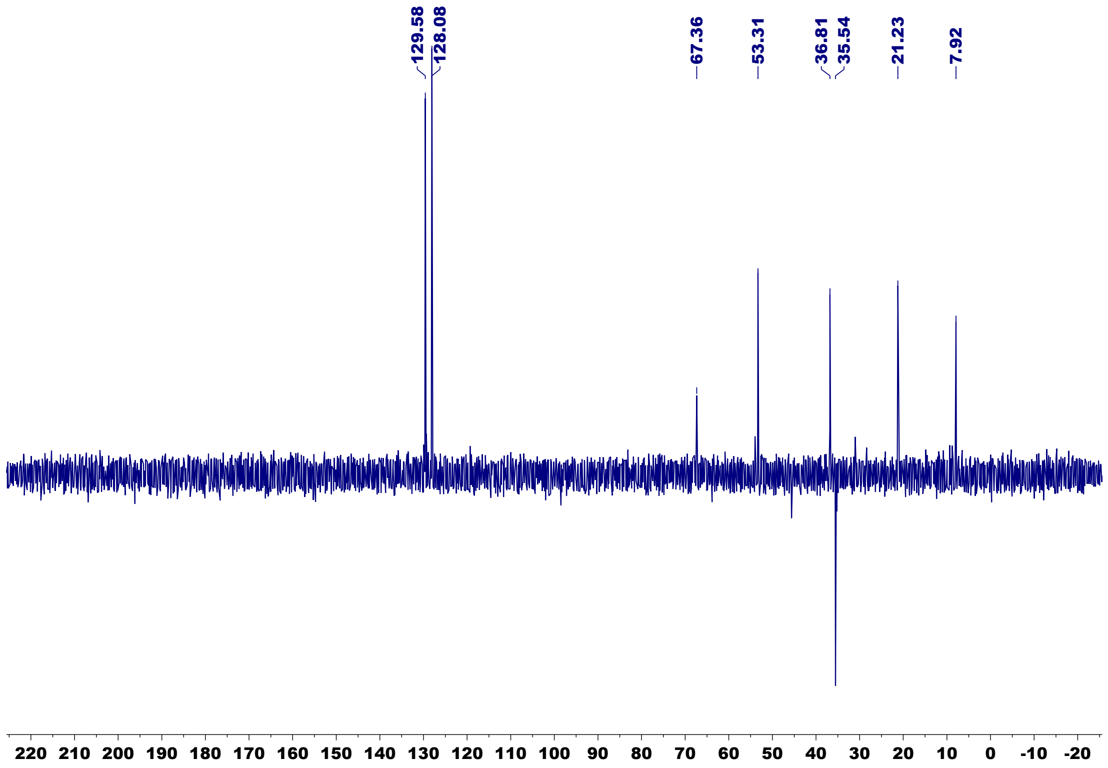
129.58
128.08
67.36
53.31
36.81
35.54
21.23
7.92
220 210 200 190 180 170 160 150 140 130 120 110 100 90 80 70 60 50 40 30 20 10 0 -10 -20

Methyl 2-(4-methylphenyl)-1-(2-methylpropanoyl)-3-nitrocyclopropane-1-carboxylate (1e) ($CDCl_3$)

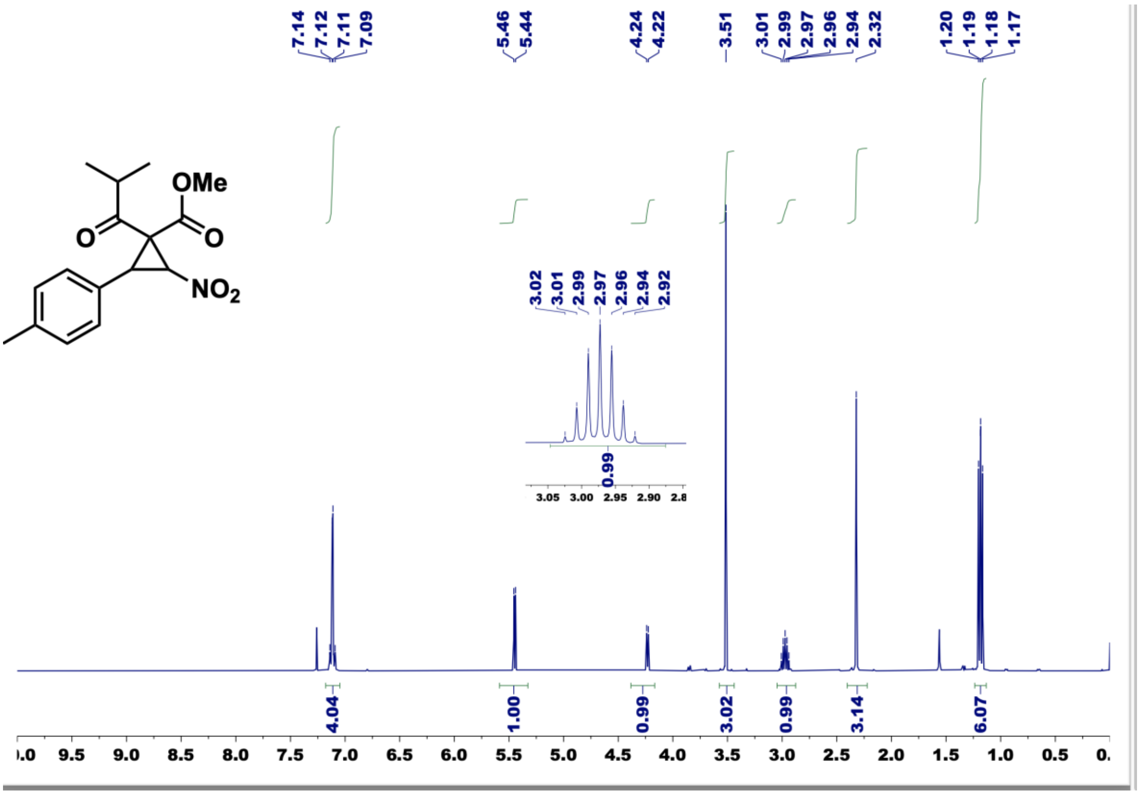

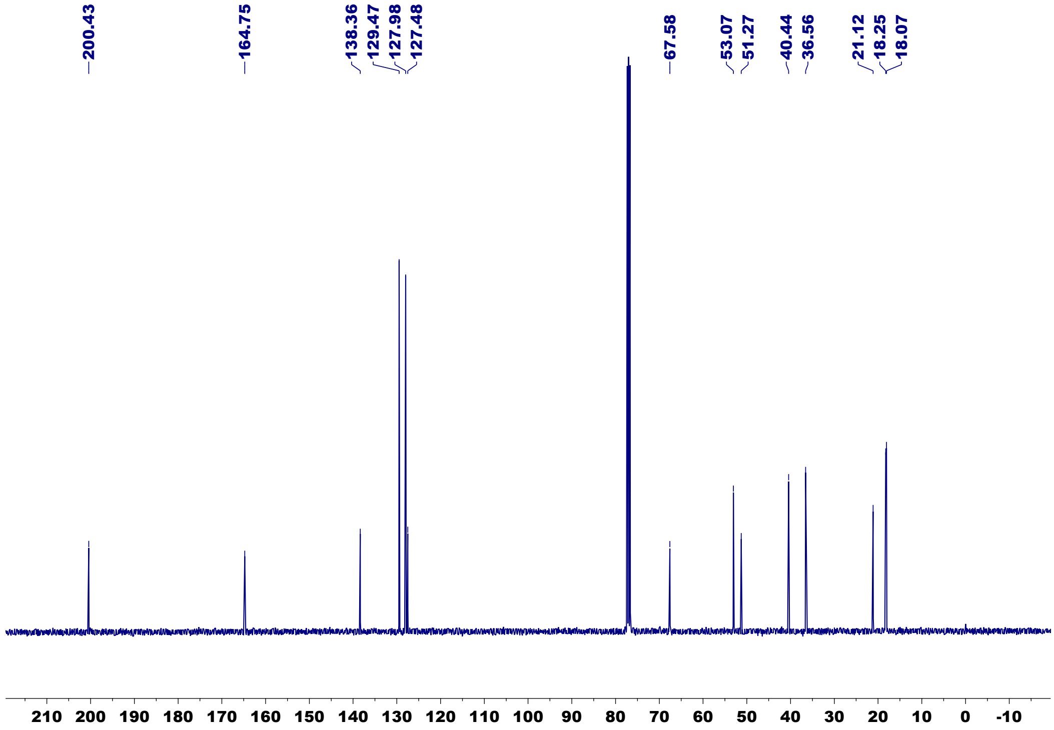

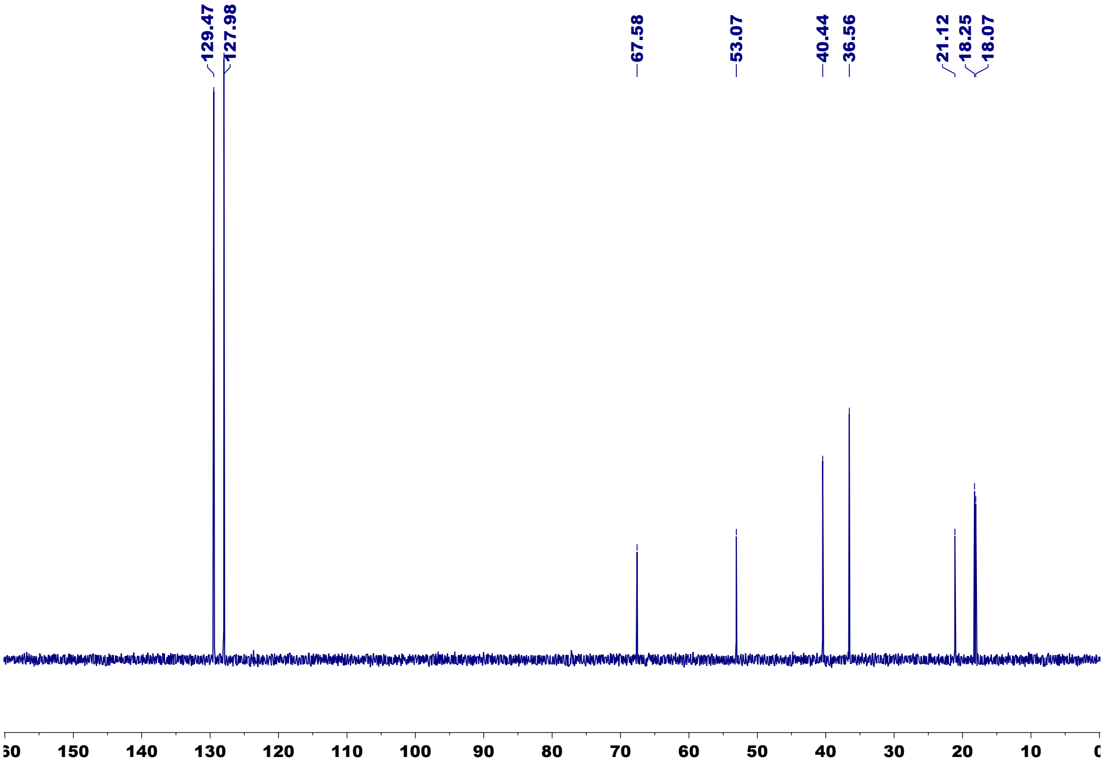

Ethyl 1-benzoyl-2-(4-methylphenyl)-3-nitrocyclopropanecarboxylate (1f) ($CDCl_3$)

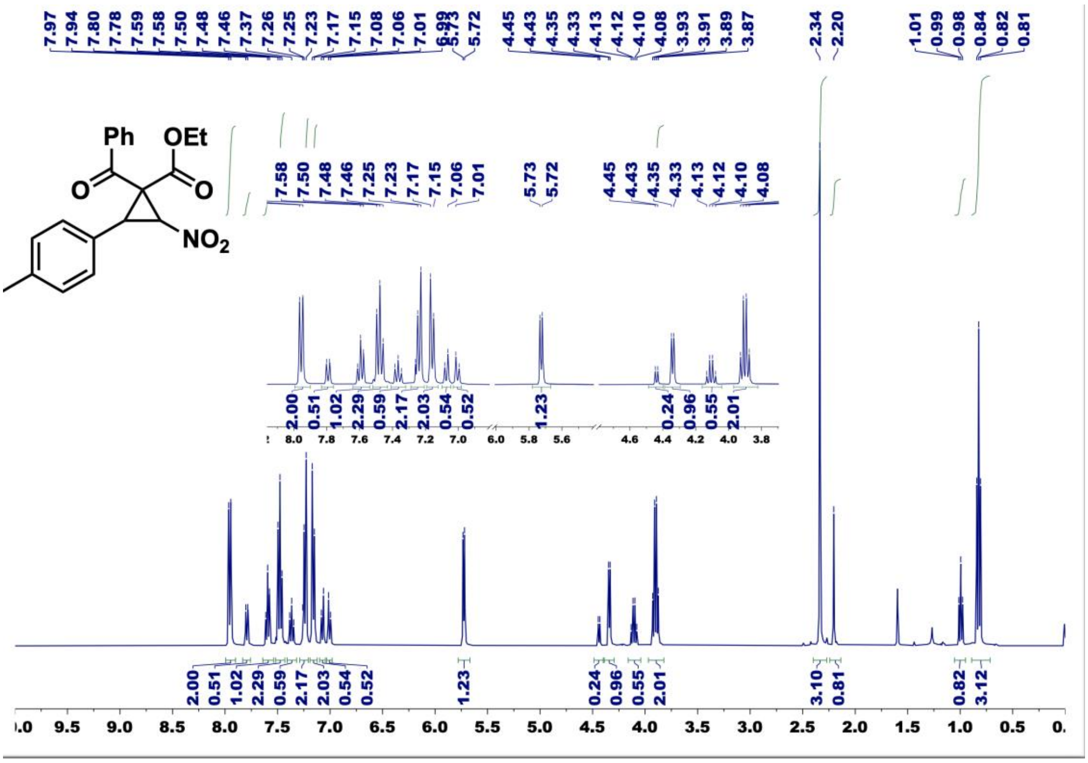

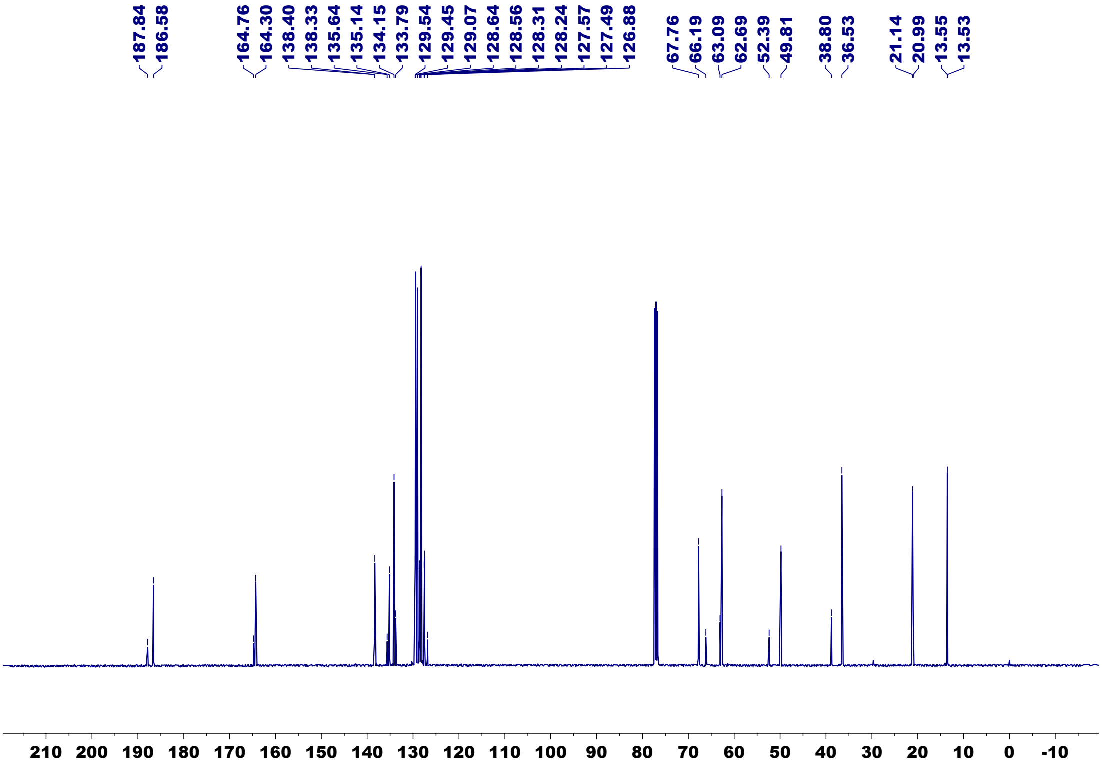
187.84
186.58
164.76
164.30
138.40
138.33
135.64
135.14
134.15
133.79
129.54
129.45
129.07
128.64
128.56
128.31
128.24
127.57
127.49
126.88
67.76
66.19
63.09
62.69
52.39
49.81
38.80
36.53
21.14
20.99
13.55
13.53
210 200 190 180 170 160 150 140 130 120 110 100 90 80 70 60 50 40 30 20 10 0 -10

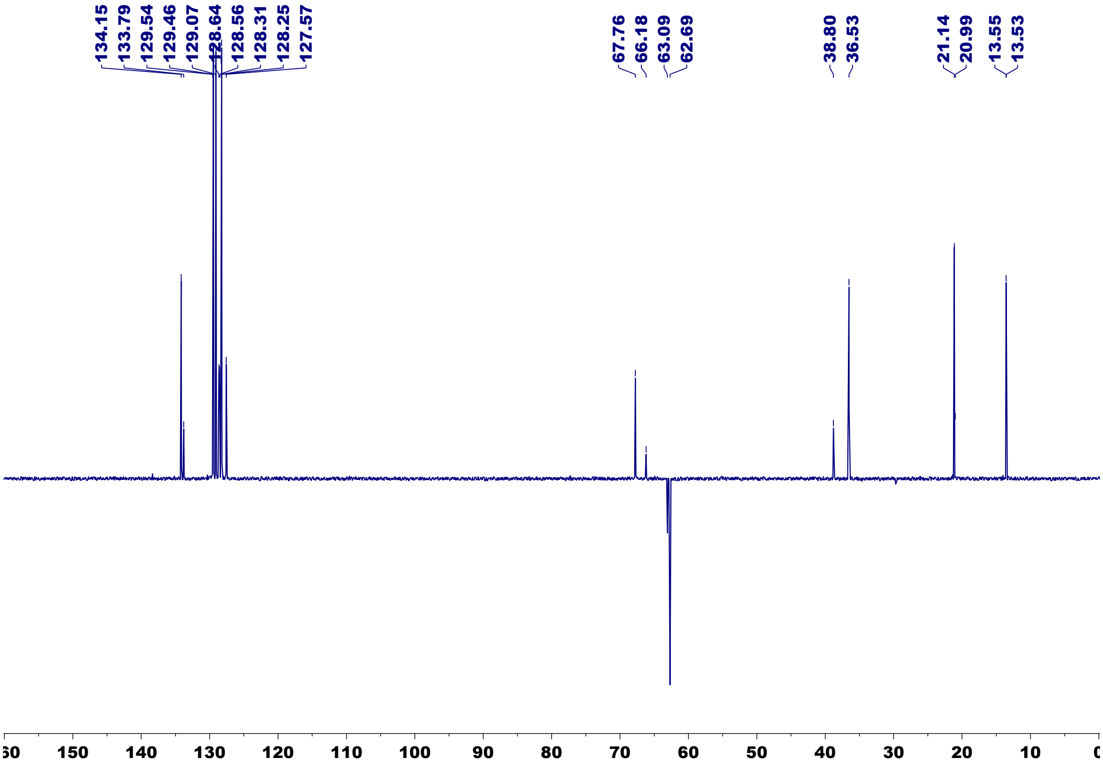
134.15
133.79
129.54
129.46
129.07
128.64
128.56
128.31
128.25
127.57
67.76
66.18
63.09
62.69
38.80
36.53
21.14
20.99
13.55
13.53
150 140 130 120 110 100 90 80 70 60 50 40 30 20 10

1-Benzoyl-1-ethanoyl-2-(4-methylphenyl)-3-nitrocyclopropane (1g) (CDCl$_3$)

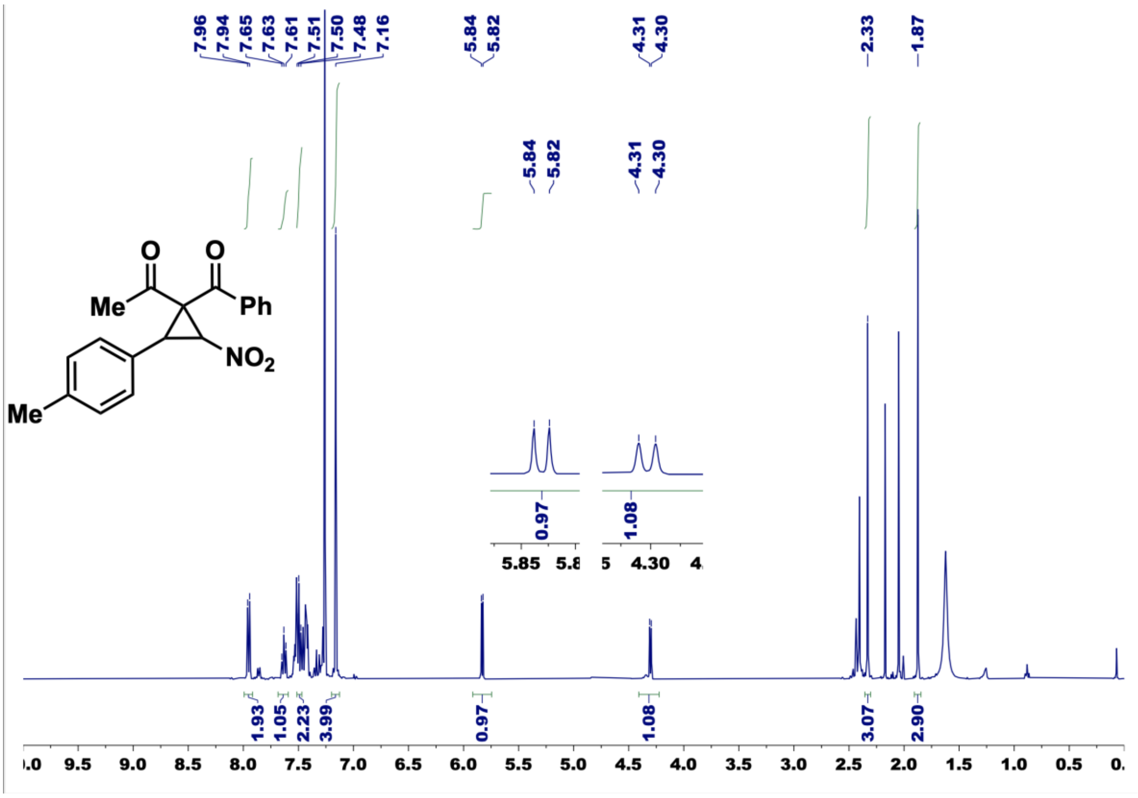

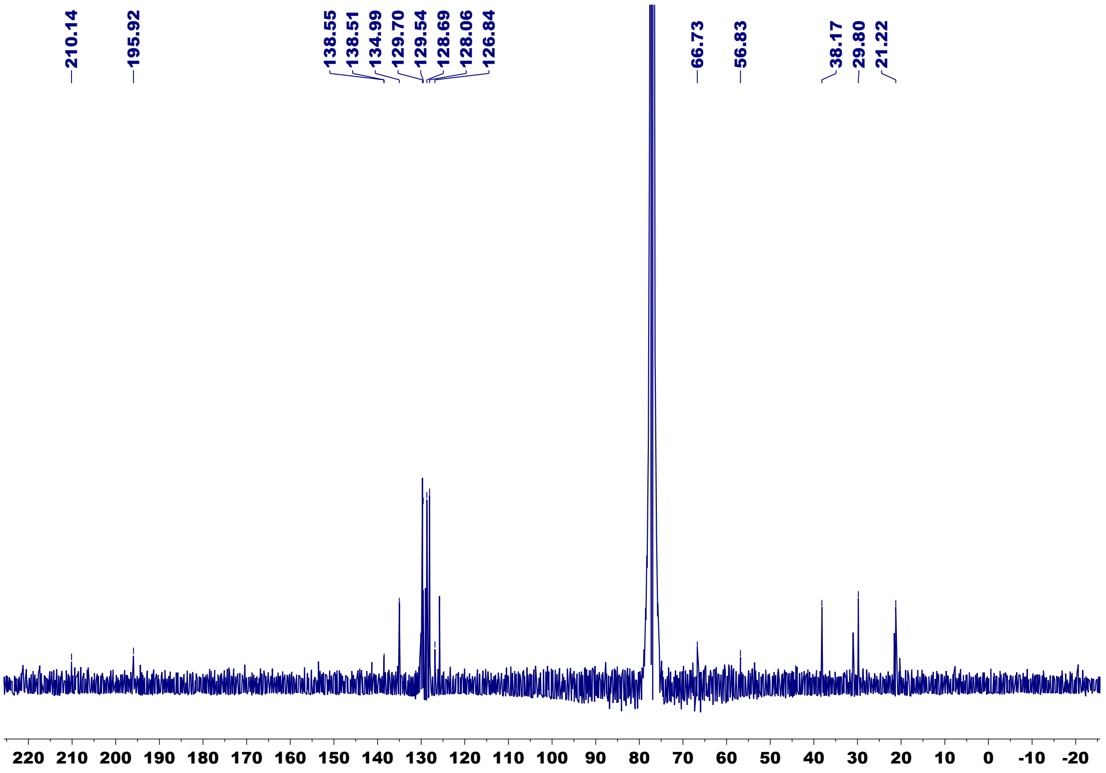

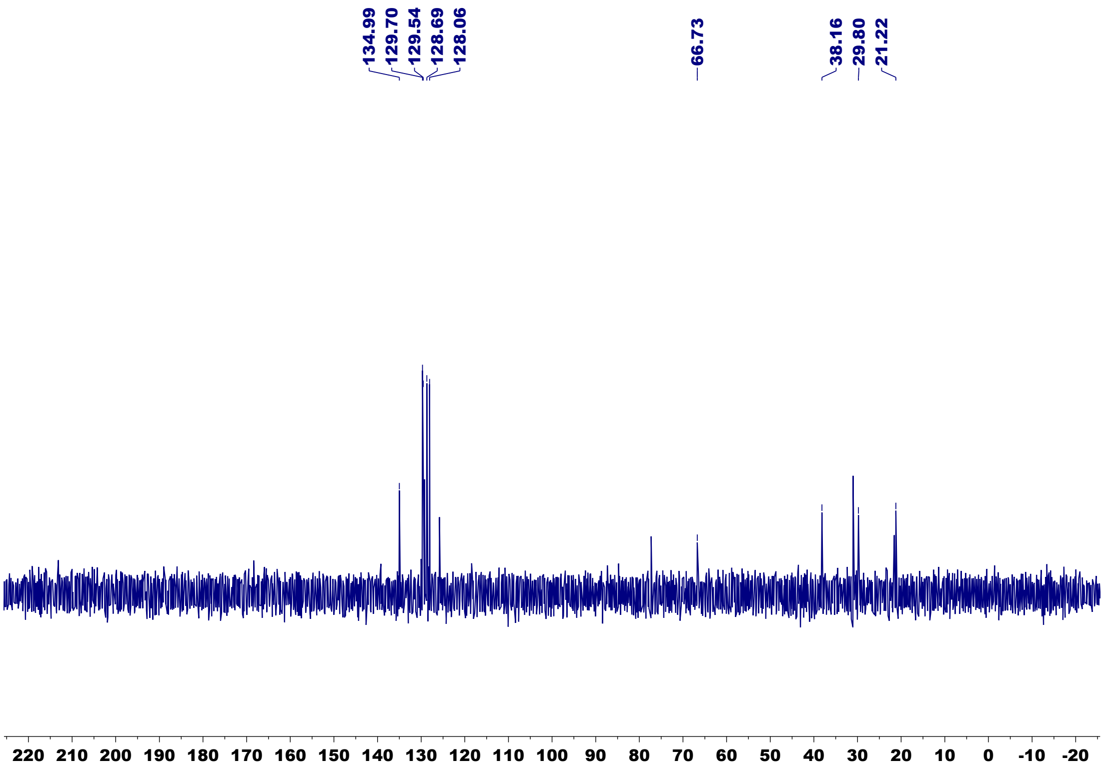

3-Ethanoyl-4,5-dihydro-2-methyl-4-(4-methylphenyl)-5-nitrofuran (8b) ($CDCl_3$)

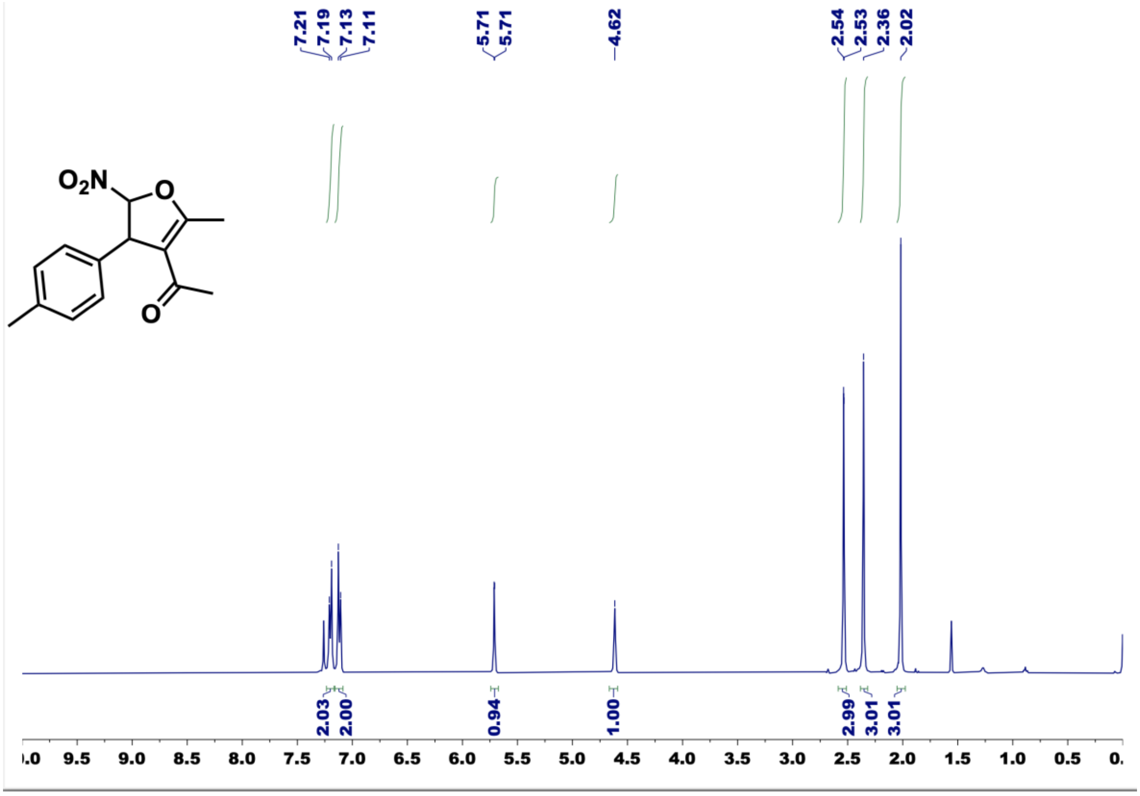

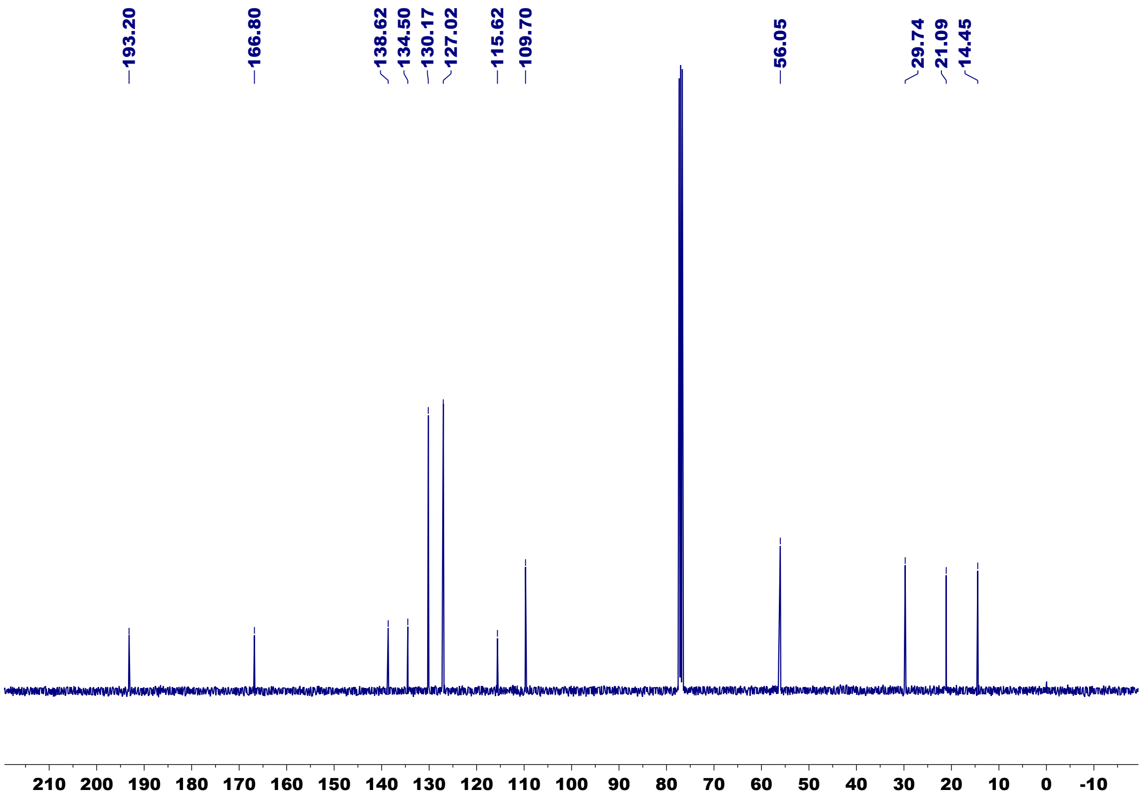
193.20
166.80
138.62
134.50
130.17
127.02
115.62
109.70
56.05
29.74
21.09
14.45
210 200 190 180 170 160 150 140 130 120 110 100 90 80 70 60 50 40 30 20 10 0 -10

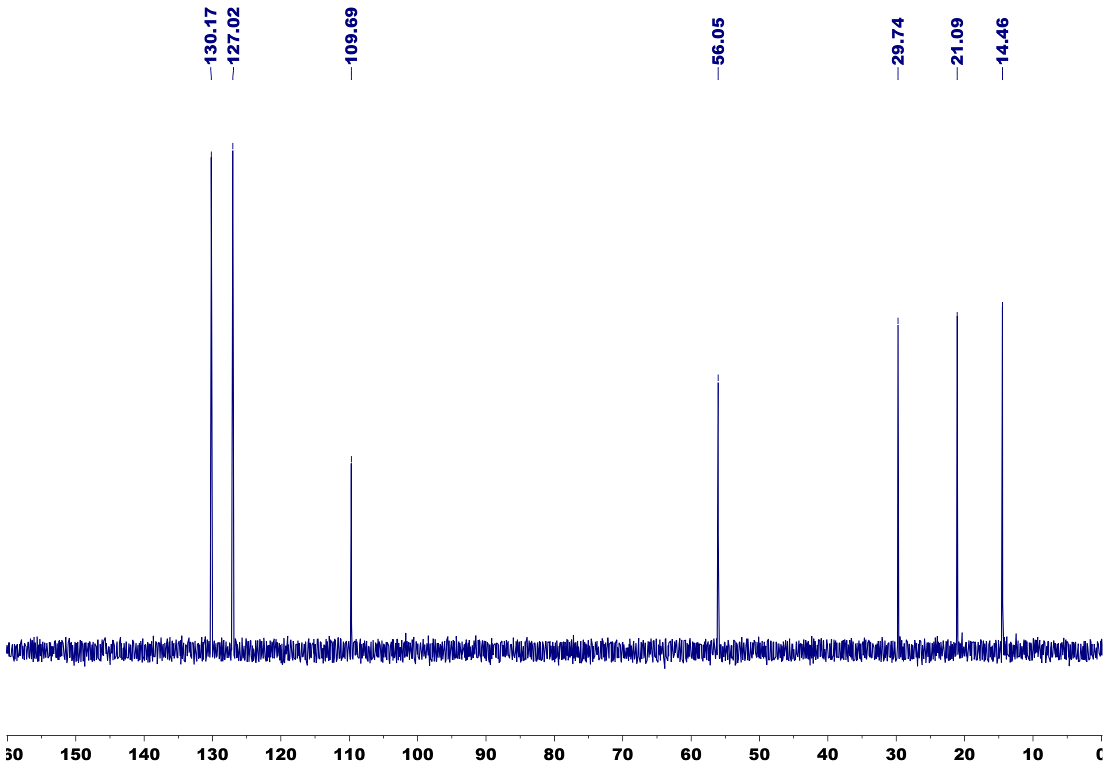
130.17
127.02
109.69
56.05
29.74
21.09
14.46
150 140 130 120 110 100 90 80 70 60 50 40 30 20 10

1-Ethyl 4,5-dihydro-2-methyl-4-(4-methylphenyl)-5-nitrofuran-3-carboxylate (8c)
($CDCl_3$)

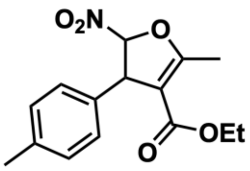

7b

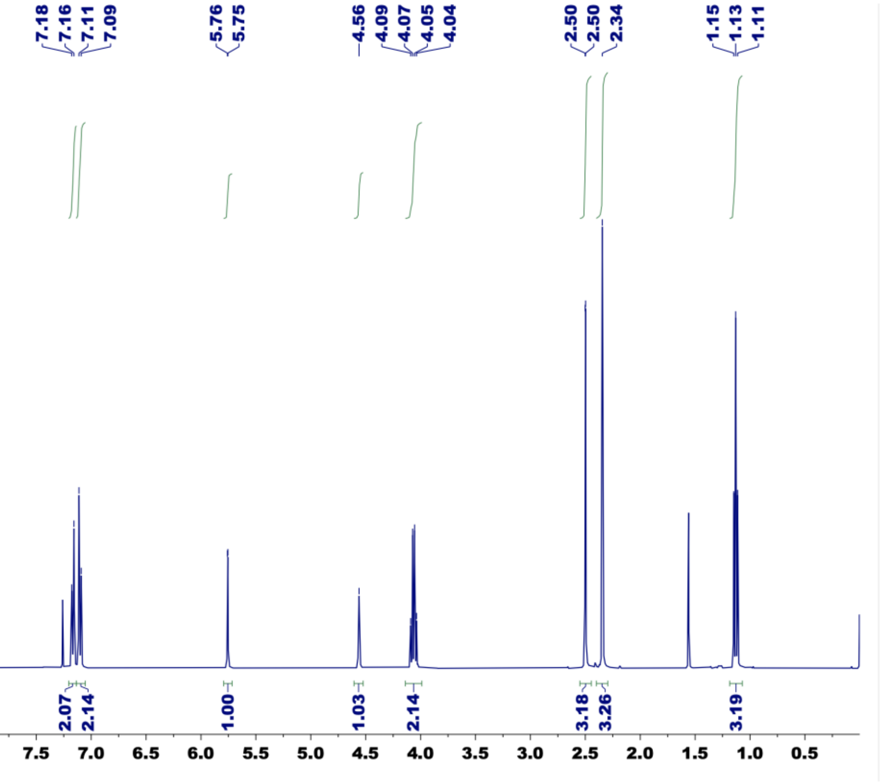

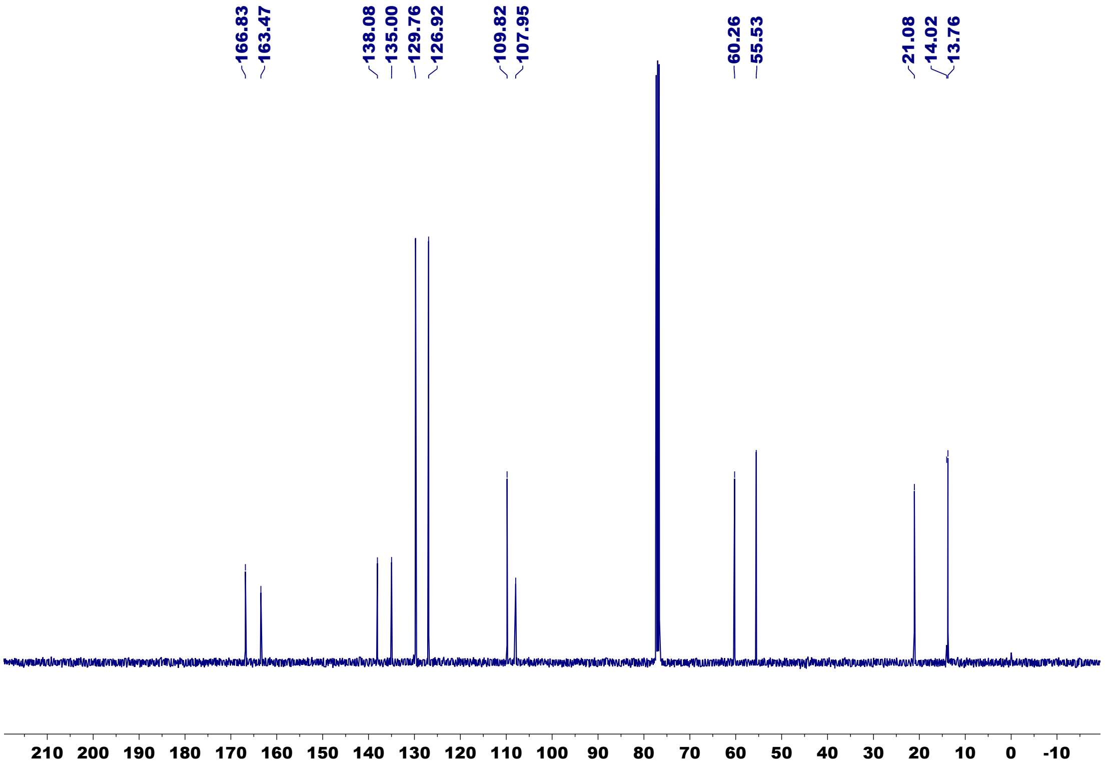

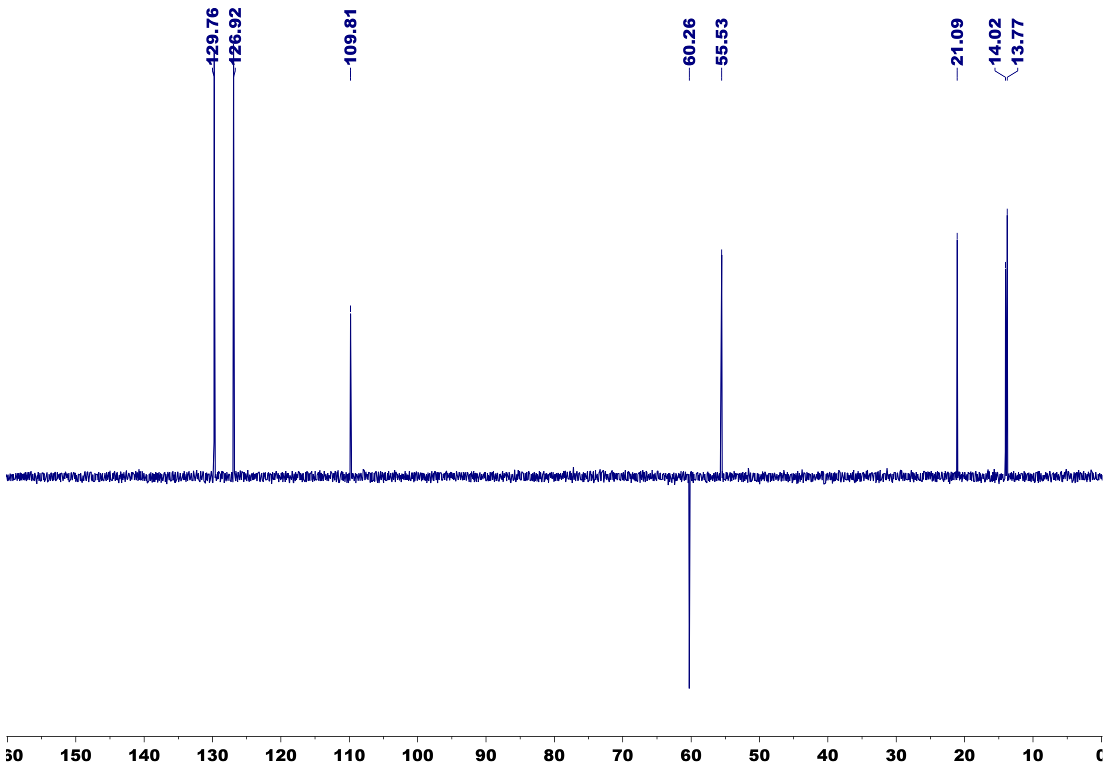

Methyl 2-ethyl-4,5-dihydro-4-(4-methylphenyl)-5-nitrofuran-3-carboxylate (8d)
($CDCl_3$)

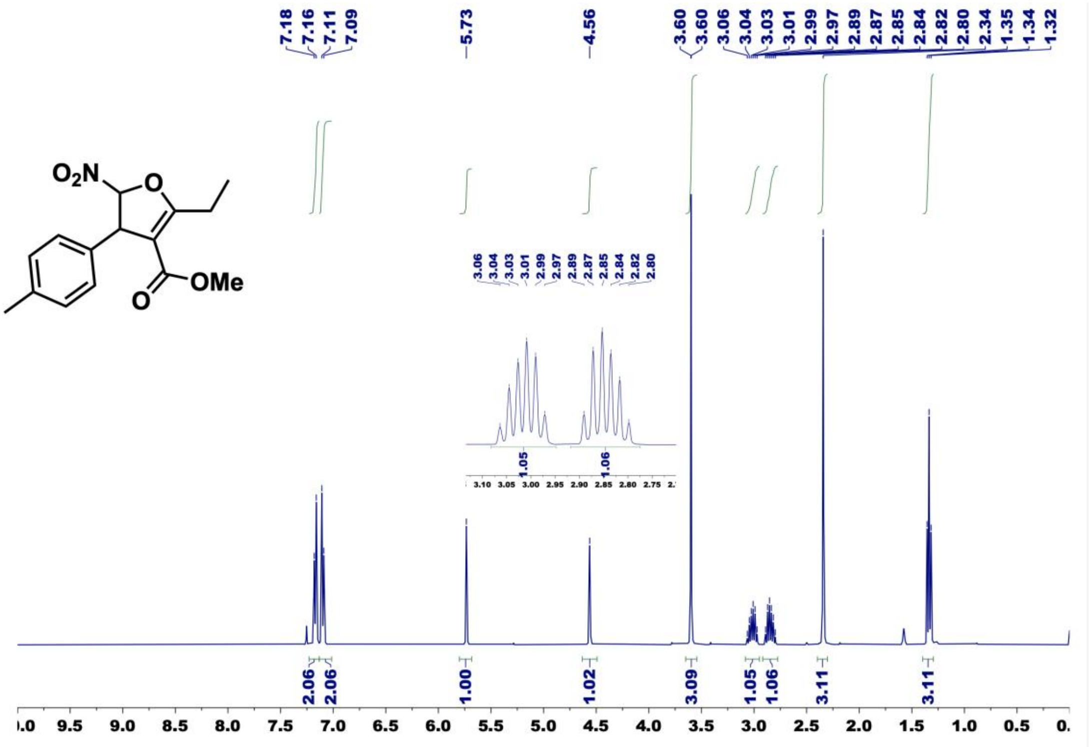

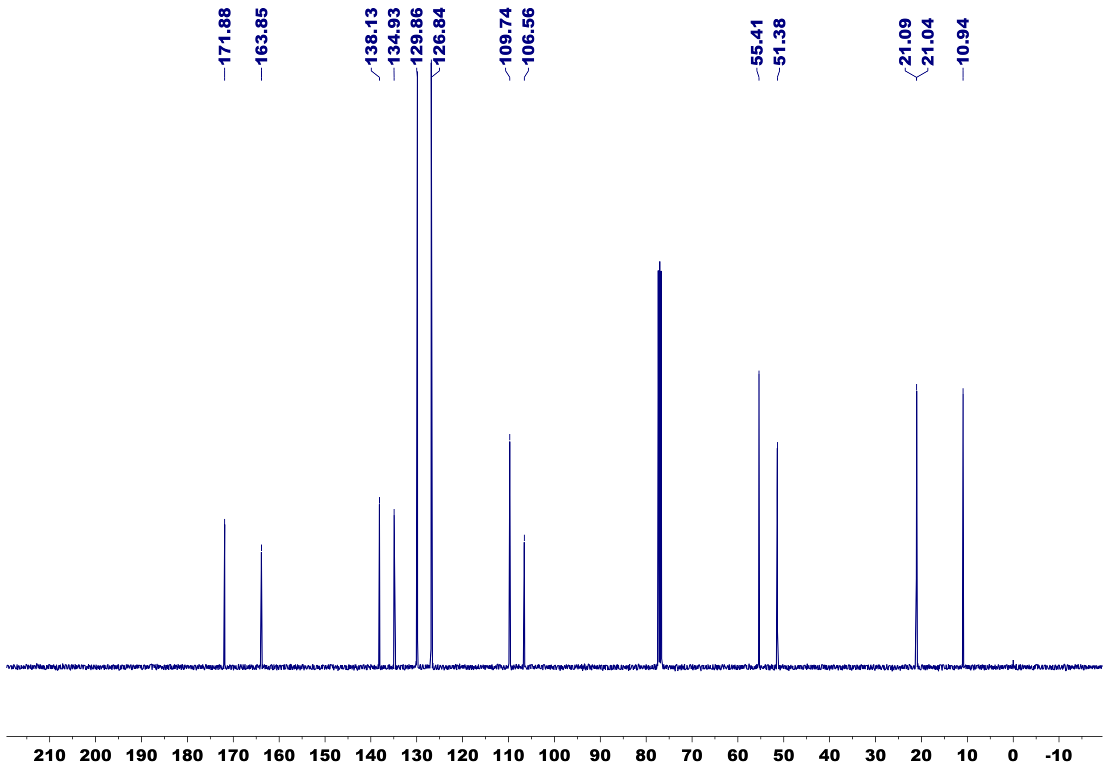
171.88
163.85
138.13
134.93
129.86
126.84
109.74
106.56
55.41
51.38
21.09
21.04
10.94
210 200 190 180 170 160 150 140 130 120 110 100 90 80 70 60 50 40 30 20 10 0 -10

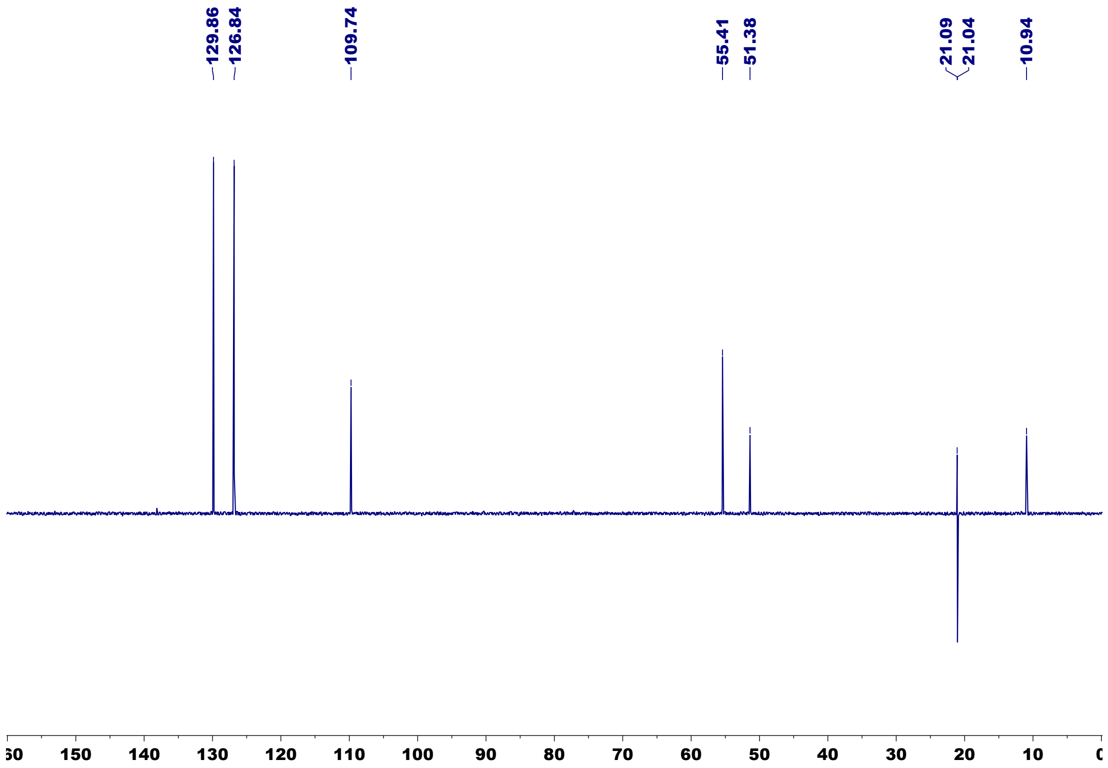
129.86
126.84
109.74
55.41
51.38
21.09
21.04
10.94
150 140 130 120 110 100 90 80 70 60 50 40 30 20 10

Methyl 4,5-dihydro-2-(1-methylethyl)-4-(4-methylphenyl)-5-nitrofuran-3-carboxylate (8e) ($CDCl_3$)

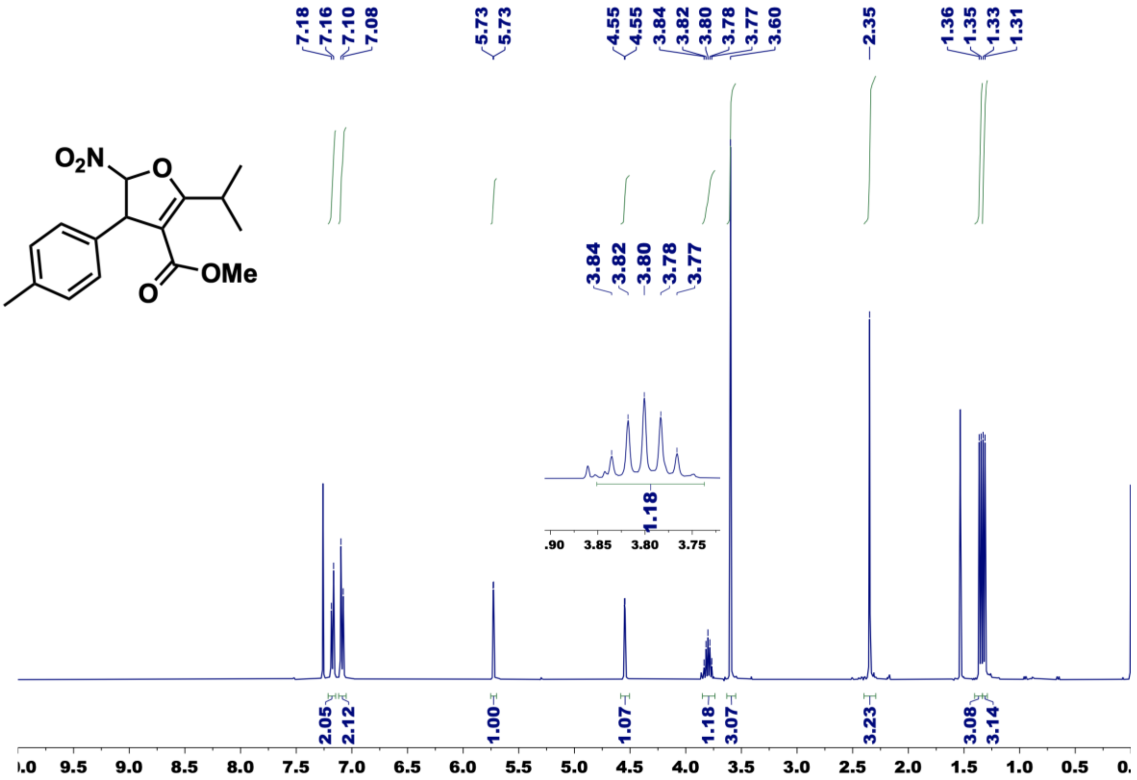

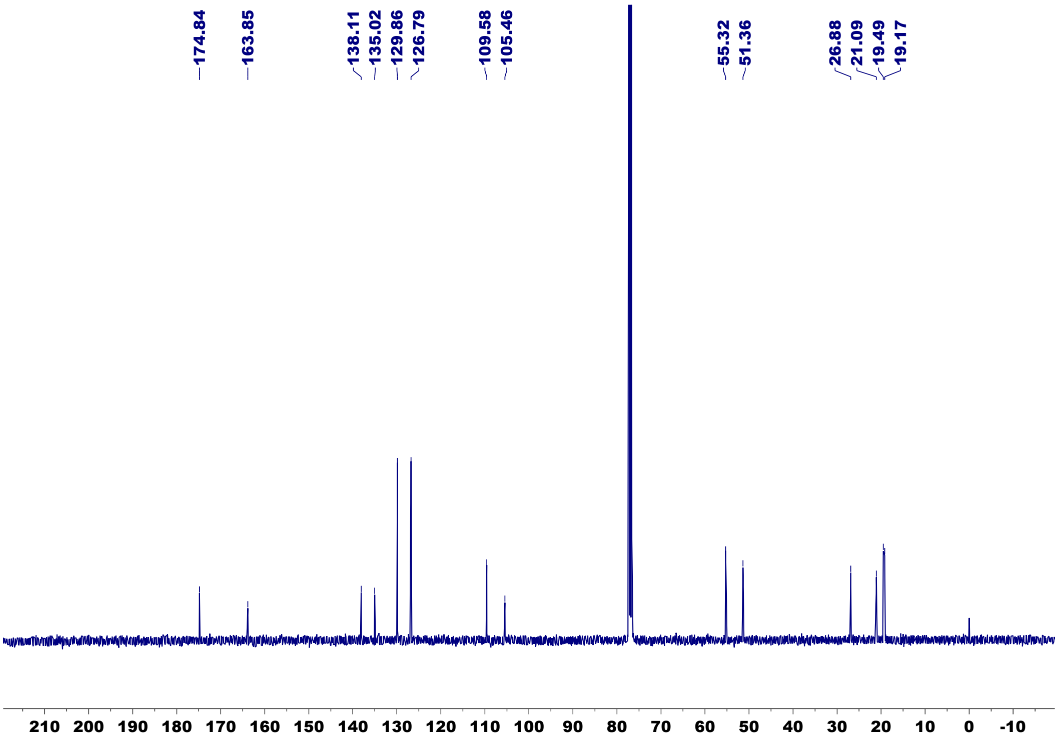

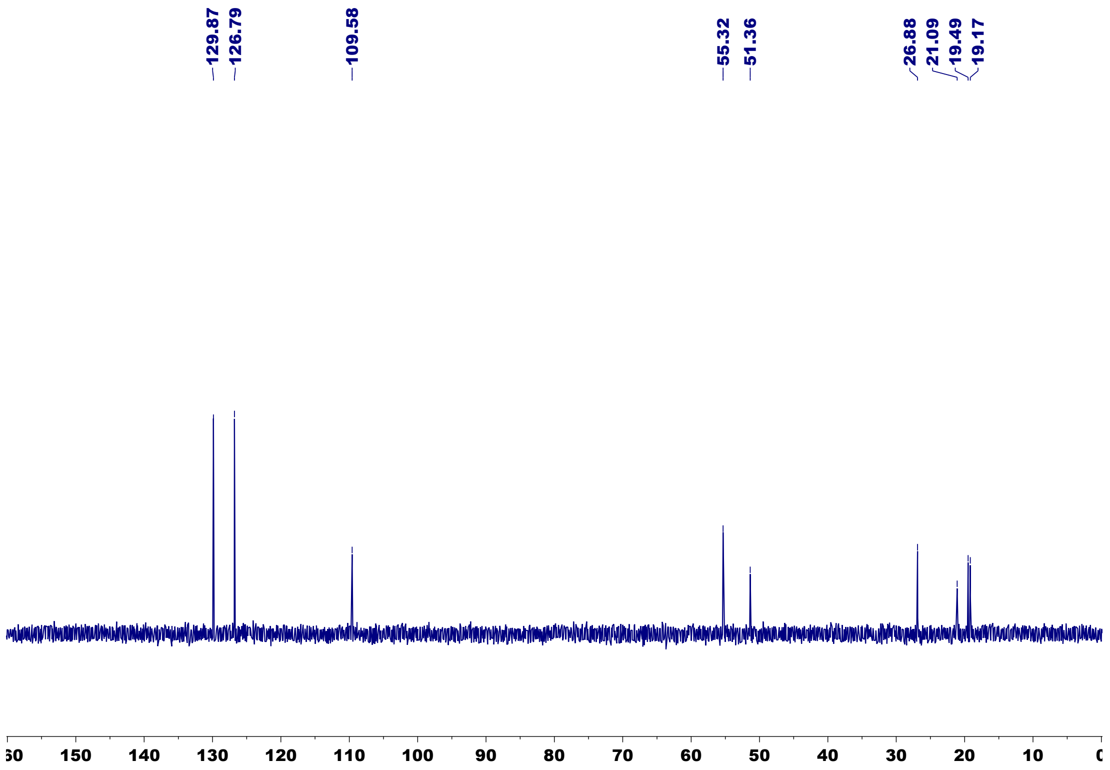

Methyl 5-isopropyl-3-(4-methylphenyl)-2-nitro-4-carboxylate (11e) ($CDCl_3$)

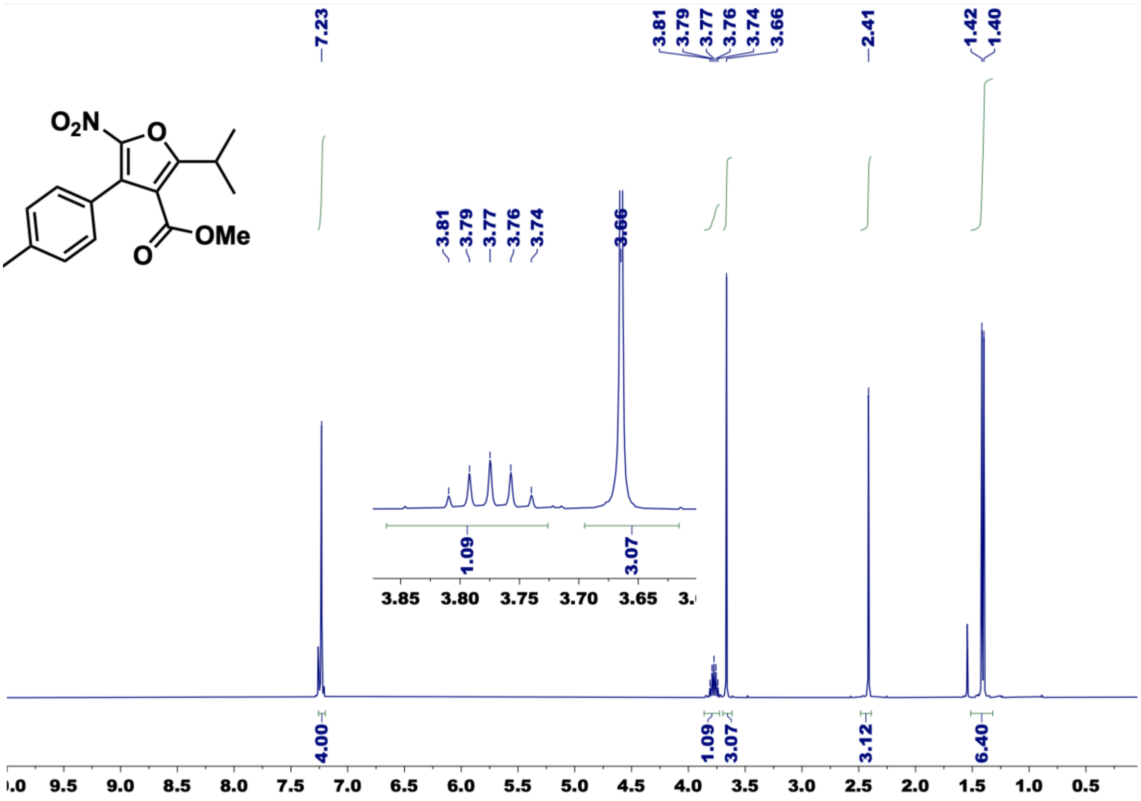

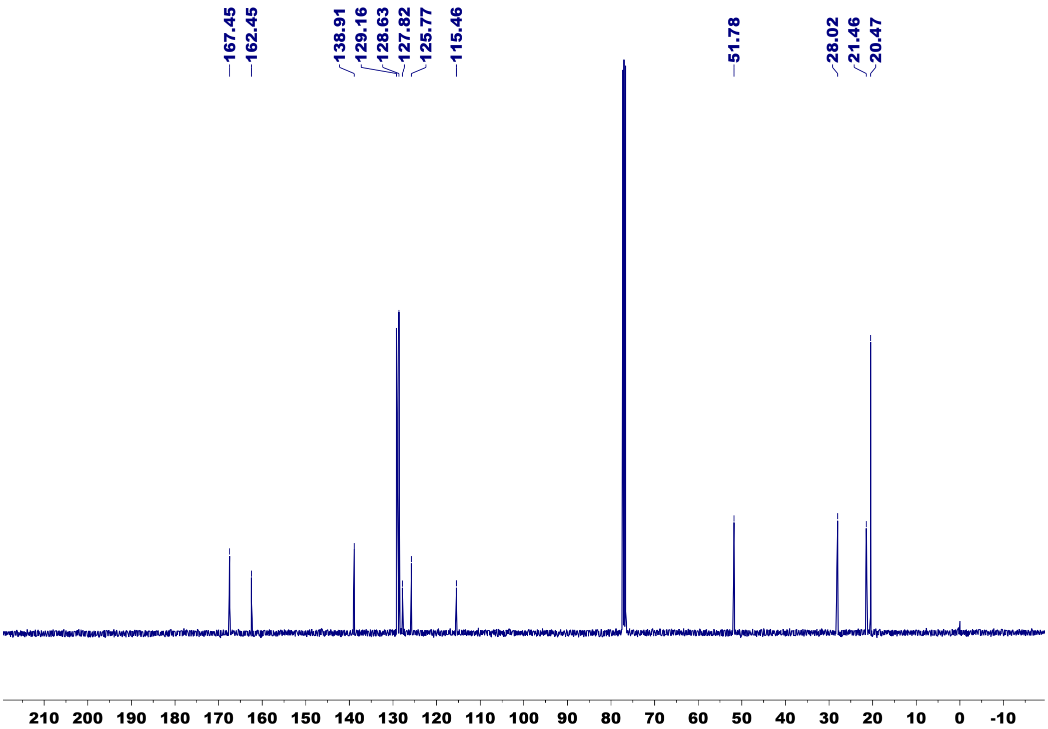
167.45
162.45
138.91
129.16
128.63
127.82
125.77
115.46
51.78
28.02
21.46
20.47
210 200 190 180 170 160 150 140 130 120 110 100 90 80 70 60 50 40 30 20 10 0 -10

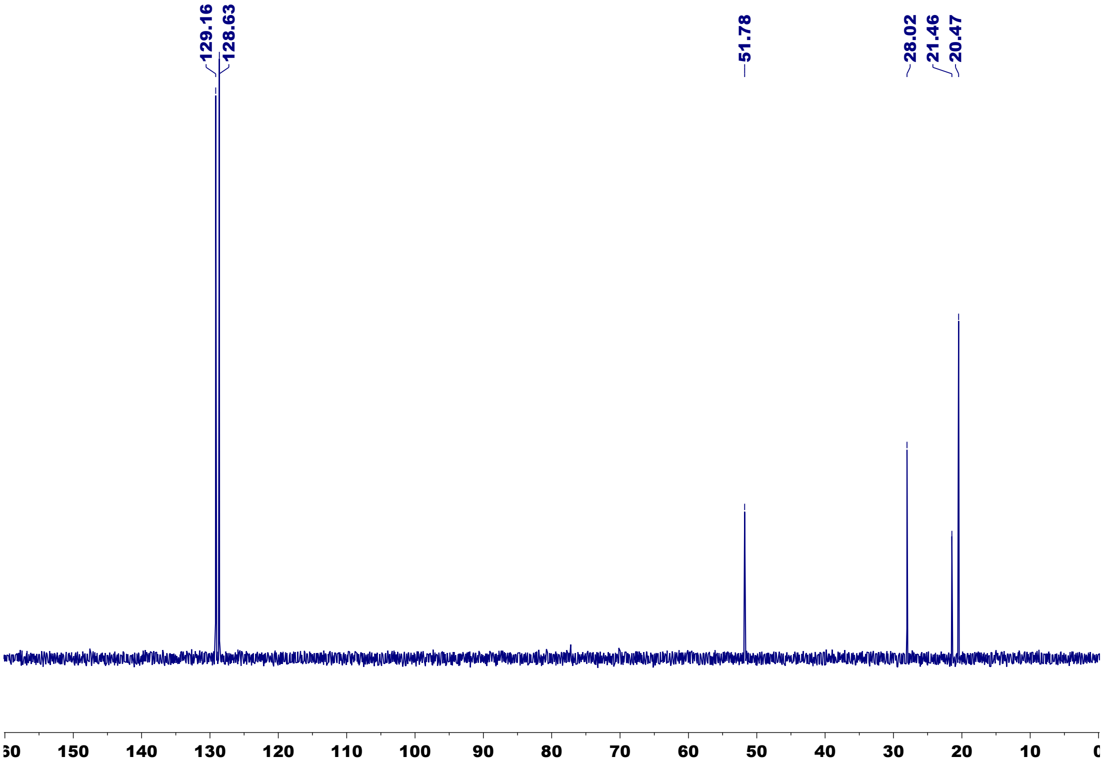
129.16
128.63
51.78
28.02
21.46
20.47
150 140 130 120 110 100 90 80 70 60 50 40 30 20 10

Ethyl 4,5-dihydro-4-(4-methylphenyl)-5-nitro-2-phenylfuran-3-carboxylate (8f)
($CDCl_3$)

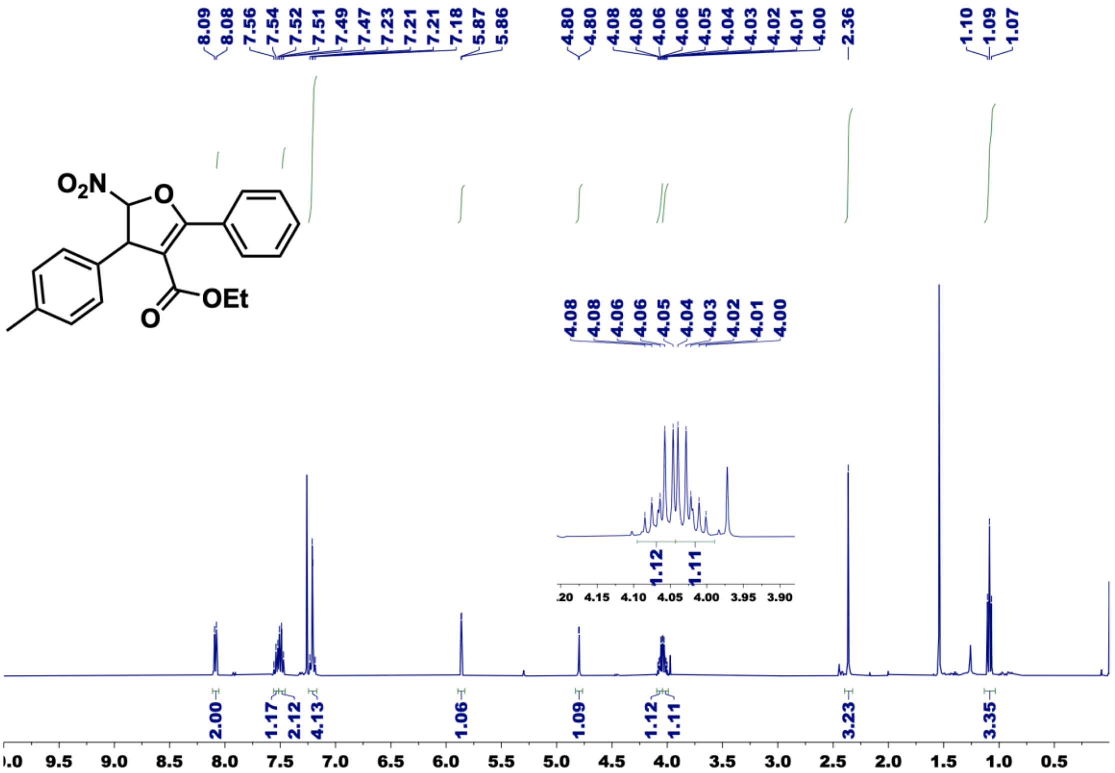

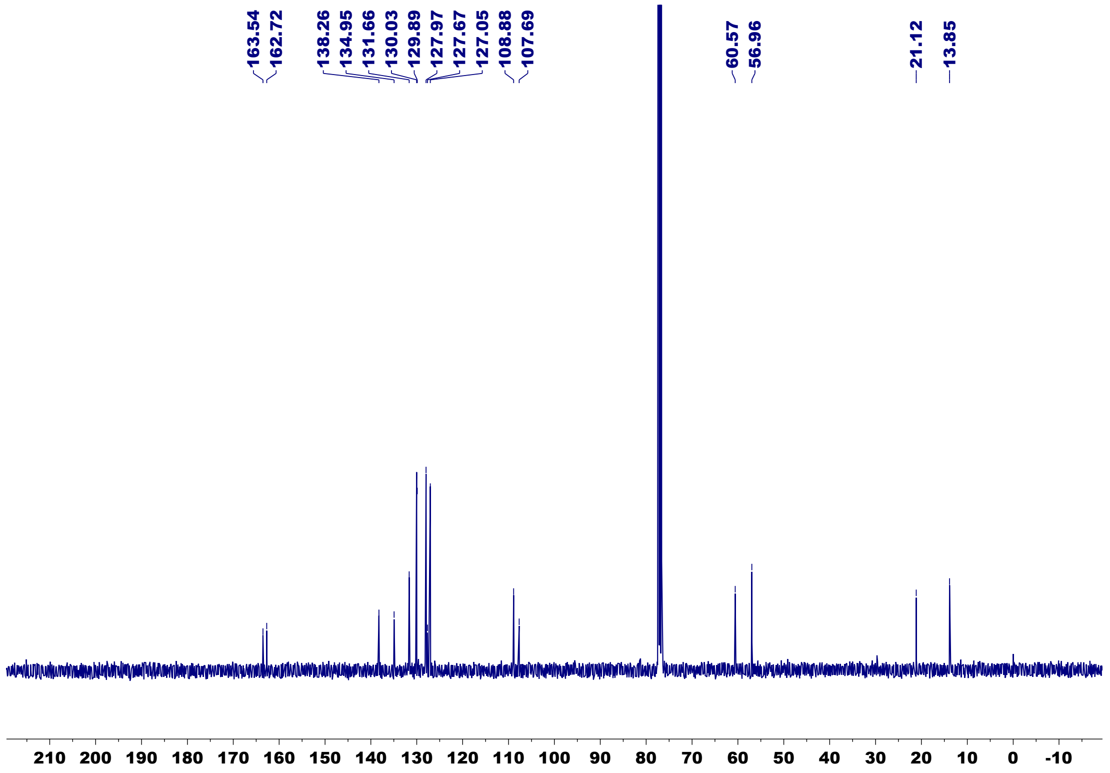

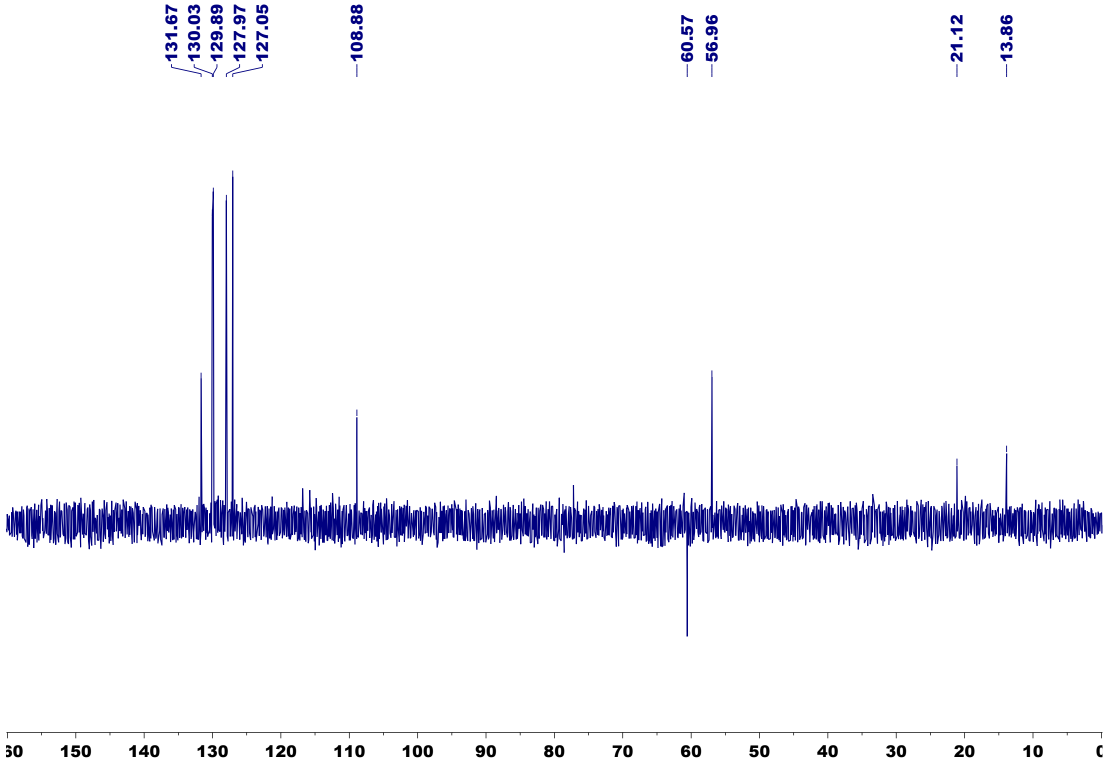

Ethyl 4-(4-methylphenyl)-5-nitro-2-phenylfuran-3-carboxylate (11f)

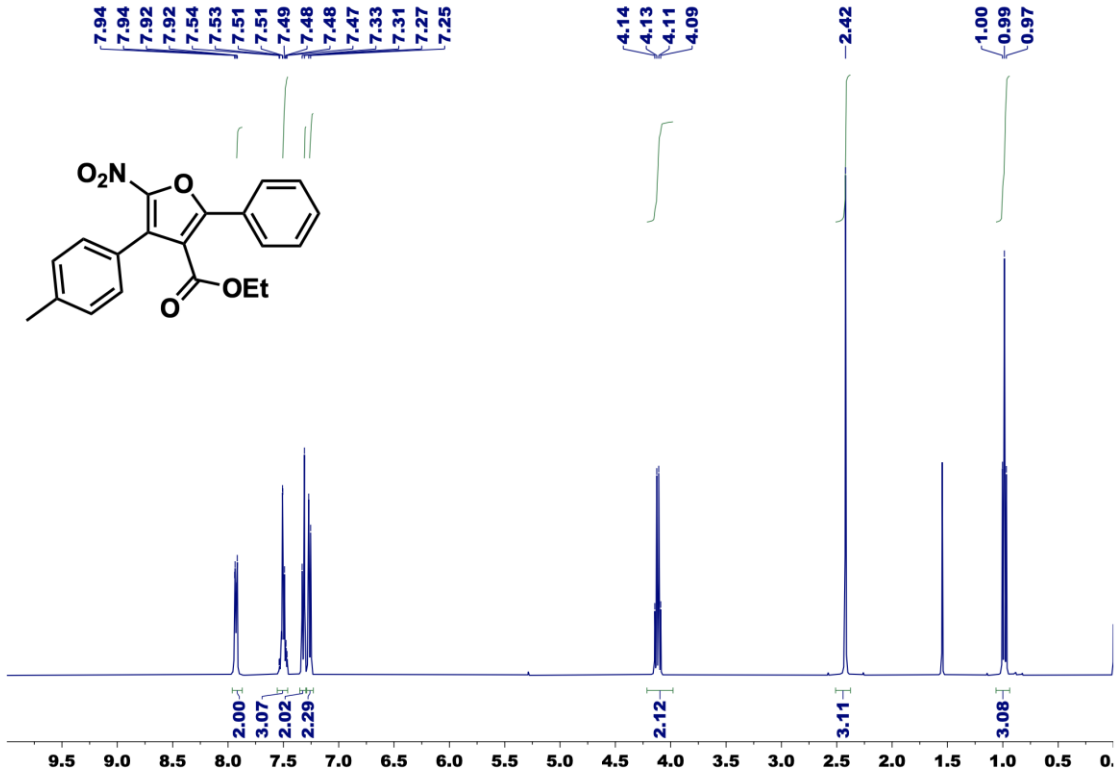

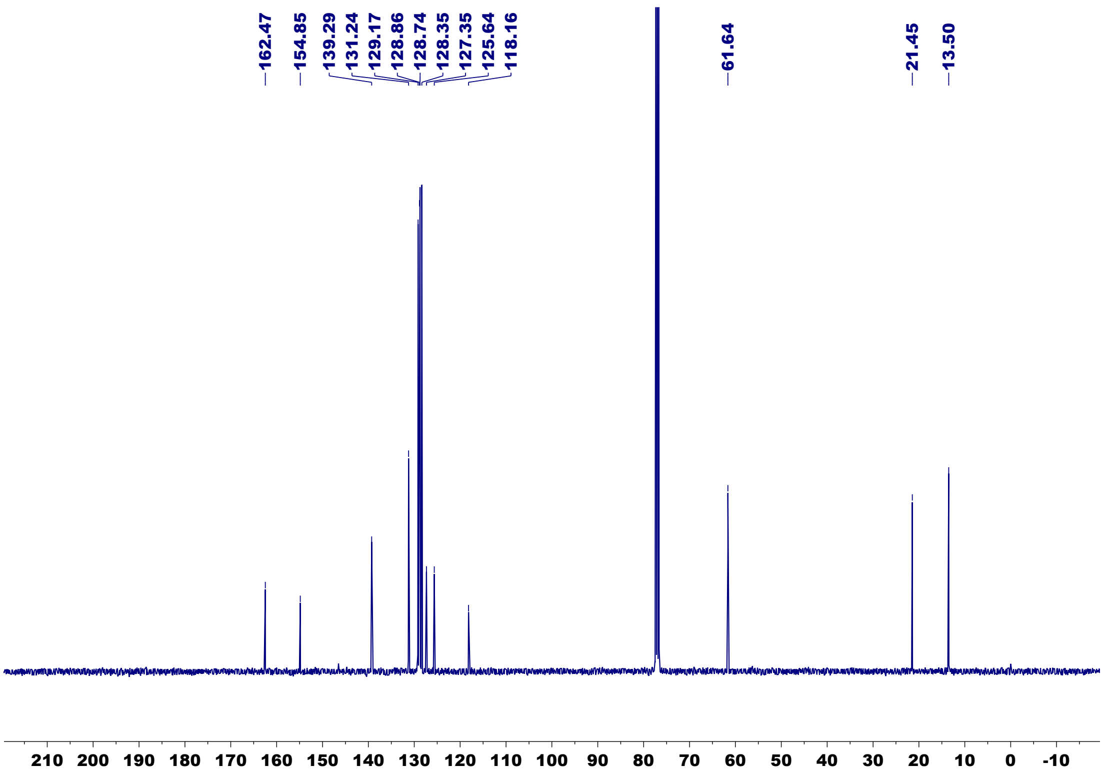
162.47
154.85
139.29
131.24
129.17
128.86
128.74
128.35
127.35
125.64
118.16
61.64
21.45
13.50
210 200 190 180 170 160 150 140 130 120 110 100 90 80 70 60 50 40 30 20 10 0 -10

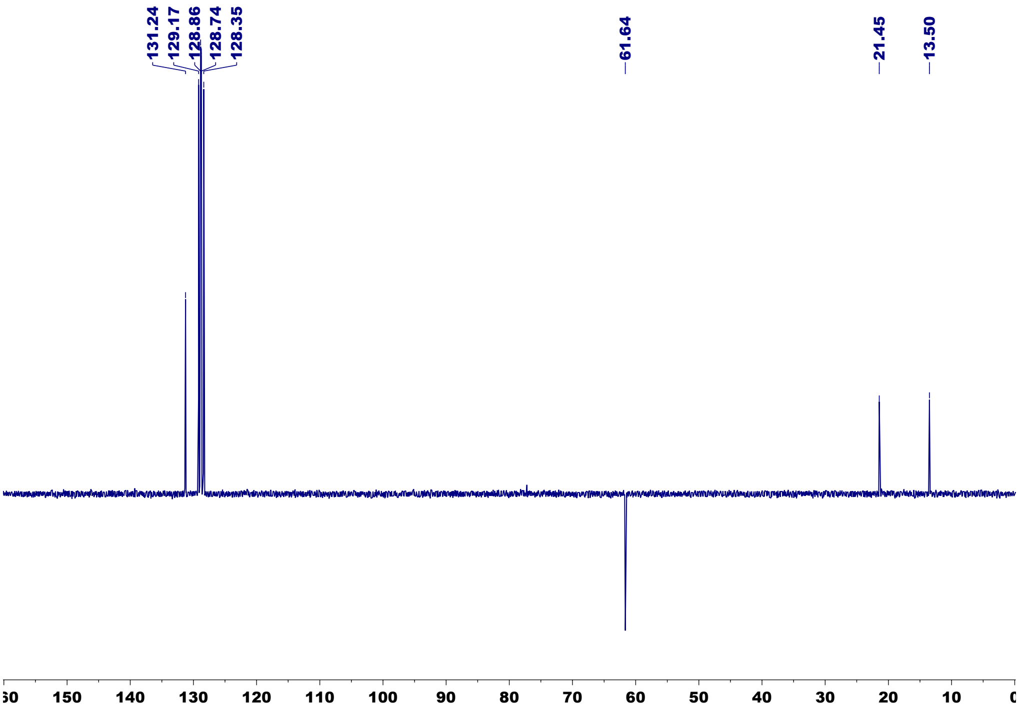
131.24
129.17
128.86
128.74
128.35
61.64
21.45
13.50
150 140 130 120 110 100 90 80 70 60 50 40 30 20 10

3-Benzoyl-4,5-dihydro-2-methyl-4-(4-methylphenyl)-5-nitrofuran (8g) ($CDCl_3$)

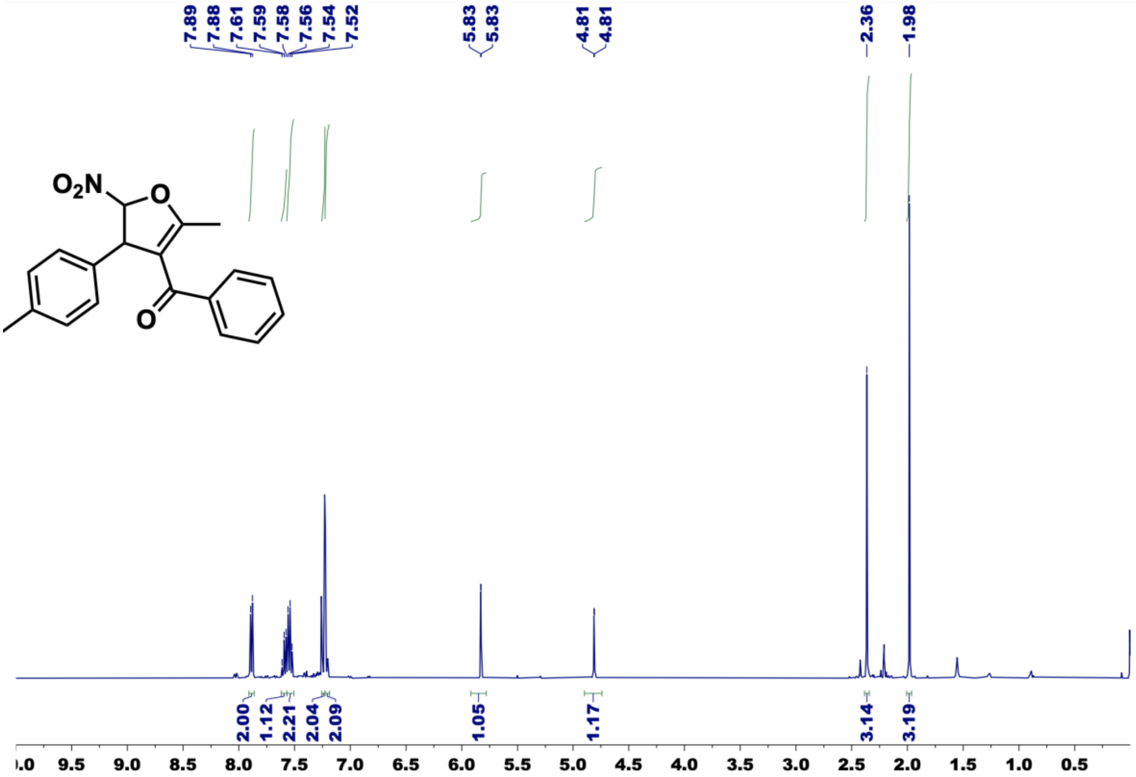

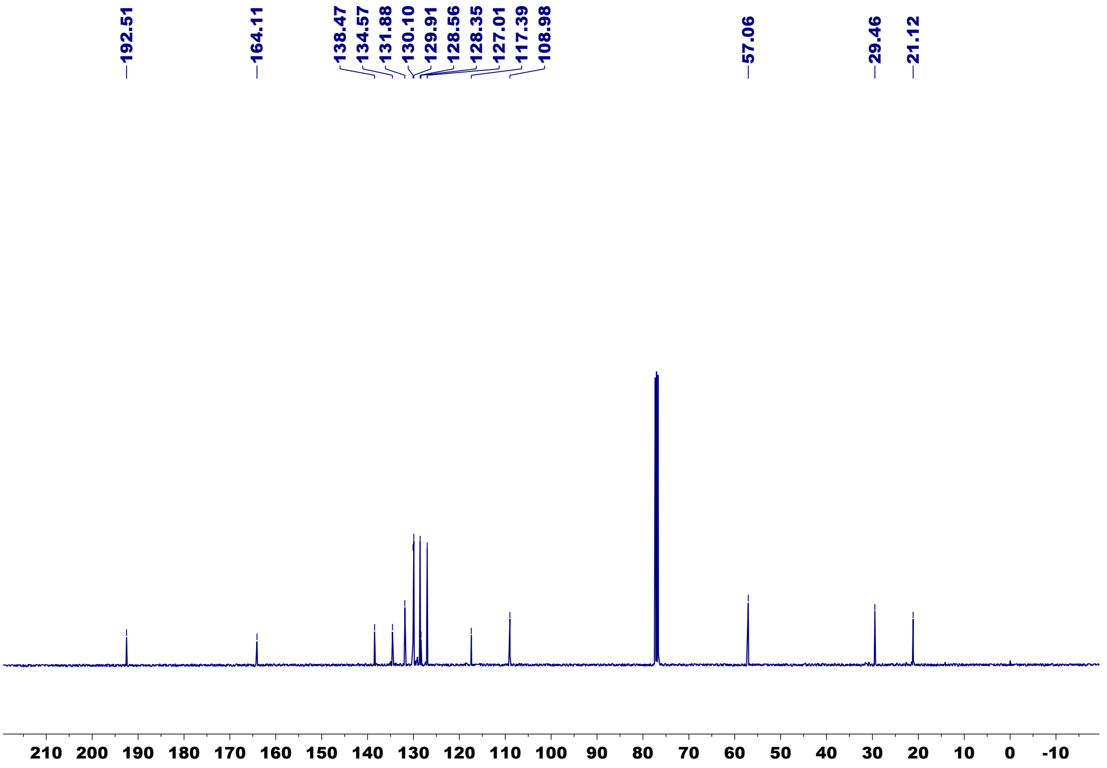

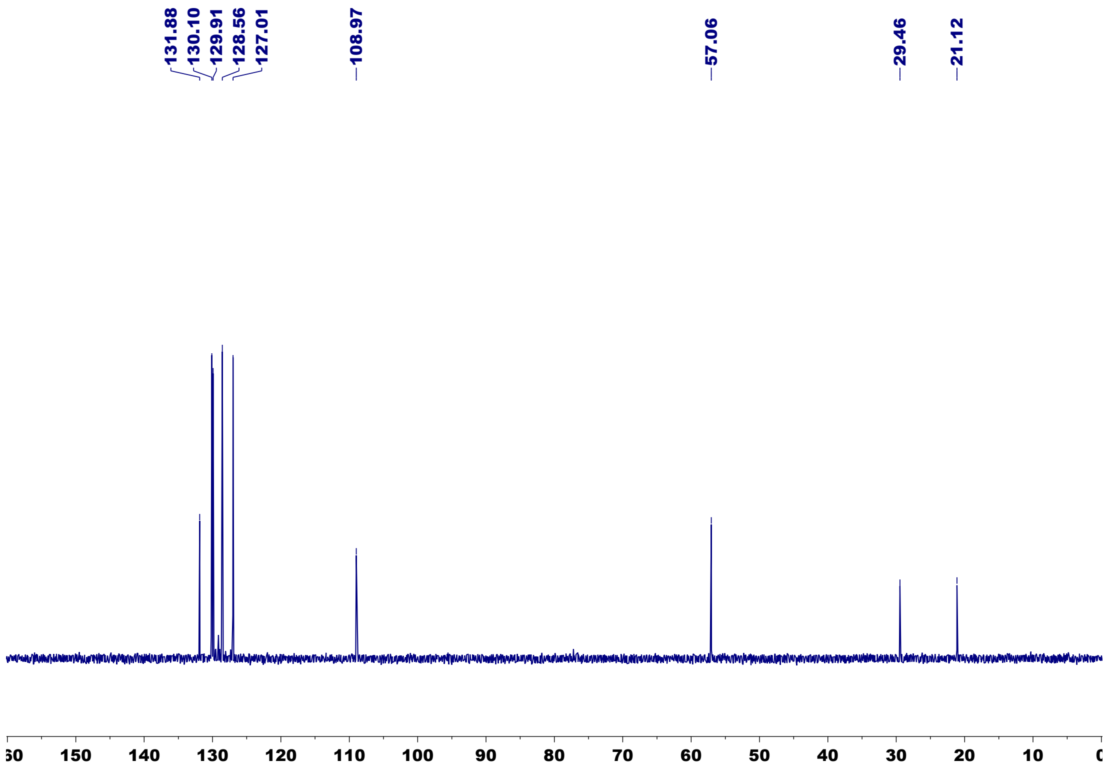

3-Ethanoyl-4,5-dihydro-4-(4-methylphenyl)-2-phenyl-5-nitrofuran (8g') ($CDCl_3$)

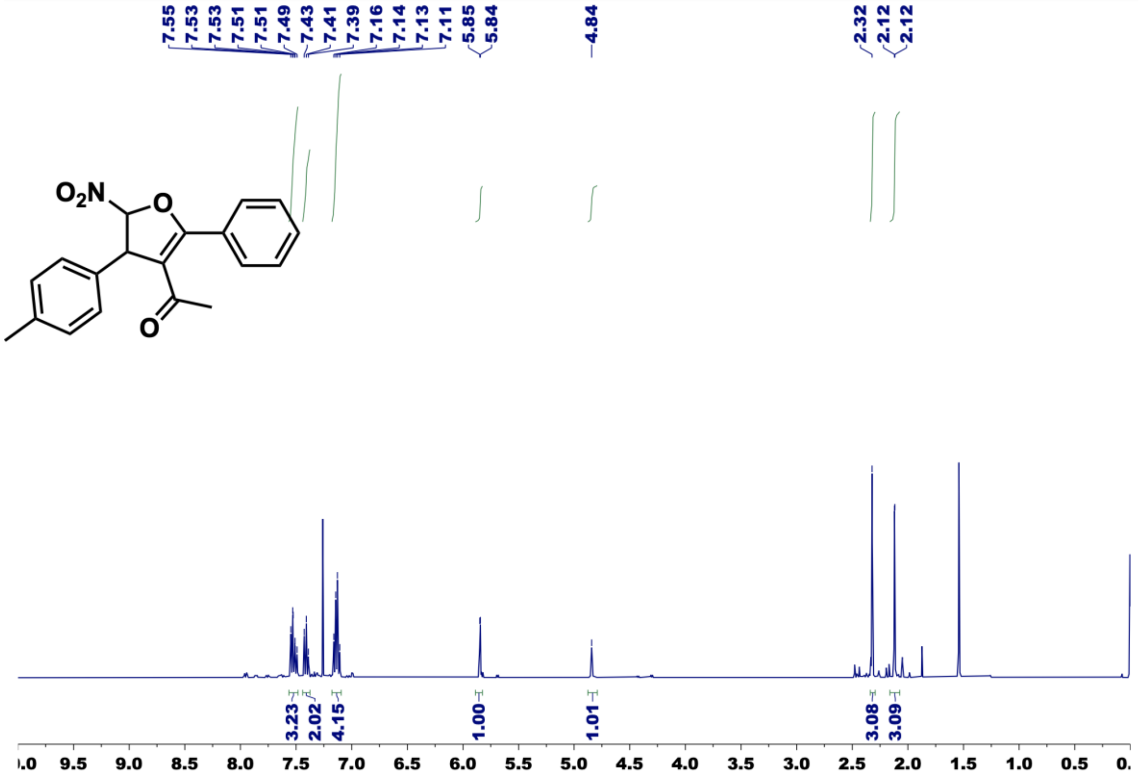

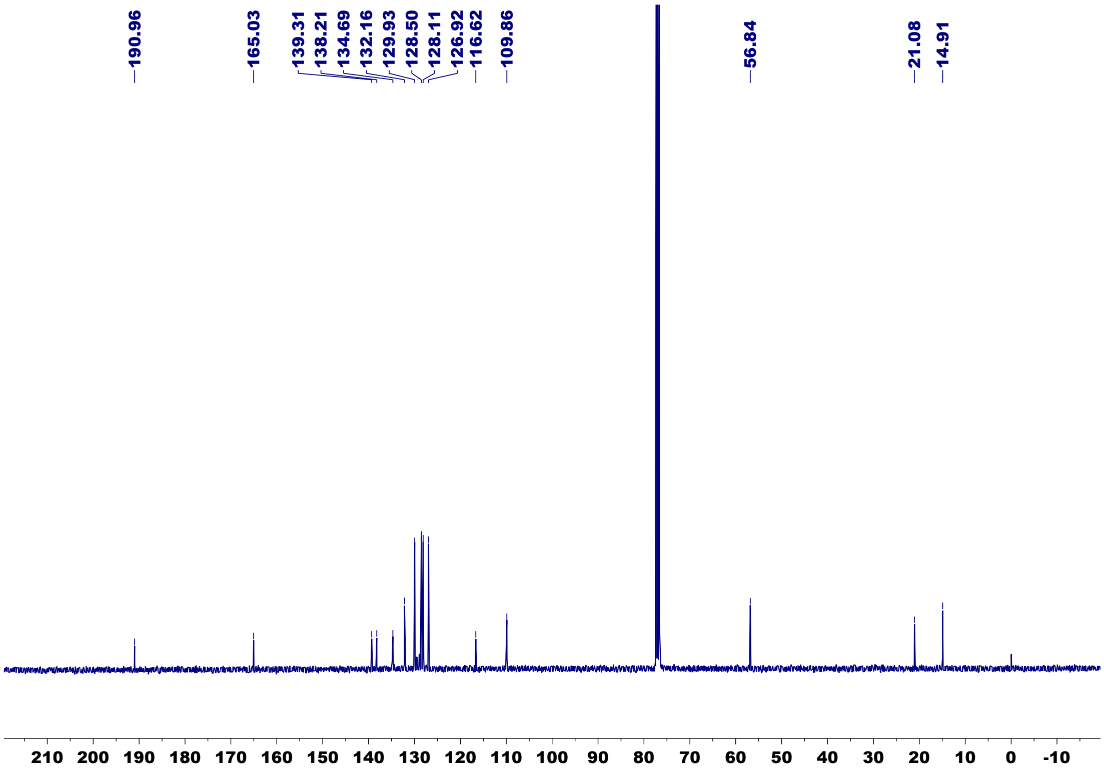
190.96
165.03
139.31
138.21
134.69
132.16
129.93
128.50
128.11
126.92
116.62
109.86
56.84
21.08
14.91
210 200 190 180 170 160 150 140 130 120 110 100 90 80 70 60 50 40 30 20 10 0 -10

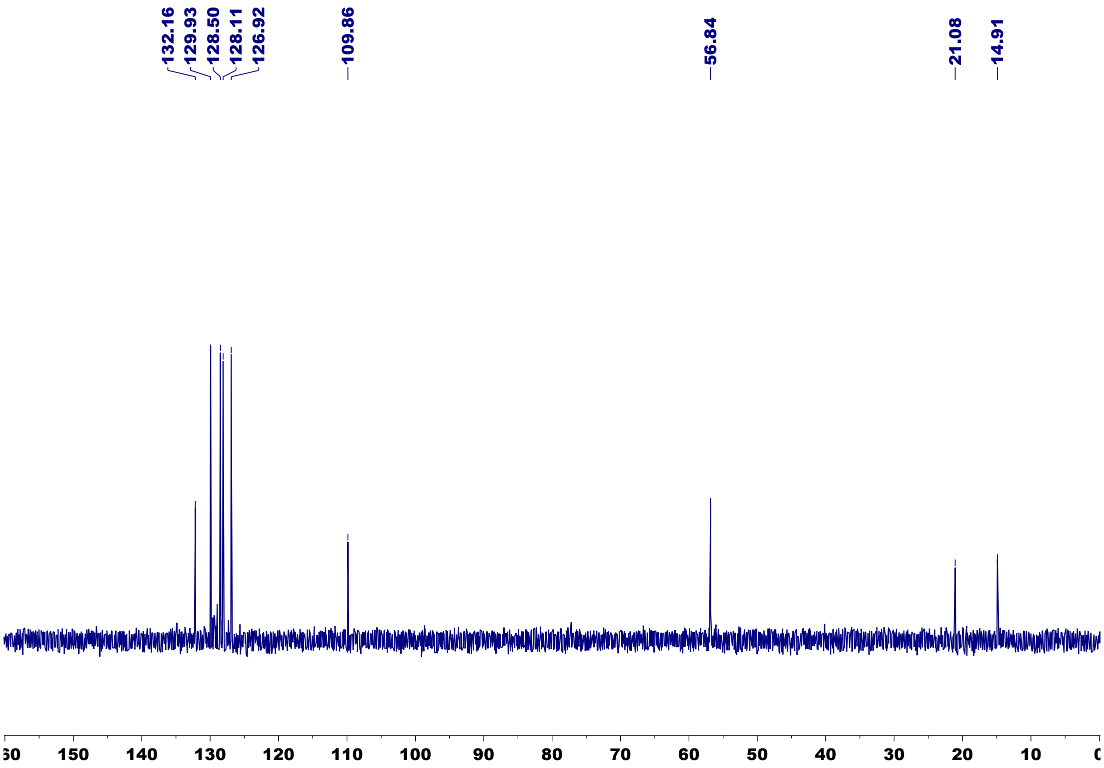
132.16
129.93
128.50
128.11
126.92
109.86
56.84
21.08
14.91
150 140 130 120 110 100 90 80 70 60 50 40 30 20 10

4,5-Dihydro-3-ethanoyl-4-(4-chlorophenyl)-2-methyl-5-nitrofuran (9) ($CDCl_3$)

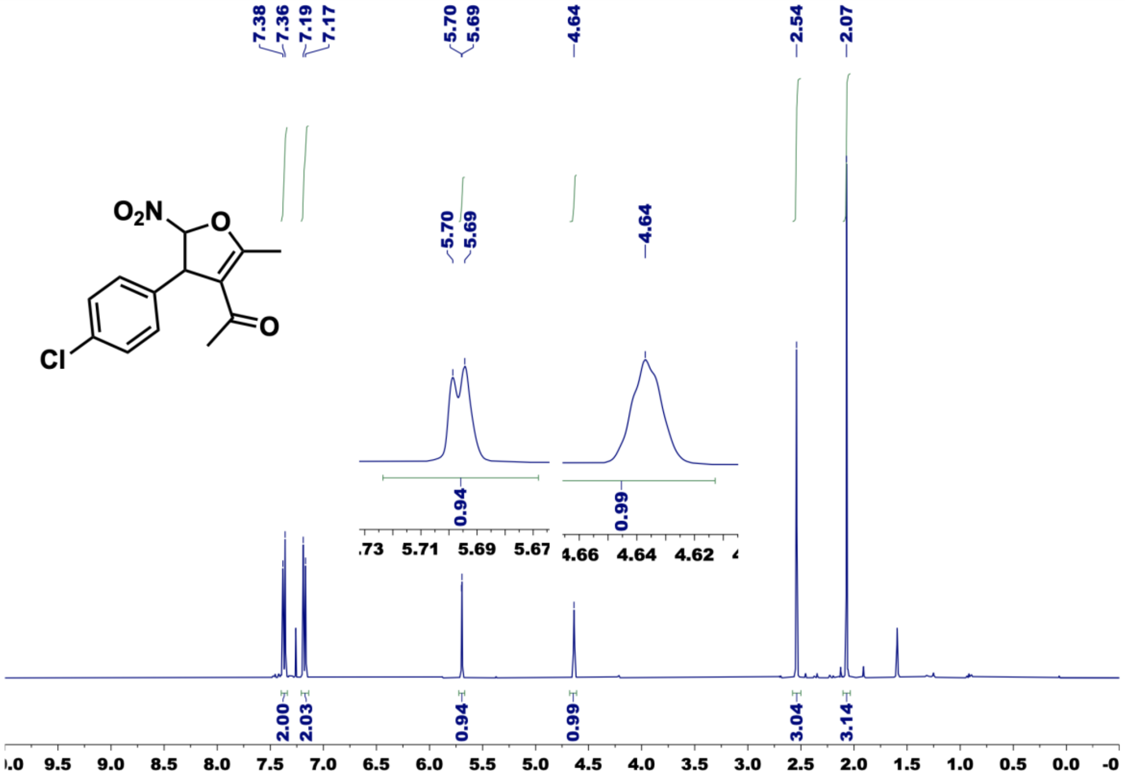

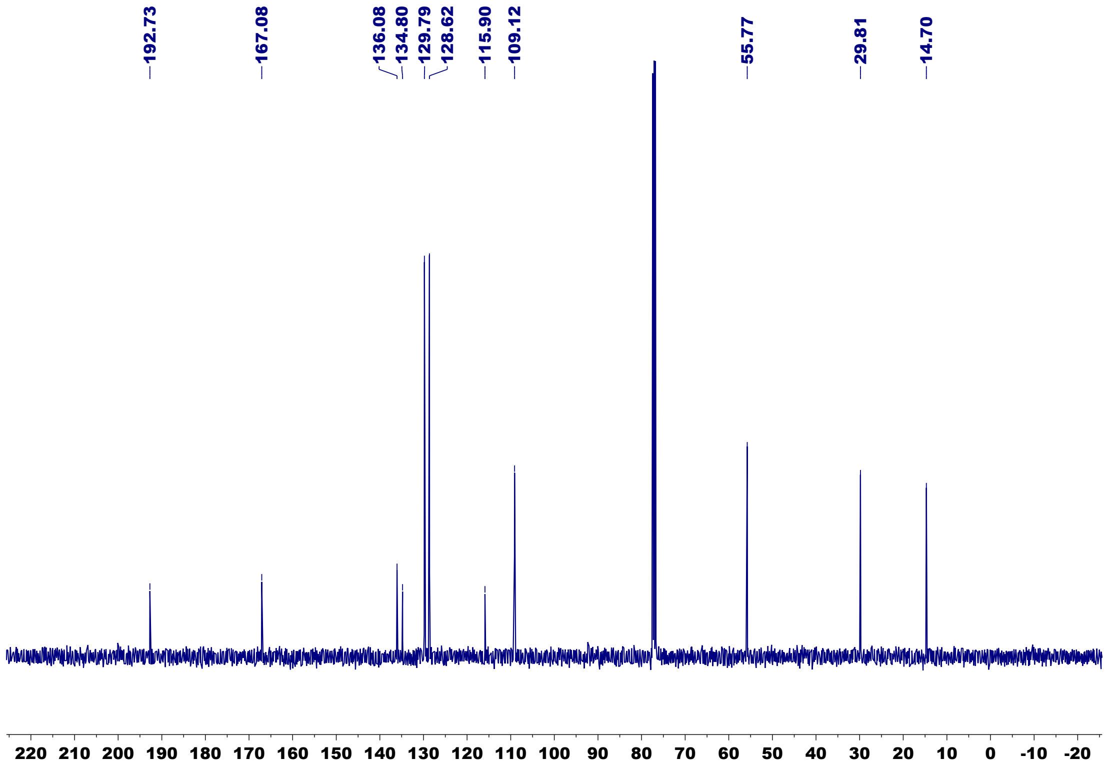

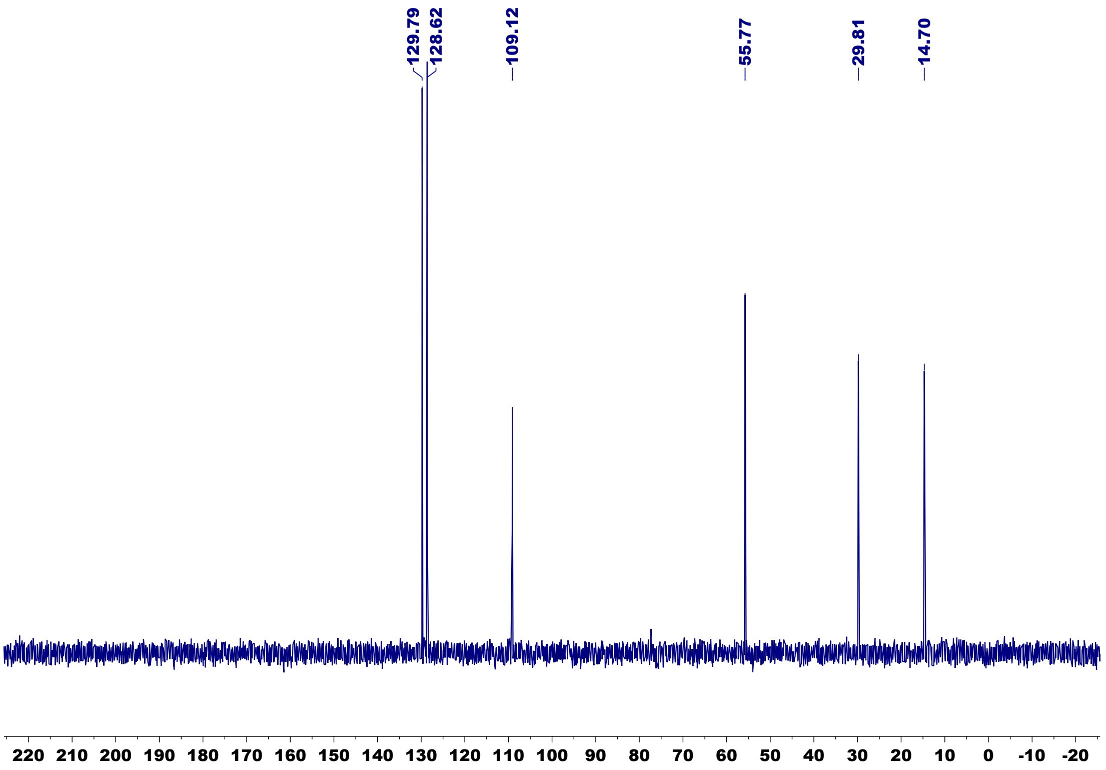

4-Chloro-4,5-dihydro-2-methyl-5-nitro-3-[1-(2,4-dinitrophenylhydrazino)ethyl] furan (10) ($CDCl_3$)

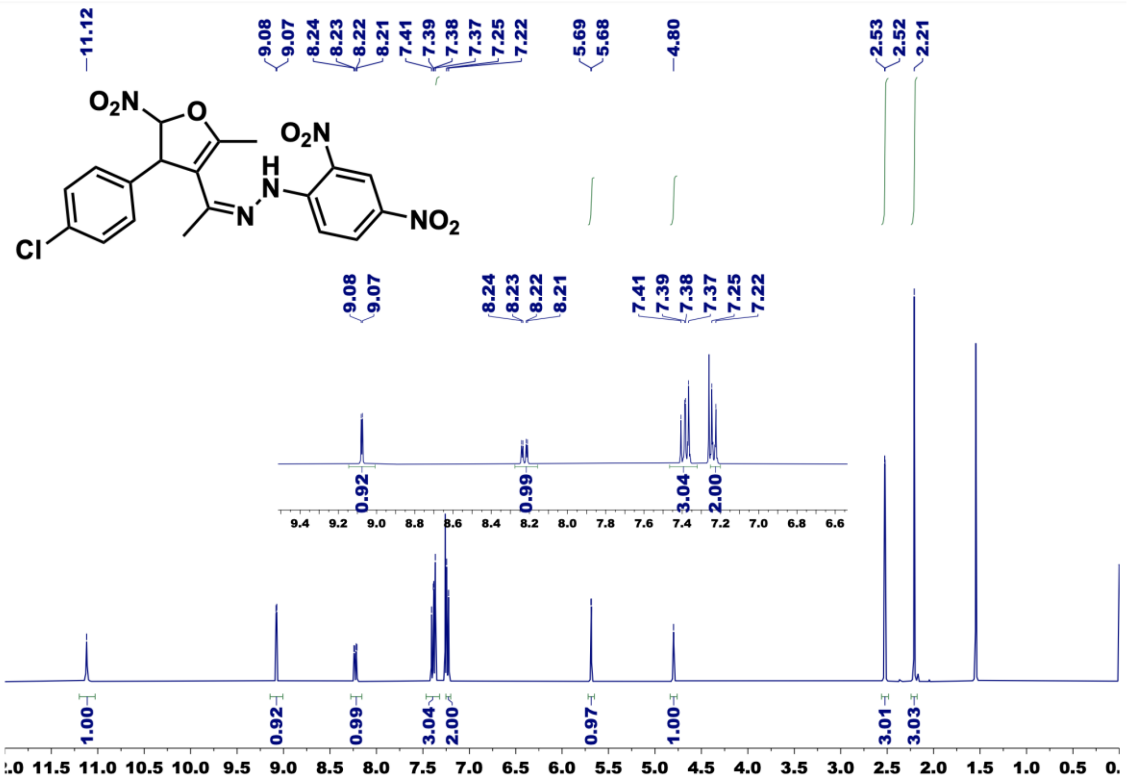

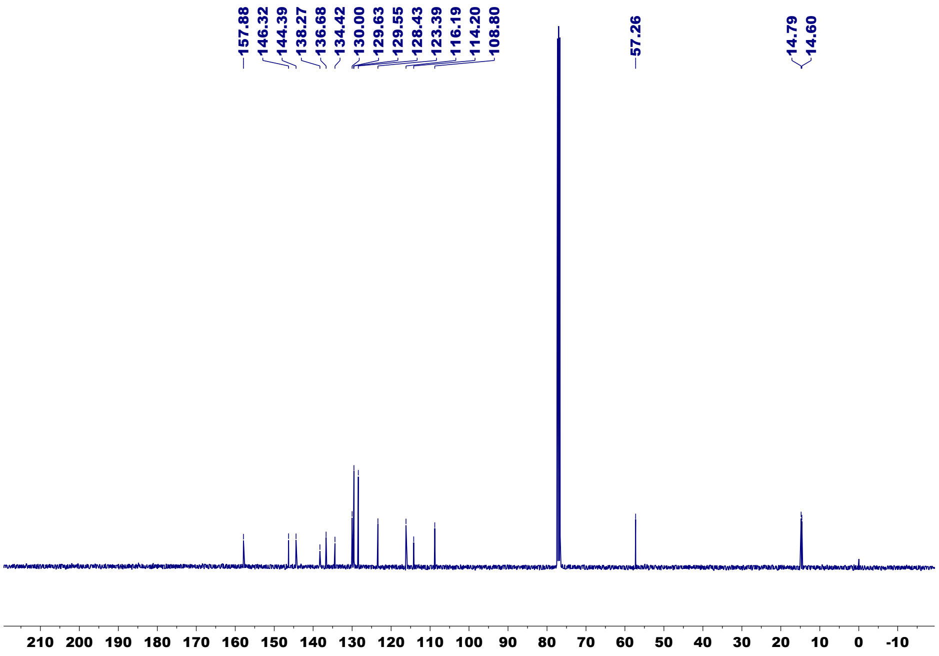
157.88
146.32
144.39
138.27
136.68
134.42
130.00
129.63
129.55
128.43
123.39
116.19
114.20
108.80
57.26
14.79
14.60
210 200 190 180 170 160 150 140 130 120 110 100 90 80 70 60 50 40 30 20 10 0 -10

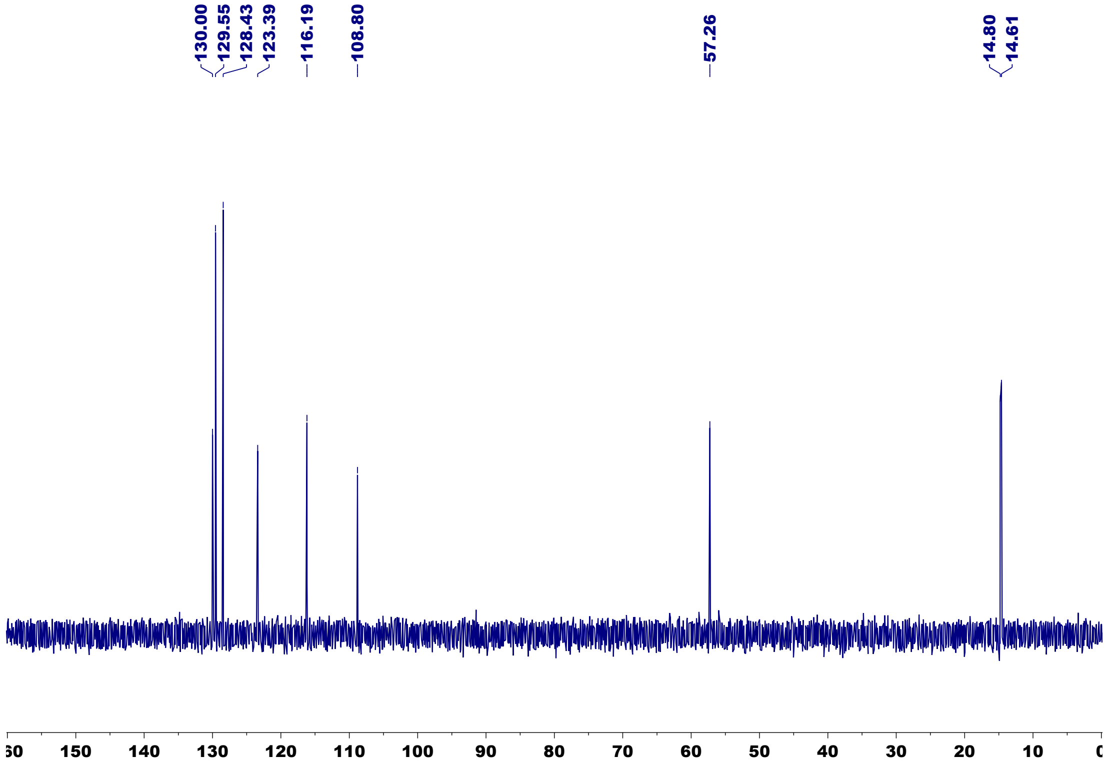
130.00
129.55
128.43
123.39
116.19
108.80
57.26
14.80
14.61
150 140 130 120 110 100 90 80 70 60 50 40 30 20 10

Methyl 4-isopropyl-1-(4-methylphenyl)furan-3-carboxylate (13)

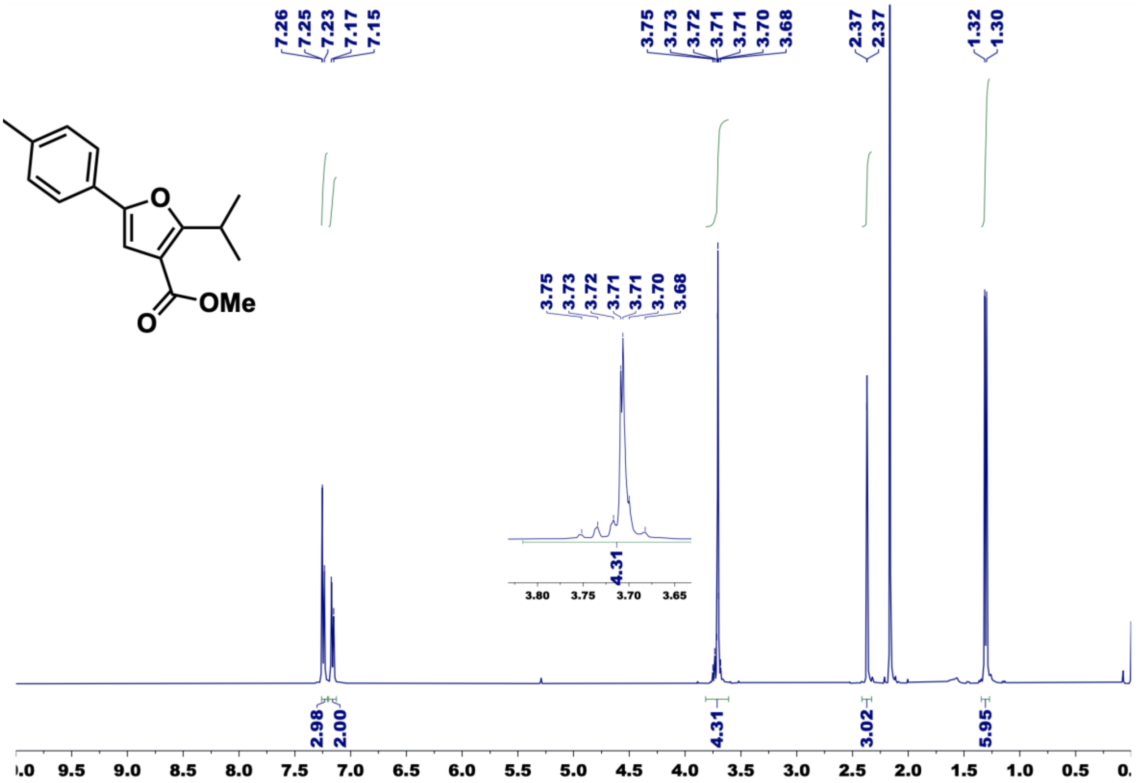

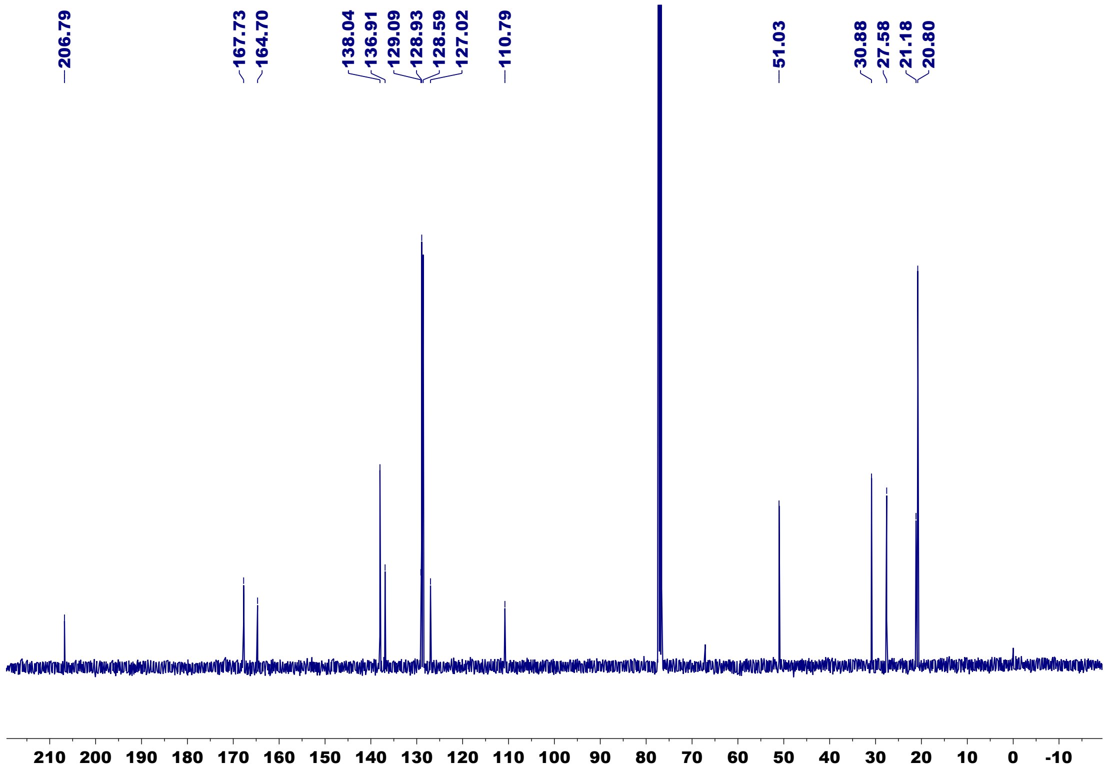

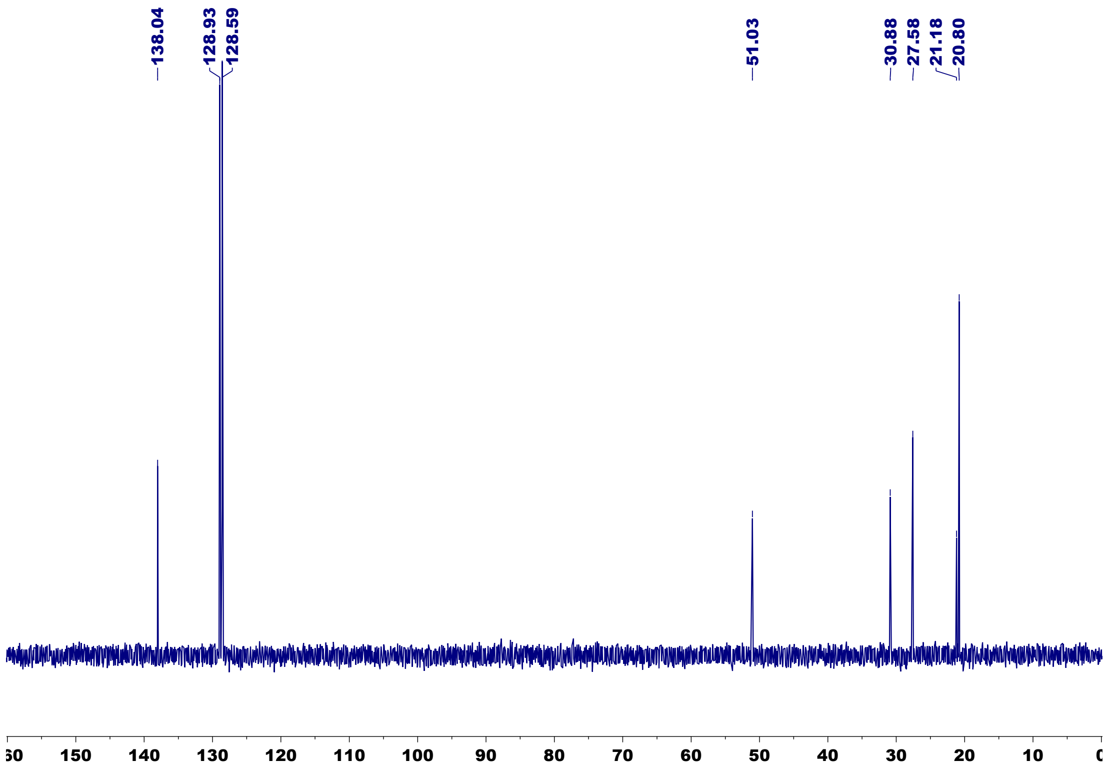

References

1 Li, L.; Babaoglu, E.; Harms, K.; Hilt, G. *Eur. J. Org. Chem.* **2017**, 4543–4547.

2 Zenz, I.; Mayr, H. *J. Org. Chem.* **2011**, *76*, 9370–9378.

3 Srihari, G.; Murthy, M. M. *Synth. Commun.* **2009**, *39*, 896–906.

4 Trukhin, E. V.; Sheremet, E. A.; Masalovich, M. S.; Berestovitskaya, V. M. *Russ. J. Org. Chem.* **2004**, *40*, 1823–1825.

5 Dou, X.; Zhong, F.; Lu, Y. *Chem. Eur. J.* **2012**, *18*, 13945–13948.
