# Supplementary material for: First synthesis of acylated nitrocyclopropanes
Source: Beilstein J Org Chem. 2023 Jun 21;19:892–900. doi: 10.3762/bjoc.19.67 (PMC10291240; doi:10.3762/bjoc.19.67)

BEILSTEIN JOURNAL OF ORGANIC CHEMISTRY

Supporting Information

for

First synthesis of acylated nitrocyclopropanes

Kento Iwai, Rikiya Kamidate, Khimiya Wada, Haruyasu Asahara and Nagatoshi Nishiwaki

Beilstein J. Org. Chem. **2023,** *19,* 892–900. doi:10.3762/bjoc.19.67

CheckCIF/PLATON report for compound 10

License and Terms: This is a supporting information file under the terms of the Creative Commons Attribution License (https://creativecommons.org/licenses/by/4.0). Please note that the reuse, redistribution and reproduction in particular requires that the author(s) and source are credited and that individual graphics may be subject to special legal provisions.
The license is subject to the *Beilstein Journal of Organic Chemistry* terms and conditions: (https://www.beilstein-journals.org/bjoc/terms)

checkCIF/PLATON report

Structure factors have been supplied for datablock(s) RK_a

THIS REPORT IS FOR GUIDANCE ONLY. IF USED AS PART OF A REVIEW PROCEDURE FOR PUBLICATION, IT SHOULD NOT REPLACE THE EXPERTISE OF AN EXPERIENCED CRYSTALLOGRAPHIC REFEREE.

No syntax errors found. CIF dictionary Interpreting this report

Datablock: RK_a

Bond precision:	C-C = 0.0035 A		Wavelength=0.71069
Cell:	a=14.972(5)	b=8.905(5)	c=15.738(3)
	alpha=90	beta=95.65(2)	gamma=90
Temperature:	298 K		

	Calculated	Reported
Volume	2088.1(14)	2088.1(14)
Space group	P 21/n	P 21/n
Hall group	-P 2yn	-P 2yn
Moiety formula	C19 H16 Cl N5 O7	C19 H16 Cl N5 O7
Sum formula	C19 H16 Cl N5 O7	C19 H16 Cl N5 O7
Mr	461.82	461.82
Dx,g cm-3	1.469	1.469
Z	4	4
Mu (mm-1)	0.236	0.236
F000	952.0	952.0
F000'	953.08	
h,k,lmax	19,11,20	19,11,20
Nref	4816	4814
Tmin,Tmax	0.972,0.977	
Tmin'	0.954	

Correction method= Not given

Data completeness= 1.000 Theta(max)= 27.496

R(reflections)= 0.0439(2788) wR2(reflections)= 0.1387(4814)

S = 1.026 Npar= 291

The following ALERTS were generated. Each ALERT has the format
test-name_ALERT_alert-type_alert-level.
Click on the hyperlinks for more details of the test.

Alert level C

```
PLAT230_ALERT_2_C Hirshfeld Test Diff for    O4       --N4       .        5.1 s.u.
PLAT905_ALERT_3_C Negative K value in the Analysis of Variance ...     -1.618 Report
```

Alert level G

```
PLAT007_ALERT_5_G Number of Unrefined Donor-H Atoms .............          1 Report
PLAT398_ALERT_2_G Deviating  C-O-C   Angle From 120 for O1       .      107.3 Degree
PLAT431_ALERT_2_G Short Inter HL..A Contact  Cl1      ..O4       .       3.03 Ang.
                                        -1/2+x,1/2-y,1/2+z  =      4_566 Check
PLAT793_ALERT_4_G Model has Chirality at C1          (Centro SPGR)          R Verify
PLAT793_ALERT_4_G Model has Chirality at C2          (Centro SPGR)          R Verify
PLAT910_ALERT_3_G Missing # of FCF Reflection(s) Below Theta(Min).          2 Note
PLAT941_ALERT_3_G Average HKL Measurement Multiplicity ..........         1.0 Low
PLAT978_ALERT_2_G Number C-C Bonds with Positive Residual Density.          2 Info
```

0 **ALERT level A** = Most likely a serious problem - resolve or explain
0 **ALERT level B** = A potentially serious problem, consider carefully
2 **ALERT level C** = Check. Ensure it is not caused by an omission or oversight
8 **ALERT level G** = General information/check it is not something unexpected

0 ALERT type 1 CIF construction/syntax error, inconsistent or missing data
4 ALERT type 2 Indicator that the structure model may be wrong or deficient
3 ALERT type 3 Indicator that the structure quality may be low
2 ALERT type 4 Improvement, methodology, query or suggestion
1 ALERT type 5 Informative message, check

It is advisable to attempt to resolve as many as possible of the alerts in all categories. Often the minor alerts point to easily fixed oversights, errors and omissions in your CIF or refinement strategy, so attention to these fine details can be worthwhile. In order to resolve some of the more serious problems it may be necessary to carry out additional measurements or structure refinements. However, the purpose of your study may justify the reported deviations and the more serious of these should normally be commented upon in the discussion or experimental section of a paper or in the "special_details" fields of the CIF. checkCIF was carefully designed to identify outliers and unusual parameters, but every test has its limitations and alerts that are not important in a particular case may appear. Conversely, the absence of alerts does not guarantee there are no aspects of the results needing attention. It is up to the individual to critically assess their own results and, if necessary, seek expert advice.

Publication of your CIF in IUCr journals

A basic structural check has been run on your CIF. These basic checks will be run on all CIFs submitted for publication in IUCr journals (*Acta Crystallographica*, *Journal of Applied Crystallography*, *Journal of Synchrotron Radiation*); however, if you intend to submit to *Acta Crystallographica Section C* or *E* or *IUCrData*, you should make sure that full publication checks are run on the final version of your CIF prior to submission.

Publication of your CIF in other journals

Please refer to the *Notes for Authors* of the relevant journal for any special instructions relating to CIF submission.

PLATON version of 28/11/2022; check.def file version of 28/11/2022

Datablock RK_a - ellipsoid plot

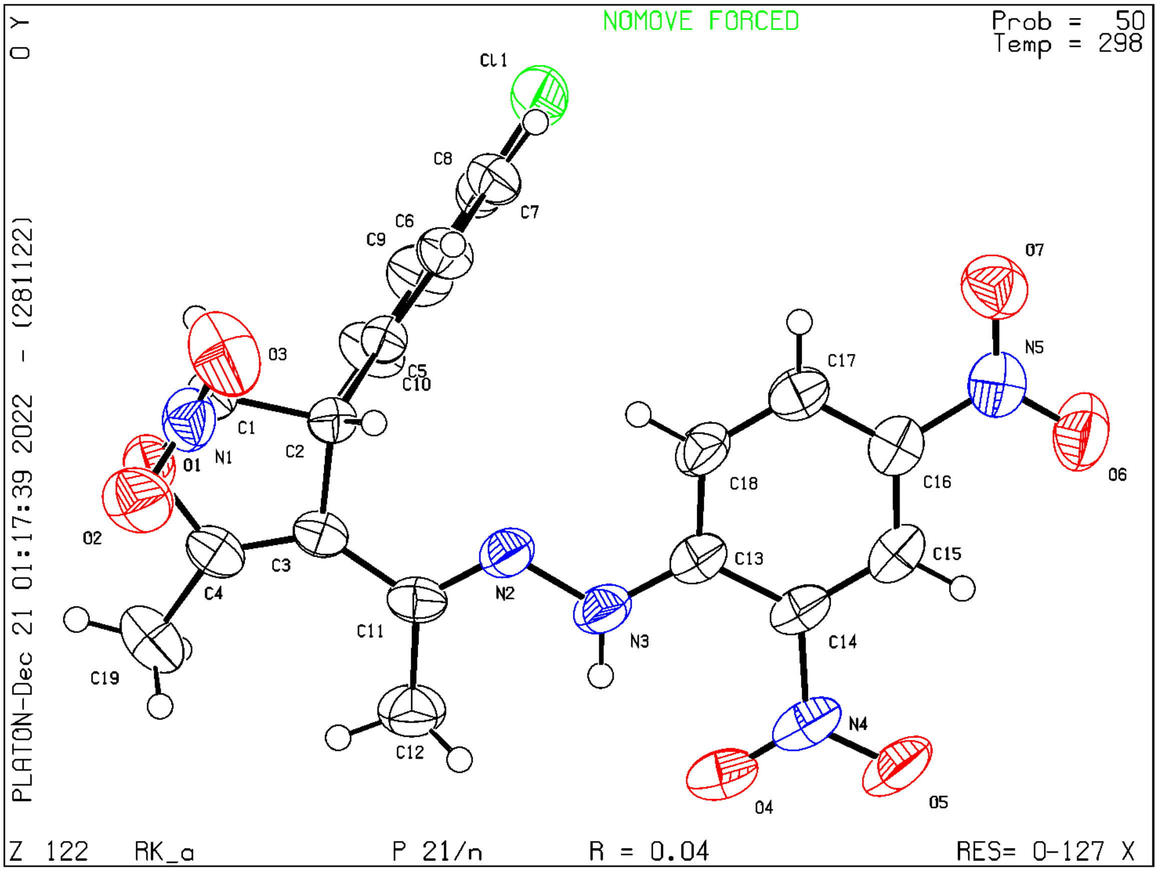

Supplement: File 3 — CheckCIF/PLATON report for compound 10. [file Beilstein_J_Org_Chem-19-892-s003.pdf]
